# Platelet Aggregation Unchanged by Lipoprotein-Associated Phospholipase A_2_ Inhibition: Results from an In Vitro Study and Two Randomized Phase I Trials

**DOI:** 10.1371/journal.pone.0083094

**Published:** 2014-01-27

**Authors:** Bonnie C. Shaddinger, Yanmei Xu, James H. Roger, Colin H. Macphee, Malcolm Handel, Charlotte A. Baidoo, Mindy Magee, John J. Lepore, Dennis L. Sprecher

**Affiliations:** 1 Discovery Medicine, Heart Failure Discovery Performance Unit, Metabolic Pathways and Cardiovascular Therapeutic Area, GlaxoSmithKline, King of Prussia, Pennsylvania, United States of America; 2 Discovery Biometrics, GlaxoSmithKline, King of Prussia, Pennsylvania, United States of America; 3 Research Statistics Unit, GlaxoSmithKline, Stockley Park, United Kingdom; 4 Janssen-Cilag Pty Ltd, Macquarie Park, New South Wales, Australia; New York University School of Medicine, United States of America

## Abstract

**Background:**

We explored the theorized upregulation of platelet-activating factor (PAF)– mediated biologic responses following lipoprotein-associated phospholipase A_2_ (Lp-PLA_2_) inhibition using human platelet aggregation studies in an in vitro experiment and in 2 clinical trials.

**Methods and Results:**

Full platelet aggregation concentration response curves were generated in vitro to several platelet agonists in human plasma samples pretreated with rilapladib (selective Lp-PLA_2_ inhibitor) or vehicle. This was followed by a randomized, double-blind crossover study in healthy adult men (n = 26) employing a single-agonist dose assay of platelet aggregation, after treatment of subjects with 250 mg oral rilapladib or placebo once daily for 14 days. This study was followed by a second randomized, double-blind parallel-group trial in healthy adult men (n = 58) also treated with 250 mg oral rilapladib or placebo once daily for 14 days using a full range of 10 collagen concentrations (0–10 µg/ml) for characterizing EC_50_ values for platelet aggregation for each subject. Both clinical studies were conducted at the GlaxoSmithKline Medicines Research Unit in the Prince of Wales Hospital, Sydney, Australia. EC_50_ values derived from multiple agonist concentrations were compared and no pro-aggregant signals were observed during exposure to rilapladib in any of these platelet studies, despite Lp-PLA_2_ inhibition exceeding 90%. An increase in collagen-mediated aggregation was observed 3 weeks post drug termination in the crossover study (15.4% vs baseline; 95% confidence interval [CI], 3.9–27.0), which was not observed during the treatment phase and was not observed in the parallel-group study employing a more robust EC_50_ examination.

**Conclusions:**

Lp-PLA_2_ inhibition does not enhance platelet aggregation.

**Trial Registration:**

1) Study 1: ClinicalTrials.gov NCT01745458 2) Study 2: ClinicalTrials.gov NCT00387257

## Introduction

Potent, selective, and orally active lipoprotein-associated phospholipase A_2_ (Lp-PLA_2_) inhibitors are in clinical development to determine their effects on reducing the risk of atherothrombosis and associated clinical sequelae (eg, acute coronary syndromes, ischemic stroke, etc) [1]–[4]. However, Lp-PLA_2_ was initially described as platelet-activating factor (PAF) acetylhydrolase (PAF-AH) because of its ability to hydrolyze PAF in vitro into biologically inactive lyso-PAF [5]–[7], when PAF in micromolar concentrations is added to isolated human plasma, [8]. Thus, it has been postulated that Lp-PLA_2_ inhibition may enhance PAF-mediated biology. Based on its role in platelet function, PAF accumulation could theoretically increase platelet aggregation, potentially contributing to adverse thrombotic events in the target population for which Lp-PLA_2_ inhibitors are being developed [9], [10].

Published evidence to date offers mixed results regarding the role of Lp-PLA_2_ activity in influencing PAF-mediated biology. Although relatively little direct evidence has been published [11], [12], multiple studies have reported positive associations between plasma Lp-PLA_2_ and various disorders in which PAF has been implicated, including asthma, allergic reactions, and anaphylaxis [13]–[18]. Conceptually, endogenous pathways for hydrolysis of PAF may potentially ameliorate PAF's otherwise inflammatory influence. As a result, understanding the relationship between Lp-PLA_2_ and PAF-mediated responses has been a critical step for advancing the clinical development of Lp-PLA_2_ inhibitors (darapladib and rilapladib) and initiating large-scale cardiovascular outcomes trials [19], [20].

Both darapladib and rilapladib are potent (half maximal inhibitory concentrations of 270 pM and 230 pM, respectively) and selective Lp-PLA_2_ inhibitors [1]. As such, the highest clinical dose of darapladib (80 mg nonenteric coated or 160 mg enteric coated) can achieve approximately 80% inhibition of plasma Lp-PLA_2_ 24 hours post dose. The top clinical dose of rilapladib (250 mg enteric coated) can attain an even greater magnitude of inhibition of around 90% at the same 24-hour postdose time point, with earlier time points demonstrating essentially complete inhibition. Therefore, rilapladib represents a more robust inquiry into the relevance of Lp-PLA_2_ inhibition to PAF-mediated biology and targets a more substantive physiology related to vascular disease. Thus, the purpose of the studies described herein was to explore the effects of the potent Lp-PLA_2_ inhibitor, rilapladib, on PAF-mediated biologic responses—in particular, platelet aggregation.

## Methods

The protocol for these trials and supporting CONSORT checklist are available as supporting information; see Checklist S1 and Protocol S1.

### In Vitro Platelet Aggregation Study

This study was conducted at GlaxoSmithKline, Upper Merion, PA, between March 15^th^ and July 18^th^, 2005. Blood donors provided written informed consent to have their blood used for research, and this procedure was approved by the GlaxoSmithKline safety board. Platelet-rich plasma (PRP) was prepared from the blood of 10 healthy human volunteers by centrifugation at 220 g for 15 minutes (females were excluded due to possible confounding of menstrual cycles and/or estrogen therapies). After the removal of PRP, samples were centrifuged at 1500 g for 10 minutes to obtain platelet-poor plasma (PPP). Platelet-rich plasma samples were adjusted to 250,000 platelets/µL using autologous PPP. Light transmission aggregometry was performed using Chronolog aggregometers (Model 570-VS, Chronolog Corporation, Havertown, PA) using 100% PPP as a reference. Platelet-rich plasma samples were pretreated with 100 nM rilapladib or vehicle (DMSO) for 5 minutes prior to addition of the platelet agonist. For each donor, 5 concentrations of each platelet agonist were used (adenosine diphosphate [ADP] = 0.5 µM, 1 µM, 2 µM, 3 µM, and 5 µM; collagen = 0.0625 µg/mL, 0.125 µg/mL, 0.25 µg/mL, 0.5 µg/mL, and 1 µg/mL; platelet-activating factor [PAF] = 3 nM, 10 nM, 30 nM, 100 nM, and 300 nM).The percent aggregation associated with each agonist concentration was used to generate an aggregation curve for each donor sample, and the average of the aggregation curves from all donors for each agonist was used to identify the concentration associated with EC_50_. Statistical significance for all results was assessed by a paired t-test. Lp-PLA_2_ activity was measured in parallel using plasma samples that were derived from PRP that had been treated either with vehicle or rilapladib (100 nM) for 5 minutes using [^3^H]-PAF as the substrate as previously described [21]. Duplicate data were collected from each individual donor for each treatment/agonist concentration tested. Platelet aggregation was reported as the maximum aggregation measured within 8 minutes of agonist addition (mean ± standard error [SE]).

### Ex Vivo Platelet Aggregation Study 1: Crossover study using a single agonist-dose assay

#### Subjects, Study Design, and Conduct

**Figure 1:**
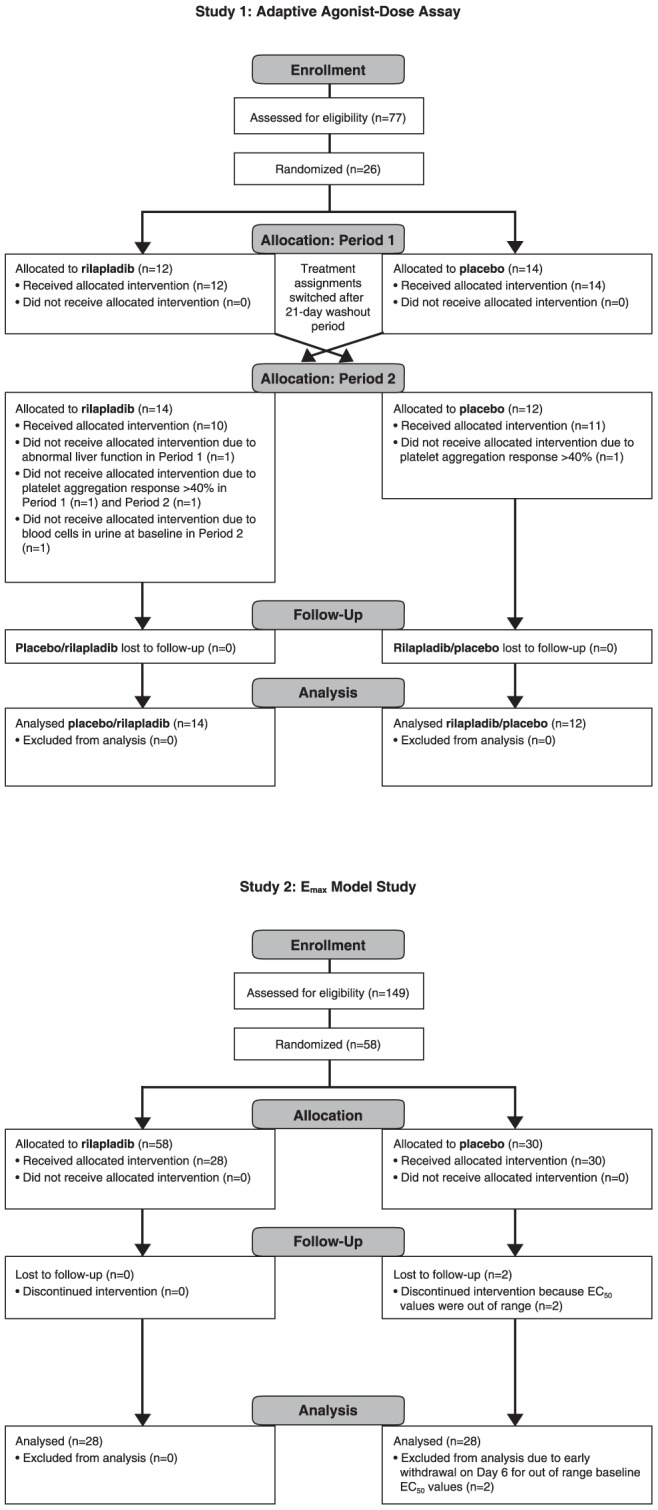
Flow diagram depicting (A) single agonist-dose and (B) E_max_ model.

This was a randomized, double-blind, placebo-controlled, repeat-dose, crossover study involving 26 otherwise healthy adult men (19–48 years of age) (Figure 1A; ClinicalTrials.gov identifiers: NCT01745458 and NCT01750827). This study was conducted at the GlaxoSmithKline Medicines Research Unit in the Prince of Wales Hospital, Sydney, Australia. Subjects provided written, informed consent and the protocol was approved by the Human Research Ethics Committee in Sydney, Australia. Subjects were enrolled in the study between July 20, 2005, and September 29, 2005. This study was conducted in accordance with Good Clinical Practice Guidelines and guiding principles of the Declaration of Helsinki [22].

The study was set up using an estimation approach, while the point estimate and associated confidence intervals were provided to describe any potential treatment difference. Sample size was chosen based on feasibility, with the power analysis conducted to put the sample size into the context of the statistical comparison. Based on the within-subject variability estimate (SD = 15.6%, maximum platelet aggregation using collagen as an agonist) obtained from the preliminary analyses of a previous nonrandomized single cohort study on 14 subjects, it was calculated that a sample size of 20 subjects would provide at least 90% power to detect a difference of 15% between rilapladib and placebo using a two-sided t-test with a type I error rate of 10%. Allocation of eligible subjects was determined using a computer-generated randomization schedule, and a 1∶1 allocation ratio between 2 treatment sequences (active then placebo or placebo then active). Subject numbers were allocated to eligible subjects in sequential order. Rilapladib and placebo were in matching tablet form. The pharmacy at the investigative site was responsible for allocation and dispensing. The drug supply to the site was in treatment packs labeled as period 1 and period 2 for each individual subject. Subjects, physicians, and site staff associated with the study as well as GlaxoSmithKline teams were blinded and unaware of the allocated treatment with the exception of an unblinded pharmacist at the investigative site. The pharmacist had no direct subject contact during the trial.

Study participants were prohibited from using prescription or over-the-counter medications within 7 days or 5 half-lives (whichever was longer) prior to the study and through the follow-up visit. Anticoagulants, antiplatelets, aspirin, aspirin-containing products, or nonsteroidal anti-inflammatory agents were prohibited within 14 days before the study and through the follow-up visit. Dietary/herbal supplements including (but not limited to) St. John's wort, kava, ephedra (ma huang), gingko biloba, DHEA, yohimbe, saw palmetto, ginseng, and red yeast rice were prohibited within 14 days prior to the study. Acetaminophen (≤2 g/d) was permitted up to 48 hours prior to dosing. A total of 4 study participants took medications routinely prohibited in healthy volunteer physiology studies, including antiallergy medications (eg, cetirizine, loratadine), analgesics (propoxyphene, dextropropoxyphene+acetaminophen), and common cold medications (eg, pseudoephedrine, povidone-iodine throat gargle solution).These medications were not expected to affect subject safety or the results of the study.

Subjects were randomly assigned to receive rilapladib (SB-659032) 250 mg (non–enteric coated formulation) or matching placebo once daily, following a low-fat breakfast, for 14 days. Upon completion of the first 14 days of treatment and a 21-day washout period (first study phase), the treatment assignments were switched, in a crossover fashion, for another 14 days of treatment, with a follow-up visit 21 days later. Therefore, the initiation of period 2 is the last follow-up day for period 1. Blood samples were collected at 0, 6, and 24 hours pre dose and post dose (respectively) on day 1 and again on day 14, and at comparable 0-, 6-, and 24-hour testing time points on day −1 and day 35 of each treatment period (when no actual drug was administered, but time-equivalent testing was performed). The “0” time point is synonymous with the 24-hour time point of the day before (eg, day −1).

#### Assessments and End Points

Platelet aggregation tests were performed using 2 ADP concentrations (2 µM and 10 µM) and 4 collagen concentrations (0.7 µg/mL, 1.0 µg/mL, 1.2 µg/mL, and 10 µg/mL).The highest concentrations, 10 uM for ADP and 10 µg/mL for collagen, were used as the positive control. A single, participant-specific subthreshold collagen concentration was defined as the concentration (of the above) that achieved an aggregation response of less than 40% in an individual participant (established at initiation of both study periods). All platelet aggregation assessments (other than those performed as the positive control) were performed in duplicate. Additional testing was mandated if the 2 results were disparate (>10% apart). If none of the agonist doses resulted in less than 40% aggregation at baseline for either study period, the subject was withdrawn from the study. Lp-PLA_2_ activity was measured in parallel using [^3^H]-PAF as the substrate as previously described [23].

#### Statistical Methods

Data from agonists ADP and collagen were analyzed separately. The average of replicates at each time point was calculated and the derived average (sub-maximum concentration) was chosen for collagen from the low or middle concentrations (0.7 µg/mL, 1.0 µg/mL, 1.2 µg/mL). The sub-maximum concentration was determined from the baseline (day −1) data for each subject at each time point within each period independently. If, for example, the average aggregation from the baseline middle concentration at 6 hours was less than 40% for one subject, then the sub-maximum concentration would be based on the middle concentration for the 6-hour post-dose time points on all subsequent study days within that same period for that subject. After the choice of sub-maximum concentration data, baseline corrected percent maximum platelet aggregation data were derived by subtracting the baseline aggregation from the post-dose platelet aggregation for corresponding time points (eg, day 14, 6 hours - day 1, 6 hours).

The primary end point, change from baseline in maximum platelet aggregation following ADP (at 2 µM), was analyzed separately by a mixed-effects ANOVA model, fitting fixed terms for regimen, period, day, time, an interaction term for regimen*day*time, and subject as a random effect. Baseline was included as a covariate in the model. Point estimates and 90% confidence intervals for the difference in active-placebo at each time point on days 1 and 14 and the follow-up (21 days after the last dose of the study drug) were calculated. For collagen, the above analyses were performed based on a priori collagen concentration selection rules (ie, sub-maximum concentration).

### Ex Vivo Platelet Aggregation Study 2: Emax model

#### Subjects, Study Design, and Conduct

This was a randomized, double-blind, placebo-controlled, repeat-dose, parallel-group study (ClinicalTrials.gov identifier: NCT00387257) involving 58 healthy (based on medical history, physical examination, laboratory tests, and cardiac monitoring) adult male subjects (aged 18–55 years; Figure 1B). This study was conducted at the GlaxoSmithKline Medicines Research Unit in the Prince of Wales Hospital, Sydney, Australia. Subjects were enrolled in the study between October 9, 2006, and March 5, 2007. Subjects provided written, informed consent, and the protocol was approved by South East Health Institutional Review Board. This study was conducted in accordance with Good Clinical Practice Guidelines and guiding principles of the Declaration of Helsinki [22].

Major exclusion criteria included the use of aspirin, aspirin-containing products, nonsteroidal anti-inflammatory agents, or any other antiplatelet medication within 14 days prior to the first platelet aggregation assessment; use of prescription or nonprescription drugs within 7 days of randomization (or 14 days if the agent is a potential enzyme inducer); and use of dietary/herbal supplements including (but not limited to) St. John's wort, kava, ephedra (ma huang), gingko biloba, DHEA, yohimbe, saw palmetto, ginseng, or red yeast rice within 14 days prior to randomization. The use of acetaminophen at doses less than or equal to 2 g/d was permitted. Subjects were excluded if their blood samples did not aggregate fully (ie, ≥40%) in response to a collagen concentration of 10 µg/mL at any point between screening and day −1, or if they had an out-of-range EC_50_ value on day −1 (ie, EC_50_<0.4 µg/mL or >1.5 µg/mL at either the predose or 6-hour postdose aggregation test on day −1).

Allocation of eligible subjects was determined using a computer-generated randomization list, and with a 1∶1 active to placebo allocation. Rilapladib and placebo were in matching tablet form. An unblinded pharmacist at the investigative site was responsible for preparation of blinded subject packs according to the randomization schedule for allocation to the subject. The subject packs were then allocated by investigator site staff in sequential order to eligible participants. Subjects, physicians, and site staff associated with the study as well as GlaxoSmithKline teams were blinded and unaware of the allocated treatment with the exception of the unblinded pharmacist at the investigative site. The pharmacist had no direct subject contact during the trial.

Eligible subjects were enrolled and randomly assigned to receive 250 mg of rilapladib or placebo daily for 14 days. Platelet aggregation was measured by light transmission aggregometry on day −1 (0 and 6 hours; latter time point corresponding to maximum concentration [C_max_] of rilapladib), day 1 (pre dose and 6 hours post dose), and day 14 (pre dose and 6 hours post dose), and at comparable time points 21 days after the last dose (day 35). Platelet aggregation responses were assessed for each study participant at each time point, using 8 levels of collagen concentration (0.3 µg/mL, 0.4 µg/mL, 0.6 µg/mL, 0.8 µg/mL, 1.0 µg/mL, 1.2 µg/mL, 1.5 µg/mL, 2.0 µg/mL; Helena Laboratories, Beaumont, TX). These levels were established from fitting a population model to results from period 1 of the previous study so as to optimize estimation of EC_50_ values, and were subsequently validated using pilot data. In addition, a positive and negative control (10 µg/mL and 0.0 µg/mL, respectively) were performed on PRP.

Blood samples (approximately 18 mL) were drawn into 0.32% weight/volume (w/v) sodium citrate. Platelet-rich plasma was prepared from blood by centrifugation at 200× g for 15 minutes at 20°C. Following removal of PRP, samples were centrifuged at 1700× g for 10 minutes to obtain PPP. Platelet-rich plasma samples were adjusted to 200× 10^9^/L with autologous PPP. Light transmittance measurement of platelet aggregation was obtained in Aggregation Remote Analyser Module System (AggRAM) platelet aggregometers (Model 8JF52001, Helena Laboratories, Beaumont, TX) using 100% PRP as a reference for 100% platelet aggregation and 100% PPP as a reference for 0% platelet aggregation. Platelet aggregation assays were performed within 3 hours of blood collection; only fasted, nonlipemic, nonhemolyzed plasma was used. Aggregation studies were performed at the study center; samples were prepared and shipped to Quest Diagnostics (Van Nuys, CA) for measuring Lp-PLA_2_ activity. Lp-PLA_2_ activity was measured in parallel using [^3^H]-PAF as the substrate as previously described [21]. The Lp-PLA_2_ activity is expressed as nmol/min/mL of the PAF consumed or as nmol/min/mL of acetate produced [21].

#### Statistical Methods

An E_max_ model was applied to the collagen log-concentration versus platelet aggregation data to characterize the concentration response relationship. An initial step in the analysis was to obtain common estimated values for slope γ, minimum effect (E_min_), and maximum effect (E_max_) for the study population using data collected at baseline time points. Following log_e_ transformation of the collagen concentration, platelet aggregation data (in % scale based on optimal aggregometry at baseline [0, 6, and 24 hours]) were used to fit a nonlinear mixed model using the maximum likelihood approach. The estimated slope γ, E_min_, and E_max_ were then used as fixed values for all further modelling. From the model, the collagen log-concentration that corresponded with EC_50_ could be derived for each subject at each baseline and post-dose time point (ie, hour 6 on day 1; hour 0 and hour 6 on day 14 and day 35).

The primary end point for this study was the difference between treatment groups in EC_50_ values 21 days after the last study dose (ie, day 35). A leftward shift, indicating a decrease in the collagen concentration versus the aggregation curve, would be indicative of enhanced platelet aggregation.

Analysis of covariance (ANCOVA) models were used to assess the treatment effect at all 3 days (day 1, day 14, and day 35), using the mean EC_50_ across predose and 6-hour observations as outcome and adjusting for the baseline mean EC_50_ (average of EC_50_ values from 3 replicate baseline time points). A separate analysis was carried out for each time point (predose or 6 hours) and was similarly adjusted for baseline mean EC_50_.

The clinically relevant equivalence criterion was originally established at 15%; however, collagen EC_50_ values provided from a pilot study of 4 repeat assessments over a 2-month duration revealed fluctuations from baseline of approximately 23%, without any treatment administration. Based on these data, a reduction in EC_50_ of less than 25% was considered a conservative threshold for demonstrating a lack of enhanced aggregation, and the protocol was amended accordingly prior to study dosing.

The parallel-group study was powered to demonstrate a lack of effect of 14 days of repeat oral doses of 250 mg of rilapladib in platelet aggregation using an equivalence margin of 25% (ie, the lower bound of 95% CIs for the ratio of active drug∶placebo in EC_50_ on day 35>75%). This was evaluated using a noninferiority testing framework based on a clinically specified noninferiority boundary of 75%. A sample size of 40 (∼20 subjects per treatment group) would provide at least 90% power to demonstrate lack of effect, assuming a standard deviation of 0.27, modeled based on the data collected in a previous study. The study was organized in up to 3 cohorts of 28 subjects, with the option to halt the study after one or 2 cohorts based on the observed level of variability in the first cohort assessed in an unblinded fashion.

## Results

### In Vitro Platelet Aggregation Study

Mean (± standard error [SE]) Lp-PLA_2_ activity among the samples treated with vehicle and rilapladib was 27.0 (±2.5) nmol/min/mL and 0.69 (±0.14) nmol/min/mL, respectively, representing a 98% reduction in enzyme activity (vs vehicle) in the rilapladib samples.

**Figure 2:**
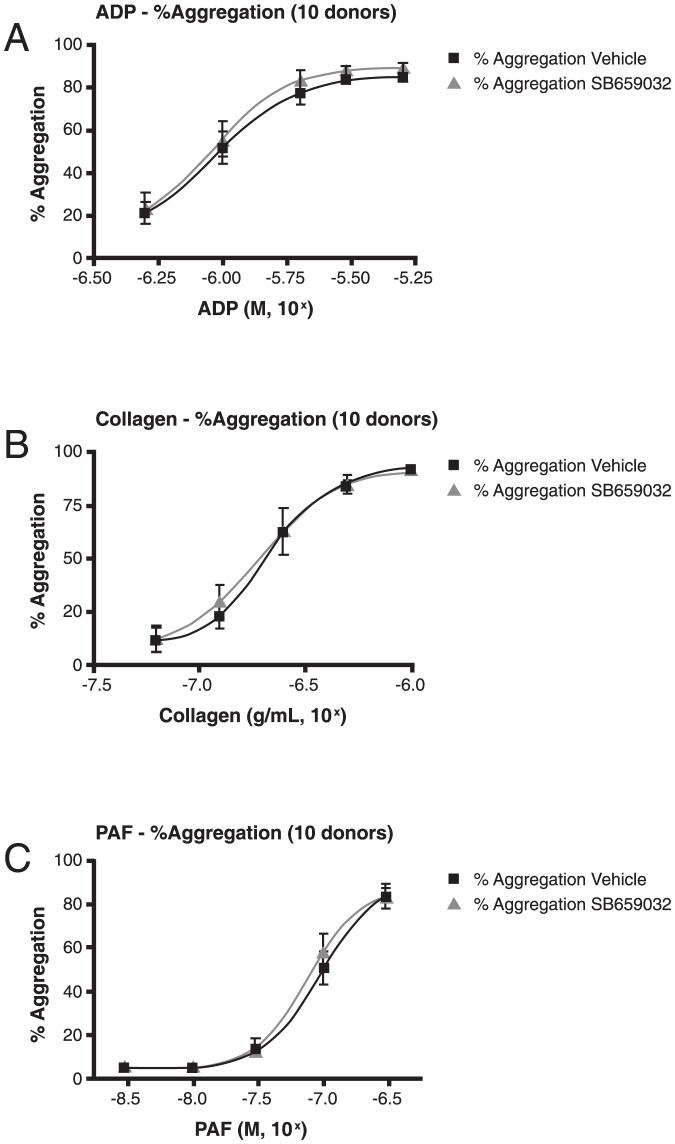
In vitro platelet aggregation results of (A) ADP, (B) collagen, and (C) PAF. ADP, adenosine diphosphate; PAF, platelet-activating factor.

In this study, there was no demonstrable effect of rilapladib on platelet aggregation (vs vehicle) when ADP, collagen, or PAF was used as an agonist (Figure 2, panels A, B, and C, respectively). Fifty-percent maximal platelet aggregation (EC_50_) values were not significantly different between vehicle and rilapladib treatment for each agonist (ADP: 1.08 µM [vehicle] vs 1.05 µM [rilapladib], *p* = .46; collagen: 0.25 µg/mL [vehicle] vs 0.25 µg/mL [rilapladib], *p* = .90; PAF: 129.7 nM [vehicle] vs 111.6 nM [rilapladib], *p* = .24).

### Ex Vivo Platelet Aggregation Study 1: Single agonist-dose assay

Twenty-six healthy adult men were enrolled and 21 completed the study; demographics and clinical characteristics are shown in Table 1. One subject who received rilapladib was withdrawn for an aggregation response greater than 40% with each agonist dose. Withdrawal reasons among those receiving placebo included abnormal liver function tests (n = 1), aggregation responses greater than 40% (n = 2), and microscopic urine red blood cells at baseline for session 2 (n = 1). All subjects (n = 26) were included in the safety analyses and a total of 23 subjects who provided post-dose platelet aggregation data were included in the pharmacodynamic/biomarker analysis.

**Table 1:** Demographics of the study participants in the crossover and E_max_ studies.

Parameter	Crossover study	E_max_ study	
	Total (N = 26)	Rilapladib (n = 28)	Placebo (n = 30)
Age, mean (SD), years	29.8 (8.9)	26.1 (7.5)	28.5 (8.6)
Race, n (%)			
White/Caucasian/European	22 (85)	25 (89)	29 (97)
White/Arabic/North African	1 (4)		
African American/African Heritage	1 (4)	1 (4)	
American Indian or Alaskan Native	1 (4)		
Asian/South East Asian Heritage	1 (4)		
Ethnicity, n (%)			
Hispanic/Latino	1 (4)	1 (4)	1 (3)
Other	25 (96)	27 (96)	29 (97)
Body weight, mean (SD), kg	77.8 (10.2)	NA	NA
Body mass index, mean (SD), kg/m^2^	NA	24 (2.7)	25 (2.4)

SD, standard deviation; NA, not available.

There was no statistically significant effect on platelet aggregation in response to collagen or ADP for rilapladib compared with placebo during 14 days of repeated dosing (Table 2). Treatment with rilapladib demonstrated substantial drug compliance and enzyme inhibition with reduced plasma Lp-PLA_2_ activity by 93.8% and 84.5% at 6 and 24 hours post dose on day 1, respectively, and by 96.6% and 93.4% at these same time points on day 14, respectively.

**Table 2:** Platelet aggregation during the crossover study (Study 1).

Agonist	Day	Time	Aggregation estimate^a^	90% CI
ADP	1	Predose	−4.1	(−7.5, −0.8)
		+6 hours	−0.4	(−3.8, 3.0)
		24 hours	−0.2	(−3.7, 3.2)
	14	Predose	1.8	(−1.6, 5.3)
		+6 hours	−2.5	(−6.0, 0.9)
		24 hours	1.5	(−2.1, 4.9)
	35	Predose	−0.7	(−4.1, 2.8)
		+6 hours	2.7	(−0.7, 6.1)
		24 hours	3.5	(0.0, 6.9)
Collagen	1	Predose	−0.2	(−11.5, 11.2)
		+6 hours	4.7	(−6.7, 16.1)
		24 hours	3.2	(−8.2, 14.6)
	14	Predose	8.3	(−3.3, 19.8)
		+6 hours	3.0	(−8.6, 14.5)
		24 hours	−0.9	(−12.5, 10.7)
	35	Predose	12.1	(0.6, 23.7)
		+6 hours	12.8	(1.2, 24.3)
		24 hours	15.4	(3.9, 27.0)

a The aggregation estimate is the adjusted mean difference between groups (rilapladib minus placebo).

CI, confidence interval; ADP, adenosine diphosphate.

In addition, there were no statistically significant effects on platelet aggregation in response to ADP at all time points at the end of the 21-day washout phase (day 35). All confidence intervals for the treatment differences (active-placebo) with ADP as the agonist were less than 15%. However, at day 35, 21 days off the study treatment, an enhanced platelet aggregation was observed (ie, upper bound of the 90% CI >15% and lower bound >0%) for all 3 time points when collagen was used as the agonist (Table 2). At this point in the study, there were no detectable plasma concentrations of rilapladib or its major metabolite (SB664601), and there was a modest 16.2% mean reduction in Lp-PLA_2_ activity following rilapladib treatment, compared with a 9.4% reduction in activity following placebo, suggesting some possible minimal residual inhibition with active treatment.

#### Within-subject and Between-subject Variability

Based on the concentration-selection criterion (highest concentration at baseline that resulted in aggregation <40%), 9 subjects (43% of the 21 who completed the study) had a different collagen concentration used in period 1 than the one that was used in period 2. Five of these subjects received rilapladib (2 had a higher concentration and 3 had a lower concentration) and 4 received placebo (all 4 had a higher concentration) during the first study phase. Inherent in this approach are 2 different collagen agonist concentrations at the same time; start of period 2 and follow-up of period 1.

Variability between subjects (within collagen dose) was substantial at baseline for both study periods. Mean (± standard deviation [SD]) values (ie, coefficient of variation) for platelet aggregation at baseline of each period is presented in Table S1 in File S1. No significant safety concerns were raised during the study; repeat dosing with rilapladib was generally well-tolerated.

### Ex Vivo Platelet Aggregation Study 2: Emax model

Fifty-eight subjects were enrolled in this study (n = 28 in the rilapladib group and n = 30 in the placebo group), and 56 completed the study (n = 28 in each group). Two subjects assigned to treatment with placebo were withdrawn because of EC_50_ values at baseline that were considered to be out of range (defined as those >1.5 times the interquartile range [quartile 3 – quartile 1] lower than the first quartile or >1.5 times the interquartile range higher than the third quartile). Demographics of the study population are shown in Table 1.

In the rilapladib-treated group, Lp-PLA_2_ was inhibited by 89.9% 6 hours following the first dose (day 1), and by 92.4% and 96.1% before dose and 6 hours post dose, respectively, on day 14 (see Table S2 in File S1). In all cases, differences in percent inhibition between drug and placebo groups were highly significant. Three weeks after the final dose (day 35), Lp-PLA_2_ inhibition by rilapladib (21.7%–23.8%) appeared to persist, which was significantly different from that in the placebo group (6.5%–9.6%). On average, a 13% to 19% increase in EC_50_ over 6 hours from am to pm, consistent with a reduction in collagen induced aggregation, was observed for all time points (eg, in the placebo group at day 35: EC_50_ 0.79 vs 0.91 µg/mL; ratio [6-hour/baseline] 1.16; 95% CI, 1.04–1.29), consistent with previous reports of circadian rhythms [23].

**Figure 3:**
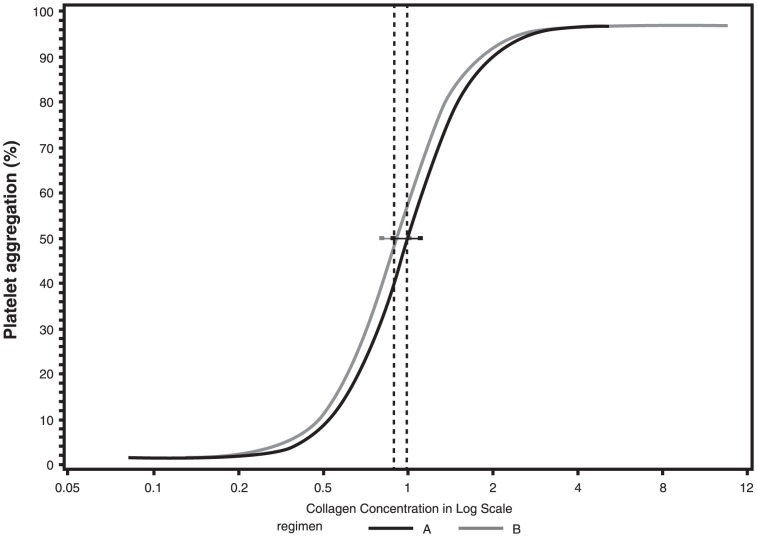
Concentration response curves for collagen-induced platelet aggregation 14 days post-dosing (treatment A = rilapladib, treatment B = placebo).

There was no statistically significant effect on platelet aggregation in response to collagen (compared with placebo) during rilapladib dosing or during the off-drug period at all time points (Table 3, Figure 3). Specifically, on day 14 the EC_50_ ratio (active∶placebo) was 1.10 (95% CI, 0.98–1.23), while on day 35, the ratio of EC_50_ for rilapladib to placebo was 1.06 (95% CI, 0.94–1.20). All point estimates in EC_50_ ratios (active∶placebo) for all the time points were greater than 1, suggesting on average no apparent aggregation effect of concern. Any decrease in EC_50_ value would be a concerning trend. Comparable results were observed on the other study days and time points (Table 3). Thus, treatment with rilapladib did not enhance collagen-induced platelet aggregation, as demonstrated by both the slight increase in average EC_50_ values for rilapladib compared with placebo and the lower bound of the 95% CI for the ratio of collagen EC_50_ for rilapladib-treated subjects relative to placebo-treated subjects greater than 0.75 (ie, ruling out a 25% reduction in EC_50_).

**Table 3:** Comparison of the EC_50_ for collagen-induced platelet aggregation (Study 2).

Day	Time	EC_50_ rilapladib	EC_50_ placebo	Ratio^a^	95% CI
1	+6 hours	1.31	1.23	1.07	(0.92, 1.23)
14	Pre	0.87	0.81	1.07	(0.92,1.24)
	+6 hours	1.13	1.00	1.12	(0.92, 1.38)
	Average	0.99	0.90	1.10	(0.94, 1.29)
35	Pre^b^	0.85	0.82	1.04	(0.94, 1.16)
	+6 hours^c^	1.10	1.02	1.08	(0.91, 1.28)
	Average	0.97	0.91	1.06	(0.94, 1.20)

a EC_50_ rilapladib/EC_50_ placebo.

b Corresponding to the predose time during treatment.

c Corresponding to the 6 hours postdose time during treatment.

EC_50_, 50% maximal platelet aggregation; CI, confidence interval.

## Discussion

The results of these studies demonstrate that near-complete Lp-PLA_2_ inhibition with rilapladib) resulted in no demonstrable effect on platelet aggregation in healthy subjects. Although Lp-PLA_2_ can hydrolyze PAF in vitro, and appreciating that other metrics of platelet activity were not tested, these data suggest that inhibiting this enzyme in vivo offers at best limited alteration in PAF-mediated biology, measured in this instance by platelet aggregation.

Given that prior addition of rilapladib to human PRP, resulting in complete inhibition of Lp-PLA_2_, did not influence the concentration response curve of PAF-stimulated human platelet aggregation, we began to corroborate these compelling in-vitro findings in human subjects. In an initial, uncontrolled, single-dose pilot study with rilapladib involving 14 healthy adult volunteers (unpublished data not shown here), a proaggregatory signal (vs baseline) was observed 6 hours post dose and after a 2-week follow-up period, prompting additional studies to explore the potential proaggregatory effects of Lp-PLA_2_ inhibition in a more rigorous manner. Using more robust methods, the additional studies described herein consistently demonstrate a lack of effect of Lp-PLA_2_ inhibition on platelet aggregation. In the crossover study described in this report, which used the sub-maximum concentration chosen among 3 fixed levels using a priori rule (<40%), an overall lack of effect on platelet aggregation with rilapladib was observed. However, the nominal signal with collagen 21 days after the last study dose, as well as the variability inherent in single-dose assays, led to the design and conduct of the more definitive E_max_ method. The E_max_ method offers the greatest precision by characterizing the complete aggregation dose response for each study participant at each time point to compare mean EC_50_ values for the aggregation responses between treatment groups. These data were more reproducible over time, and did not depend on the selection of a correct sub-threshold agonist concentration. Demonstrating less than a 25% increase in aggregation, the smallest detectable difference outside of assay error and a range considered to have no impact on coagulation, diminishes the chance of a clinically relevant platelet-induced adverse effect from rilapladib or darapladib therapies. Further, the absence of any trend toward aggregation at every time point in vitro and in vivo from our clinical data does not suggest a critical role of Lp-PLA_2_ inhibition in enhancing PAF-mediated biology when applied to agonist-induced platelet aggregation.

Despite these compelling clinical studies using a direct and extremely potent inhibitor of the Lp-PLA_2_ enzyme, these findings contrast with results of several previously published reports that suggest Lp-PLA_2_ (plasma PAF-AH) activity is inversely associated with disorders in which PAF has been implicated, including asthma (related to overall risk [13], [17] or severity [14], [16]) and anaphylaxis [18]. These studies were largely conducted in the Japanese population, in which a common Lp-PLA_2_ gene variant (Val279Phe) is associated with reduced (heterozygotes; 27% of the population) or complete lack (homozygotes; 4% of the population) [14], [24] of plasma Lp-PLA_2_ activity. However, similar findings on associations between Lp-PLA_2_ and asthma risk have also been reported with other gene variants in non-Japanese populations [25], [26]. Consistent findings have not been observed across all studies with the Val279Phe variant. In one study involving individuals homozygous or heterozygous for the Val279Phe gene variant, plasma Lp-PLA_2_ activity was not associated with asthma prevalence, type (atopic vs nonatopic), or severity [15].

Aside from the studies described herein, relatively little “direct” experimental evidence is available describing the effects of PAF inhalation-mediated responses. In a randomized, double-blind, placebo-controlled crossover trial [11], 14 subjects with mild atopic asthma received either recombinant human PAF-AH (ie, Lp-PLA_2_; target plasma concentration of >10 µg/mL) or placebo followed by exposure (inhaled) to subject-specific allergen (based on skin prick testing). In this study, treatment with recombinant PAF-AH was not associated with statistically significant differences (vs placebo) in pulmonary function or in early or late asthmatic responses following bronchial allergen challenge [11]. Another clinical study evaluated the effects of inhaled PAF on pulmonary function and inflammatory cell kinetics in 8 healthy individuals homozygous for the Val279Phe variant and 16 age-matched and sex-matched controls (6 who were heterozygous for the variant and 10 with the wild-type allele) [12]. While these subjects were not asthmatic, there were no statistically significant differences in the percent decrease in FEV_1_ following PAF inhalation among the 3 Lp-PLA_2_ genotypes. Specifically, a decrease in FEV_1_ of greater than 10% was observed in 3 subjects (38%) with PAF-AH deficiency and in 5 subjects (31%) from the control group. Transient neutropenia following PAF inhalation also did not differ among genotypes in magnitude or duration [12]. This latter, more direct, study suggests that the impact of administered PAF is little influenced by the level of LpPLA_2_ activity.

Two interpretations of the findings presented herein are (1) PAF is not a predominant factor in the underlying inflammatory processes in platelet aggregation, or (2) PAF-AH (Lp-PLA_2_) is not a principal mechanism involved in inactivating PAF in vivo. The possibility that Lp-PLA_2_ may not be principally involved in the hydrolysis and inactivation of PAF in vivo is further supported by the enzyme kinetics. In particular, the Michaelis constant (Km) of Lp-PLA_2_ for PAF is reported to be ∼14 µM; however, PAF elicits biologic responses at nanomolar concentrations [27], [28], which is consistent with findings in the present in vitro platelet aggregation study. Furthermore, it has been reported that the half-life for PAF in plasma or whole blood (both of which contain secreted Lp-PLA_2_) from otherwise healthy individuals is approximately 5 minutes [29]. These enzyme characteristics tend to question the role of Lp-PLA_2_ in hydrolyzing and inactivating physiologic relevant concentrations of PAF in vivo since this phospholipid mediator is biologically active in the low nM range, having a calculated dissociation constant (Kd) for its receptor of around 1 nM [30], a concentration of PAF that is more in line to what is found circulating in humans [18]. These data are consistent with a recent report by Liu et al, which suggests that rapid clearance of PAF by endothelial cell rich organs, such as the liver, represents the major route of in vivo PAF catabolism rather than Lp-PLA_2_ activity when assessed in Lp-PLA_2_–deficient mice. [31].

In a previous 12-week clinical trial of coronary heart disease (CHD) or CHD-risk equivalent subjects (n = 959), administration of darapladib, a less potent Lp-PLA_2_ inhibitor than rilapladib, did not induce a change in biomarkers related to platelet activity (P-selectin, CD40 ligand, or urinary 11-dehydrothromboxane B2) [32]. These markers, which might be suspected to be up-regulated in the setting of increased PAF influence, do not appear altered by LpPLA2 inhibition, consistent with our findings. In this study, darapladib achieved an approximately 80% inhibition of Lp-PLA_2_ using the same radiometric assay used in our two current studies with rilapladib, which demonstrated Lp-PLA_2_ inhibition of ∼90% [32]. Therefore, in the clinical studies presented herein, as well as in the biomarker study, there was at best a vanishingly small level of Lp-PLA_2_ activity available for influencing PAF catabolism, particularly when considering physiologically relevant low nM levels of PAF. In addition, one might argue that the more potent effects of rilapladib make this agent the more compelling approach to test the potential effect of residual PAF on biologic functions such as platelet aggregation, as we have done in the studies described in this report.

A profoundly hypercoagulable state (in contrast to the cohorts of our studies) could result in a proclivity toward platelet aggregation not observed in healthy controls. Such a concern is perhaps offset by the experimental design of full collagen-induced aggregation used in the current study. Further, although only one platelet agonist (collagen) was used in the E_max_ model, collagen operates through multiple platelet pathways, thereby representing a fairly robust platelet aggregation signal. In addition, despite observing the anticipated contrasts in platelet aggregation throughout the day (circadian variation), ex vivo studies did not include a positive control for potentiation of platelet aggregation. Our studies were exclusively performed in men, and thus although it is not clear that our findings can be extended to women, it would be reasonable to expect similar findings in women. Finally, while platelet aggregation incorporates many elements of platelet function and activity, there are other metrics of platelet activity and/or biology that we did not test.

In conclusion, these data suggest that near-complete Lp-PLA_2_ (PAF-AH) inhibition did not substantially influence platelet aggregation preclinically in vitro or clinically ex vivo in male subjects. These data support the notion that PAF hydrolysis does not factor critically into the physiologic phenotype of platelet thrombogenicity that is, for example, associated with myocardial infarction and other thrombotic disorders. Further studies are necessary to conclusively determine the influence of Lp-PLA_2_ on PAF-mediated biology.

## Supporting Information

File S1 **Table S1, Mean ± standard deviation for platelet aggregation at baseline of each period in crossover study. Table S2, Comparison of percent plasma Lp-PLA_2_ Inhibition in the ex vivo platelet study.**
(DOC)

Checklist S1 **Completed CONSORT checklist.**
(DOC)

Protocol S1 **Protocols for each of the reported studies.**
(PDF)
