# Supplementary material for: Platelet Aggregation Unchanged by Lipoprotein-Associated Phospholipase A2 Inhibition: Results from an In Vitro Study and Two Randomized Phase I Trials
Source: PLoS One. 2014 Jan 27;9(1):e83094. doi: 10.1371/journal.pone.0083094 (PMC3903475; doi:10.1371/journal.pone.0083094)
Supplement: File S1 — Table S1, Mean ± standard deviation for platelet aggregation at baseline of each period in crossover study. Table S2, Comparison of percent plasma Lp-PLA2 Inhibition in the ex vivo platelet study. (DOC) [file pone.0083094.s001.doc]

**SUPPORTING TEXT**

**Table S1.** Mean ± standard deviation for platelet aggregation at baseline of each period in crossover study

|  | Period 1 | Period 2 |
| --- | --- | --- |
| Collagen = 0.7 μg/mL | 8.2 ± 7.1 | 11.8 ± 17.1 |
| Collagen = 1.0 μg/mL | 32.1 ± 32.3 | 25.5 ± 28.5 |
| Collagen = 1.2 μg/mL | 47.2 ± 33.5 | 41.8 ± 35.2 |

**Table S2.** Comparison of percent plasma Lp-PLA_2_ Inhibition in the ex vivo platelet study

| Day | Time | % inhibition rilapladib | % inhibition placebo | Difference^a^ | 95% CI |
| --- | --- | --- | --- | --- | --- |
| 1 | + 6 hr | 89.88 | 0.93 | 88.95 | (78.18-99.72) |
| 14 | Pre | 92.43 | 10.44 | 81.98 | (78.08-85.89) |
|  | + 6 hr | 96.10 | 9.45 | 86.65 | (82.75-90.55) |
| 35 | Pre^b^ | 23.82 | 9.60 | 14.22 | (8.15-20.30) |
|  | +6 hr^c^ | 21.65 | 6.50 | 15.15 | (9.35-20.95) |
|  |  |  |  |  |  |

^a^The difference in % inhibition was calculated as the least square mean value in the rilapladib-treated group minus the least square mean value in the placebo-treated group.

^b^Corresponding to the predose time during treatment.

^c^Corresponding to the 6 hours postdose time during treatment.

Lp-PLA_2_, lipoprotein-associated phospholipase A_2_
