# Supplementary material for: Platelet Aggregation Unchanged by Lipoprotein-Associated Phospholipase A2 Inhibition: Results from an In Vitro Study and Two Randomized Phase I Trials
Source: PLoS One. 2014 Jan 27;9(1):e83094. doi: 10.1371/journal.pone.0083094 (PMC3903475; doi:10.1371/journal.pone.0083094)
Supplement: Protocol S1 — Protocols for each of the reported studies. (PDF) [file pone.0083094.s003.pdf]

Division: Worldwide Development
Retention Category: GRS019
Information Type: Protocol

**Title:**	A Double Blind Study to Evaluate Effects of Repeat Doses of SB-659032 on Platelet Aggregation in Healthy Male Volunteers

Compound Number: SB-659032

Effective Date: 07-Jun-2005

Description:

SB-659032 is a selective and orally active inhibitor of lipoprotein-associated phospholipase A_2 (Lp-PLA_2) that is being developed for the treatment of atherosclerosis. This study is designed to assess whether inhibition of plasma Lp-PLA_2 activity impacts platelet function as assessed by *ex vivo* platelet aggregation tests and *in vivo* plasma biomarkers. Blood samples for PK analysis and measurement of Lp-PLA_2 activity will also be collected. Questionnaires will be completed to evaluate the frequency of odor-related AEs with non-enteric coated formulation of SB-659032 relative to placebo. This will be a double blind, repeat dose, randomized, placebo-controlled, two period, period balanced, crossover study. There will be a minimum of a 21 day washout period between dosing in each period.

Subject: SB-659032, Platelet Aggregation

Author(s):

Yun, Suyong CSSO, CPDM
Magee, Mindy CPK/M&S, CPDM
Sethuraman, Venkat S CPSP, BDS
Morgan, John M DM, CPDM

Copyright 2005 the GlaxoSmithKline group of companies. All rights reserved.
Unauthorised copying or use of this information is prohibited.

CONFIDENTIAL ZM2005/00032/00
LP2104623

Sponsor Signatory:

A Johnson — June 7, 2005

Tony Johnson, MD
VP, Cardiovascular/ Urogenital Discovery Medicine

Date

SPONSOR INFORMATION PAGE

Clinical Study Identifier: LP2104623

GlaxoSmithKline
2301 Renaissance Boulevard
Building #510
Post Office Box 61540
King of Prussia, Pennsylvania 19406
Telephone number: +1 610 787-7000

Medical Monitor Contact Information:

John M. Morgan, M.D.	
Telephone Number	610 270 4593
After hours Contact Number	215 495 2332
Fax Number	610 270 4114

Back-up Medical Monitor Information:

Pedro C. Fernandez, M.D.	
Telephone Number	610 270 6217
After hours Contact Number	215 833 7692
Fax Number	610 270 5250

Regulatory Agency Identifying Number(s): 66,502

INVESTIGATOR PROTOCOL AGREEMENT PAGE

I confirm agreement to conduct the study in compliance with the protocol.

Investigator Name: ______________________________

______________________________ ______________________
Investigator Signature Date

Table of Contents

	Page
ABBREVIATIONS	8
PROTOCOL SUMMARY	10
1. INTRODUCTION	14
1.1. Background	14
1.2. Preclinical Studies	15
1.3. Clinical Studies	16
1.4. Rationale	27
2. OBJECTIVE(S)	28
2.1. Primary	28
2.2. Secondary	28
3. ENDPOINT(S)	29
3.1. Primary	29
3.2. Secondary	29
4. STUDY DESIGN	29
5. STUDY POPULATION	29
5.1. Number of Subjects	29
5.2. Eligibility Criteria	30
5.2.1. Inclusion Criteria	30
5.2.2. Exclusion Criteria	30
5.2.3. Other Eligibility Criteria Considerations	31
6. STUDY ASSESSMENTS AND PROCEDURES	31
6.1. Demographic and Baseline Assessments	32
6.1.1. Screening	32
6.1.2. Day -1 and Predose	32
6.2. Dosing/Post-Dose Procedures	33
6.3. Follow-up Visit	34
6.4. Safety	34
6.4.1. 12-Lead ECG Monitoring	34
6.4.2. Vital Sign and Clinical Laboratory Assessment	34
6.4.3. Pregnancy	35
6.5. Pharmacokinetics	35
6.5.1. Sample Collection and Preparation	35
6.5.2. Assay Methodology	35
6.5.3. Planned Pharmacokinetic Evaluation	35
6.6. Pharmacodynamics	36

6.6.1. Sample Collection and Preparation 36
6.6.2. Assay Methodology 36
6.6.3. Planned Pharmacodynamic Evaluation 37
6.7. Biomarker(s) 37
6.7.1. Sample Collection and Preparation 37
6.7.2. Assay Methodology 38
6.7.3. Planned Biomarker Evaluation 38
6.8. Odor Assessments 38
6.9. Pharmacogenetics 38

7. LIFESTYLE AND/OR DIETARY RESTRICTIONS 38
7.1. Meals and Dietary Restrictions 38
7.2. Contraception 40
7.3. Other Restrictions 40

8. INVESTIGATIONAL PRODUCT(S) 40
8.1. Description of Investigational Product 40
8.2. Dosage and Administration 40
8.3. Dose Rationale 40
8.4. Blinding 41
8.5. Treatment Assignment 42
8.6. Packaging and Labeling 42
8.7. Preparation 42
8.8. Handling and Storage 42
8.9. Product Accountability 42
8.10. Assessment of Compliance 42
8.11. Treatment of Investigational Product Overdose 43
8.12. Occupational Safety 43

9. CONCOMITANT MEDICATIONS AND NON-DRUG THERAPIES 43
9.1. Permitted Medications 43
9.2. Prohibited Medications 43

10. SUBJECT COMPLETION AND WITHDRAWAL 43
10.1. Subject Completion 43
10.2. Subject Withdrawal 44
10.2.1. Subject Withdrawal from Study 44
10.2.2. Subject Withdrawal from Investigational Product 44
10.3. Treatment After the End of the Study 44

11. ADVERSE EVENTS (AE) AND SERIOUS ADVERSE EVENTS (SAE) 44
11.1. Definition of an AE 44
11.2. Definition of a SAE 45

11.2.1. Clinical Laboratory Abnormalities and Other Abnormal Assessments as AEs and SAEs 45
11.3. Time Period, and Frequency of Detecting AEs and SAEs 46
11.4. Prompt Reporting of SAEs to GSK 46
11.4.1. Timeframes for Submitting SAE Reports to GSK 47
11.5. AE and SAE Documentation and Follow-up Procedures 47
12. DATA ANALYSIS AND STATISTICAL CONSIDERATIONS 47
12.1. Hypotheses 47
12.2. Study Design Considerations 47
12.2.1. Sample Size Assumptions 47
12.2.2. Sample Size Sensitivity 48
12.2.3. Sample Size Re-estimation 48
12.3. Data Analysis Considerations 48
12.3.1. Analysis Populations 48
12.3.2. Analysis Data Sets 48
12.3.3. Treatment Comparisons 49
12.3.4. Interim Analysis 49
12.3.5. Key Elements of Analysis Plan 49
13. STUDY CONDUCT CONSIDERATIONS 51
13.1. Regulatory and Ethical Considerations, Including the Informed Consent Process 51
13.2. Quality Control (Study Monitoring) 51
13.3. Quality Assurance 52
13.4. Study and Site Closure 52
13.5. Records Retention 52
13.6. Provision of Study Results and Information to Investigators 53
13.7. Data Management 53
13.8. Independent Data Monitoring Committee (IDMC) 53
14. REFERENCES 54
Appendices 55
Appendix 1: Time and Events Table 55
Appendix 2: Clinical Laboratory Tests 58
Appendix 3 : Values of potential Clinical Concerns 59
Appendix 4: PGx 60

ABBREVIATIONS

2,3-diFBCl	2,3-difluorobenzylchloride
ADP	Adenosine Diphosphate
AE	Adverse Event
ANOVA	Analysis of Variance
AUC	Area under the plasma concentration vs time curve
ALT	Alanine amino transferase
AST	Aspartate amino transferase
bTG	β-thromboglobulin
BUN	Blood Urea Nitrogen
CI	confidence interval
CBC	Complete blood count
CIB/IB	Clinical Investigator's Brochure/Investigator's Brochure
CPK	Creatinine phosphokinase
Cmax	Maximum drug concentration
Cmin	Minimum drug concentration
CRF	Case report form (electronic or hard copy)
CSR	Clinical study report
CV	Cardiovascular
CYP3A4	cytochrome P450 enzyme 3A4
DNA	deoxyribonucleic acid
EC	enteric coated
ECG	Electrocardiogram
FDA	Food and Drug Administration
GCP	Good Clinical Practices
GGT	Gamma glutamyltransferase
GSK	GlaxoSmithKline
HIV	Human Immunodeficiency Virus
IEC	Independent Ethics Committee
IND	Investigational New Drug application
IRB	Institutional Review Board
IUD	Intrauterine device
LDL	Low density lipoprotein
LLQ	Lower limit of quantification
Lp-PLA_2	Lipoprotein-associated phospholipase A_2
Lyso-PC	Lysophosphatidylcholine
M	Molar
MSDS	Material Safety Data Sheet
NOAEL	No Adverse Effect Level
PD	Pharmacodynamic
PAF-AH	platelet activating factor acetylhydrolase
PLA_2	Phospholipase A_2
PGx	Pharmacogenetics
PK	Pharmacokinetic
ppm	Parts per million
PRP	Platelet rich plasma
QC	Quality Control

RAP	Report and Analysis Plan
RBC	Red blood cell
SAE	Serious adverse event
sCD40L	Soluble CD40 ligand
sd	Standard deviation
SNP	Single Nucleotide Polymorphism
SRM	Study Reference Manual
TxB2	Thromboxane B2
T½	Terminal phase elimination half-life
Tmax	Time of maximal plasma concentration
UDS	Urine drug screen
ULN	Upper limit of normal

Trademarks

GSK Trademarks

Trademarks not owned by GlaxoSmithKline
Plavix
Heparin

PROTOCOL SUMMARY

Rationale

SB-659032 is a selective and orally active inhibitor of lipoprotein-associated phospholipase A_2 [Lp-PLA_2] that is being developed for the treatment of atherosclerosis. Lp-PLA_2, also known as platelet activating factor acetylhydrolase (PAF-AH), is one of the enzymes responsible for the hydrolysis of PAF [platelet activating factor] into biologically inactive lyso-PAF. PAF is synthesized by a variety of cell types including platelets, neutrophils, monocytes, mast cells and eosinophils. In platelets, PAF promotes aggregation by stimulating a G protein-linked cell surface receptor. However, it does not function as an independent mediator of aggregation [Morrow, 2001].

SB-659032 will be studied in patients with documented evidence of coronary heart disease including patients with acute coronary syndrome where the use of antiplatelet agents such as aspirin and/or clopidogrel is considered part of standard care. It is theoretically possible that an Lp-PLA_2 inhibitor may result in accumulation of PAF, promoting platelet aggregation and hence negate the beneficial effects of anti-platelet agents. In a clinical study (study LP2102487) in which healthy subjects were administered a single 250 mg dose of the compound SB-659032, preliminary data [data on file] suggests that 11 out of 14 subjects had enhanced platelet aggregation (>15% change from baseline) at 6 hours post-dose following the addition of 0.1 or 0.2 mcg/mL of the agonist collagen. In addition, 3 out of the 14 subjects displayed enhanced platelet aggregation at 24 hours post-dose and 8 out of the 14 subjects displayed enhanced platelet aggregation at the 14 day follow-up visit. In the absence of a placebo control in this trial, the clinical significance of these results is unclear, highlighting the need to complete a placebo-controlled study. Therefore, this study is designed to assess the effect of SB-659032 on platelet function compared to placebo when administered daily for 14 days.

Non-enteric coated, 250 mg repeat doses of SB-659032 will be utilized in this study. This dose will be administered with a low-fat breakfast in an attempt to minimize generation of 2,3-difluorobenzylchloride in the stomach. *Ex vivo* platelet aggregation tests using ADP and collagen as agonists will be performed at pre-dose, 6 hours post-dose, 24 hours post-dose on Day -1, 1 and 14, and at the 21 day (minimum) post-last dose. Considering the mode of action of aspirin and clopidogrel, the use of ADP and collagen as agonists for the purposes of assessing platelet function in the current study are deemed appropriate to ascertain whether an Lp-PLA_2 inhibitor, SB-659032, can negate the effect of these anti-platelet agents. Soluble CD40 ligand (sCD40L) in serum will be measured at the same time points as the platelet aggregation tests on Day 1 and 14 and at the 21 day (minimum) post-last dose. Urinary 11-dehydrothromboxane B2 (U-11-dehydro-TxB2) will be measured from a 24 hour urine collection on Days -1, 1, 14 and at the 21 day (minimum) post-last dose visit.

Objective(s)

Primary

1. To estimate the effect of single and repeat oral doses of 250 mg of non-enteric coated SB-659032 on platelet aggregation as compared to placebo
2. To assess the effect of single and repeat oral doses of 250 mg of non-enteric coated SB-659032 on biomarkers of platelet function as compared to placebo

Secondary

1. To evaluate the degree of inhibition of plasma Lp-PLA_2 activity following single and repeat dosing of non-enteric coated SB-659032
2. To assess the safety and tolerability of a non-enteric coated SB-659032
3. To monitor mean concentrations of SB-659032 and its major metabolite, SB-664601
4. To assess the frequency of odor-related adverse events as reported by subjects following repeated dosing with a non-enteric coated formulation of SB-659032 in healthy subjects

Endpoint(s)

Primary

1. Percent maximum platelet aggregation following ADP- and collagen-induced aggregation
2. Urinary 11-dehydrothromboxane B2 and serum soluble CD40 ligand concentrations

Secondary

1. Plasma Lp-PLA_2 activity, expressed in terms of percent inhibition relative to baseline
2. Clinical safety data from spontaneous adverse event reporting, 12-Lead ECG, vital signs, nursing/physician observation and safety laboratory tests will be summarized and any clinically significant abnormalities will be described
3. Geometric mean concentrations of SB-659032 and its major metabolite, SB-664601, will be summarized in a tabular form and descriptive statistics will be calculated.
4. The frequency of odor-related adverse events as reported by subjects will be summarized in a tabular form and descriptive statistics will be calculated.

Study Design

This will be a double blind, repeat dose, randomized, placebo-controlled, two period, period balanced, crossover study. The entire study will consist of a minimum of 12 overnight stays and 20 outpatient visits. There will be a minimum of a 21 day washout period between dosing in each period.

Regimen	Description
A	250 mg non-enteric coated SB-659032 QD for 14 days
B	SB-659032 matched placebo QD for 14 days

Subjects will return for an assessment, at a minimum of 21 days following the last dose of study medication in each period. This will require an additional 2 night stay, unless these assessments are performed at the same time as the Day -1 procedures for Period 2. The 21 day (minimum) post-last dose visit for Period 2 will also be the last follow-up visit. Each subject's participation in the study will be approximately 13 weeks from screening to follow-up.

Study Population

A sufficient number of subjects will be enrolled so that at least 20 subjects complete the study. Eligible subjects will be healthy adult males between 18 and 55 years of age.

Study Assessments

Subjects will report to the research facility at least 48 hours prior to dosing in each period and will remain in the facility until the morning of Day 2. On the morning of Day 1, subjects will be randomized to receive either 250 mg of SB-659032 or SB-659032 matched-placebo for 14 days. Subjects will be dosed as outpatients on Days 3 to 12. On the evening of Day 12, subjects will be re-admitted to the facility and will remain in house for the remainder of the period. Blood samples for pharmacokinetic analysis will be collected prior to dosing and at 6 and 24 hours following dosing on Days 1 and 14. Blood samples for platelet aggregation will be collected prior to dosing and at 6 and 24 hours following dosing on Day 1 and Day 14. Samples will be taken at the equivalent times on Day -1 and at the 21 day (minimum) post-dose visit. Blood samples for Lp-PLA_2 and sCD40L analysis will be collected prior to dosing and at 6 and 24 hours following dosing on Day 1 and Day 14. In addition, a blood sample will be collected for one assessment at the time of the first platelet aggregation assessment at the 21 day (minimum) post-last dose visit. Urine samples (0-24 hour) will be collected for urinary 11-dehydrothromboxane B2 analysis on Days -1, 1, 14 and at the 21 day (minimum) post-dose visit. The frequency of odor-related AEs will be assessed by subjects with implementation of the questionnaire/scale at the end of each period. Subjects will be asked to return at least 24 hours prior to the 21 day (minimum) post-last dose assessments for a 2 night stay. For both periods and for all subjects, this visit should occur at exactly the same number of days following the last dose of SB-659032. The 21 day post-last dose visit for Period 2 will also be the last follow-up.

Assessment of tolerability to study medication will be made during the study, as described or as necessary, by heart rate and blood pressure measurements, 12-Lead ECG, laboratory safety tests, adverse event questioning and nursing/physician observation.

Subjects participating in this study will be given the option of participating in pharmacogenetic research. If a subject consents to participate, a blood sample will be collected.

1. INTRODUCTION

1.1. Background

Cardiovascular (CV) disease accounts for approximately 50% of all deaths (from myocardial infarction and stroke) in both developed and developing countries. Atherosclerosis is the fundamental pathology behind most CV deaths and is now universally acknowledged as an inflammatory disease and not one that simply results from the arterial accumulation of lipids. This specialized inflammatory disease of the intima is characterized by leukocyte (monocytes and T-lymphocytes) recruitment and accumulation having a major role in both early and continuing aspects of its pathogenesis. Indeed, the histopathological examination of plaques that have been identified as culprit lesions causing a myocardial infarction more often than not occupy less than 50% of the vessel lumen cross sectional area, have a soft lipid core, and a thin friable cap with a high concentration of macrophages in the so called "shoulder region." Thus, macrophage driven chronic inflammation appears to represent an important destabilizing process in the arterial lesion [Ross, 1999].

The sub-endothelial oxidation of low density lipoprotein (LDL) is viewed as a highly significant biological process that both initiates and accelerates arterial lesion development [Ross, 1999]. One of the earliest events in LDL oxidation is the hydrolysis of oxidatively modified phosphatidylcholine, generating substantial quantities of lysophosphatidylcholine (lyso-PC) and oxidized fatty acids. This hydrolysis is mediated by lipoprotein-associated phospholipase A_2 (Lp-PLA_2), an enzyme that is found associated predominantly with LDL in human plasma [Tew, 1996]. A significant volume of evidence has accumulated in favor of both by-products, especially lyso-PC, being proinflammatory and proatherogenic mediators [Macphee, 1999]. Thus, preventing the generation of these mediators through inhibition of Lp-PLA_2 should retard atherosclerosis by interfering with inflammatory cell localization, activation, pro-inflammatory function and death. Consistent with this notion is the recent observation that plasma levels of Lp-PLA_2 represent an independent predictor of coronary heart disease [Packard, 2000].

SB-659032 may prevent the accumulation of lyso-PC and other pro-inflammatory lipids generated during the oxidation of LDL. Lyso-PC has several pro-atherogenic activities ascribed to it, including monocyte chemotaxis and induction of endothelial dysfunction, both of which facilitate monocyte-derived macrophage accumulation within the artery wall. An inhibitor of Lp-PLA_2 such as SB-659032 is predicted to inhibit intimal macrophage accumulation and therefore retard and stabilize the developing atherosclerotic plaque.

SB-659032 represents the third inhibitor of Lp-PLA_2 to be evaluated in the clinic, the first two having been SB-435495 and SB-480848. SB-659032 is a back up compound of SB-480848 that has been in clinical development since 2001.

Lp-PLA_2 is often referred to in the literature as platelet-activating factor acetyl hydrolase (PAF-AH) since it was first characterized as a plasma enzyme activity that hydrolyzed exogenously added PAF. Lp-PLA_2 and PAF-AH are different names for the same enzyme but Lp-PLA_2 is a more general term, because this enzyme can hydrolyze a broad

range of substrates in addition to PAF. Lp-PLA_2 may be a primary mechanism of PAF removal *in vivo* and a deficiency in its enzymatic activity could potentially enhance PAF-mediated responses and exacerbate asthmatic conditions. Hence, patients with asthma are excluded in clinical trials of SB-659032 until an appropriate study in an asthmatic population with an Lp-PLA_2 inhibitor has been performed. In addition, it is theoretically possible that an Lp-PLA2 inhibitor may result in accumulation of PAF, promoting platelet aggregation. Preliminary data from the LP2102487 study suggests that 11 out of 14 subjects had enhanced platelet aggregation (>15% change from baseline) at 6 hours post-dose following the addition of 0.1 or 0.2 mcg/mL of the agonist collagen. In addition, 3 out of the 14 subjects displayed enhanced platelet aggregation at 24 hours post-dose and 8 out of the 14 subjects displayed enhanced platelet aggregation at the 14 day follow-up visit. The study was not placebo control, which limited the ability to interpret the data. Hence, the current study is designed to examine whether repeat, oral, 250 mg doses of SB-659032 have any effect on platelet function as assessed by *ex vivo* platelet aggregation tests (using ADP and collagen as agonists) as well as *in vivo* biomarkers of platelet function [Urinary 11-dehydrothromboxane B2 (U-11-dehydro-TxB2) and soluble CD40 ligand (sCD40L)] as compared to placebo.

SB-659032 absorbs light in the waveband of concern (290-700 nM) for potential phototoxicity, and (based on rat data) shows an association with melanin in skin and eye. Although, the risk associated with this observation is likely to be very low. Subjects should avoid direct sunlight during the study, from the administration of the first dose until 3 days after the last dose. Sunblock (SPF ≥ 30) should be applied to areas of skin exposed to direct sunlight for more than 15 minutes.

Please refer to the Investigator Brochure [GlaxoSmithKline Document Number ZM2004/00059/00] for further information regarding preclinical data.

1.2. Preclinical Studies

The potential effects of SB-659032 on female fertility and embryofetal development have been assessed in rats at doses of up to 100 mg/kg/day. Maternal body weight gain and food consumption were reduced at 100 mg/kg/day but there were no effects on female fertility and no evidence of developmental toxicity (AUC (0-t) >13000 ng.h/mL). In addition, in a study to assess embryofetal development in the rabbit, maternal food consumption was reduced at the highest dose of 50 mg/kg/day, but there was no evidence of developmental toxicity (AUC (0-t) of 6040 ng.h/mL).

SB-659032 was not genotoxic *in vitro* in Ames tests or the mouse lymphoma cell mutation assay, and did not induce chromosomal damage in a micronucleus test in rats. SB-659032 can form 2,3-difluorobenzylchloride (2,3-diFBCl) in the presence of hydrochloric acid. Based on its structure, 2,3-diFBCl is potentially genotoxic. A structurally related *in vitro* acid degradant of SB-480848, 4-fluorobenzyl chloride (4-FBCl), was weakly positive in a single bacterial strain in an Ames test and resulted in a small increase (3- to 6-fold) in mutant colonies in a mouse lymphoma assay. However, further testing *in vivo* showed that 4-FBCl was not genotoxic in a rat micronucleus assay at the maximum tolerated repeat oral dose (250 mg/kg/day for 14 days) using a sensitive target tissue, bone marrow. Forced degradation of SB-659032 in both 0.1M hydrochloric

acid and simulated gastric fluid (containing pepsin and bile salts, adjusted to pH 1.2) showed that only very low levels of the impurity were formed in conditions simulating the low pH environment of the stomach. After 4 hours at 35-40 °C, only 2.4 ppm of 2,3-diFBCl (relative to the weight of SB-659032 added) was detected in simulated gastric fluid, and 2.3 ppm detected in 0.1 M hydrochloric acid. The highest clinical dose of non-enteric coated SB-659032 to be administered (250 mg per day) would correspond to a daily intake of 0.6 μg of 2,3-diFBCl (0.013 μg /kg to a 50 kg individual). *In vitro* studies conducted at pH of 4 indicate that 2,3-diFBCl is not detected suggesting that it's formation is pH dependent and not likely to be formed under neutral or alkaline physiological conditions. Hence, in the current study, the dose of 250 mg of non-enteric coated tablet of SB-659032 will be administered at the end of a low-fat breakfast. As such, gastric pH is predicted to be greater than 2 when SB-659032 is administered and it is anticipated that formation of 2,3-diFBCl is likely to be negligible.

The negative result of the *in vivo* rat micronucleus assay for the related degradant, 4-FBCl, the level of containment provided by administration with food, and the very low maximum theoretical exposure (0.6 ìg from 250 mg tablet) together indicate that there is no significant genotoxic risk associated with the potential trace levels of 2,3-diFBCl that may result from oral administration of SB-659032.

To date, in the 1-month toxicological studies conducted in rats and dogs, the only significant histopathological finding was phospholipid accumulation. Most notable tolerability issues were related to decreased food consumption in rats, and emesis, increased salivation and loose feces in dogs at exposures greater than NOAEL.

1.3. Clinical Studies

To date, five clinical pharmacology studies with SB-659032 have been initiated. Single, oral doses up to 240 mg were evaluated in Study 001, while studies 002 and 004 assessed repeat, oral doses of SB-659032. As of 03 Dec 2004, 176 healthy (155 male, 21 female) subjects have been exposed to at least 1 oral dose of SB-659032. Of these, 114 (98 male, 16 female) subjects have been exposed to repeat, oral doses of SB-659032 ranging from 20-400 mg UID and for a duration of up to 14 days. Overall, SB-659032 was found to be safe and well tolerated in healthy subjects.

Study SB-659032/001

Single oral doses of SB-659032 were administered to healthy subjects in Study 001. The doses of SB-659032 studied were 2.5 mg, 5 mg, 10 mg, 20 mg, 40 mg, 80 mg, 120 mg, 180 mg and 240 mg. Thirty (30) adult male subjects were enrolled in the study and each subject was randomized to receive SB-659032 at up to 3 dose levels as well as a single oral dose of placebo. Safety, PD and PK assessments were made throughout the study. SB-659032 was found to be safe and well tolerated at single, oral doses up to 240 mg .

There were no deaths or serious adverse events (SAEs) reported in Study 001. Preliminary evaluation of data indicates no clinically significant changes in vital signs or clinical laboratory values following administration of single oral doses of SB-659032. The majority of the AEs reported were deemed to be "unlikely" or "not related" to study

medication. The most common adverse events (AEs) occurring after administration of SB-659032 were headaches and pain (e.g. backpain, pain at the venipuncture site on the left arm). The frequency of reported AEs was similar with SB-659032 and placebo.

One subject was withdrawn from Study 001 due to an AE (rash); this AE was not considered related to treatment with SB-659032. Another subject was withdrawn from the study based on pre-defined withdrawal criteria: the subject had AUC(0-inf) of 2131.5 ng.h/mL following a single dose of 180 mg which was higher than the stopping criteria [AUC = 2122 ng.h/mL based on the most sensitive species]. This subject was withdrawn due to meeting the withdrawal criteria set a priori; he did not experience any adverse events as a result of this exposure.

Following single oral administration of SB-659032 in the fasted state, maximum plasma concentrations (Cmax) of SB-659032 typically occurs around 4 hours post dose, as shown in Table 1. After attaining Cmax, SB-659032 plasma concentrations generally decline in a bi-exponential manner, with an apparent terminal elimination half-life ranging from 20 to 70 hours in healthy young males. Exposure to SB-659032 increased with increasing dose but in a less than dose proportional manner, with an approximately 9-fold increase in AUC for the 24-fold increase in dose between 10 to 240 mg. AUC(0-inf) and Cmax data exhibit moderate inter-subject variability (generally 25-55%).

Table 1 Preliminary SB-659032 Geometric Mean (CV%) Pharmacokinetic Parameters Following Single Oral Dose Administration of SB-659032 in Study 001

Dose	N	C_{max} (ng/mL)	AUC(0-inf) (ng.h/mL)	T_{max}[1] (hours)
2.5 mg	8	1.20<br>(28.0)	NE<br>NE	2.51<br>(1.95-4.00)
5 mg	8	2.18[4]<br>(36.4)	92.5[2]<br>(21.5)	3.50<br>(1.98-4.00)
10 mg	8	3.09<br>(34.1)	123[3]<br>(25.5)	3.54<br>(1.97-5.97)
20 mg	8	5.66<br>(34.4)	243[4]<br>(26.9)	5.97<br>(2.00-6.03)
40 mg	8	5.86[4]<br>(31.8)	298[3]<br>(43.0)	4.00<br>(2.98-9.00)
80 mg	8	13.2[4]<br>(25.2)	612[4]<br>(31.2)	5.97<br>(1.02-9.00)
120 mg	8	15.8<br>(37.1)	749<br>(31.8)	4.49<br>(1.98-6.00)
180 mg	8	26.2[4]<br>(29.5)	915[4]<br>(48.1)	3.47<br>(1.00-5.97)
240 mg	8	31.3[4]<br>(31.2)	1082[4]<br>(41.2)	2.03<br>(1.00-5.98)

1. median (range)
2. n=2
3. n=6
4. n=7
5. NE = not evaluable

Similar to the parent compound, the N-desalkylated metabolite SB-664601 reaches Cmax around 3 hours following single oral administration of SB-659032 in the fasted state, as shown in Table 2. Following Cmax, SB-664601 plasma concentrations decline rapidly, with a steeper slope as compared to the parent compound. This initial rapid decline is followed by a slow elimination phase with shallower slope than the parent compound. Following single-dose administration, SB-664601 was quantifiable for a short period of time in some subjects given doses of 10 to 240 mg of SB-659032. The terminal elimination half-life and, subsequently, AUC(0-inf) of SB-664601 were not well defined in this study due to the prolonged elimination phase and the limited quantifiable plasma concentration during that phase. Cmax of SB-664601 was approximately 30% (ranging from 10-55%) of the parent Cmax, however, since SB-664601 was quantifiable for a short period of time, SB-664601 AUC(0-t) was on average less than 10% (ranging 2-22%) of the parent AUC(0-t).

Table 2 Preliminary SB-664601 Geometric Mean (CV%) Pharmacokinetic Parameters Following Single Oral Dose Administration of SB-659032 in Study 001

Dose	N	C_{max} (ng/mL)	AUC(0-t) (ng.h/mL)	T_{max}[1] (hours)
2.5 mg	8	0.662[2]<br>NE	12.5[2]<br>NE	3.00[2]<br>NE
5 mg	8	0.554[2]<br>NE	0.804[2]<br>NE	3.02[2]<br>NE
10 mg	8	0.688[3]<br>(21.6)	2.85[4]<br>(10.2)	3.02[4]<br>(2.97-4.05)
20 mg	8	1.12<br>(38.1)	6.86[3]<br>(81.6)	2.49<br>(1.98-5.98)
40 mg	8	1.39[5]<br>(29.2)	8.35[6]<br>(49.8)	2.49<br>(1.95-4.07)
80 mg	8	3.75[6]<br>(15.7)	52.4[6]<br>(26.4)	3.00<br>(1.97-5.98)
120 mg	8	5.00<br>(42.8)	59.0<br>(47.6)	3.00<br>(1.98-6.00)
180 mg	8	10.3[6]<br>(49.1)	153[3]<br>(60.7)	3.03<br>(2.93-5.97)
240 mg	8	12.3[6]<br>(54.8)	196[3]<br>(78.6)	3.96<br>(1.98-5.98)

1. median (range)
2. n=1
3. n=5
4. n=3
5. n=6
6. n=7
7. NE = not evaluable

Preliminary pharmacodynamic data following single dose administration of SB-659032 showed a pharmacodynamic effect (as expressed by percent inhibition of plasma Lp-PLA_2 activity), with a difference from placebo at all doses studied. Plasma drug concentration and Lp-PLA_2 activity exhibited a direct-effect relationship. Percent

inhibition of plasma Lp-PLA_2 activity at 24 hours following dosing reached a plateau with single doses of ≥ 120 mg, as shown in Figure 1.

Figure 1 SB-659032/001: Preliminary Pharmacodynamic Data (Plasma Lp-PLA_2 Activity)

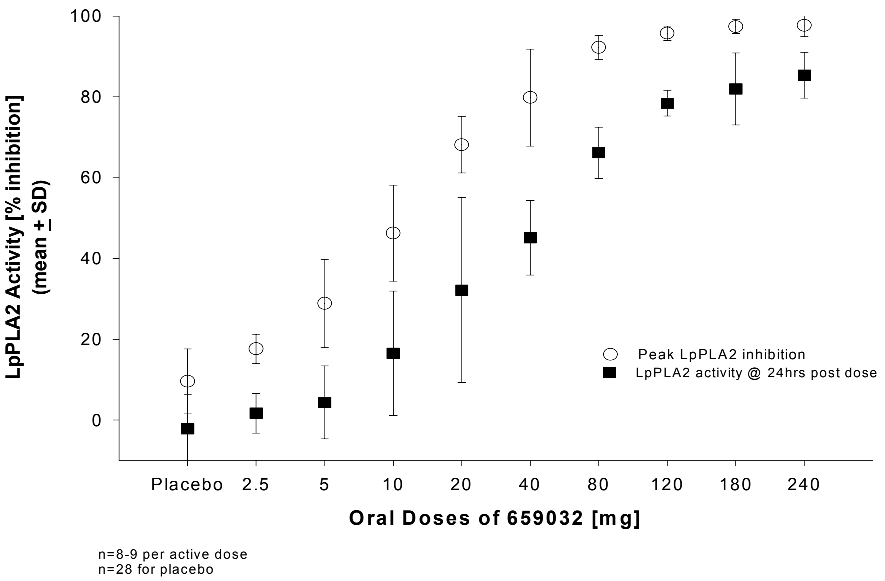

Study SB-659032/002

Single and repeat [once daily for 14 days] oral doses of SB-659032 were evaluated in Study 002. Subjects were randomized to either SB-659032 or placebo (3:1 randomization). The doses of SB-659032 administered in Study 002 were: single and repeat (once daily for up to 14 days) doses of 20 mg, 50 mg, 120 mg, 240 mg and 400 mg. Of the 61 healthy subjects enrolled 19 received single doses only, and an additional 31 received single and repeat oral doses. SB-659032 was found to be generally safe and well tolerated.

There were no deaths or serious adverse events (SAEs) reported in Study 002. Preliminary evaluation of data indicate no clinically significant changes in vital signs or clinical laboratory values following administration of single or repeat oral doses of SB-659032. The most common adverse events (AEs) occurring after administration of SB-659032 were: headache, contact dermatitis (attributed to ECGs tabs) and odor-related AEs (e.g. urine abnormal, change in body odor). Only one subject was withdrawn due to an adverse event (Bell's Palsy probably related to Herpes Simplex virus) deemed "unlikely" to be related to study medication by the investigator.

As with single dosing, Cmax and AUC increase in a less than dose proportional manner following repeat dosing of SB-659032, with a 13.4-fold increase in AUC as dose increased from 20 to 400 mg (Table 3). Preliminary pharmacokinetic (PK) data show that repeat dosing of SB-659032 up to 400 mg once daily resulted in a range of 25-100% higher plasma concentrations compared to those after a single dose. This observed increase in the systemic exposure is lower than that predicted from the single dose

pharmacokinetic data (on average, ranging from 150-230%). Steady-state SB-659032 concentrations appear to be achieved following 14 days of repeat dosing.

Table 3 Preliminary SB-659032 Geometric Mean (CV%) Pharmacokinetic Parameters Following Repeat Oral Dose Administration of SB-659032 in Study 002 Part I

Dose (mg)	Day	N	AUC(0-∞) (ng·h/mL)	AUC(0-τ) (ng·h/mL)	Cmax (ng/mL)	Tmax (h)[1]	t½ (h)
20	1	6	205 (34)	---	5.54 (16.4)	5.01 (2.02-6.07)	34.9 (20.8)
	14	6	---	98.4 (18.9)	6.48 (25.7)	4.97 (2.02-5.02)	43.1 (8.6)
50	1	6	411 (48)	---	8.87 (28.9)	4.51[2] (1.03-6.02)	40.8 (16.4)
	14	6	---	241 (19.5)	12.9 (20.1)	5.01 (1.97-6.07)	51.6 (23.8)
120	1	6	793 (77)	---	15.1 (59.8)	4.00[3] (1.08-6.00)	47.8 (19.4)[3]
	14	6	---	469 (61.7)	26.7 (66.1)	4.51 (3.00-6.05)	53.5 (17.5)
240	1	6	1400 (32)	---	33.8 (46.7)	4.98[4] (2.00-6.00)	42.4 (15.3)[4]
	14	6	---	928 (60.1)	54.9 (62.5)	4.98 (2.98-5.98)	43.2 (21.9)
400	1	5	1728 (33)	---	64.7 (44.3)	3.02[5] (2.98-5.00)	40.8 (12.7)[5]
	14	5	---	1323 (64.2)	77.1 (64.9)	5.00 (1.02-8.97)	57.2 (6.3)

Median (range)
N=8
N=7
N=10
N=6

The pharmacologically active, N-desalkylated metabolite of SB-659032, SB-664601, was not detected at 20 mg, and was sparsely quantifiable, with repeat dosing of 50 mg and 120 mg. Following administration of 240 mg and 400 mg for 14 days, SB-664601 was quantifiable to the last sampling time point (i.e., 144 hours post last dose). However, due to its long half-life, the terminal elimination phase was not fully characterized. Hence, $AUC_{(0\text{-inf})}$ and half-life were not considered reportable (Table 4). The exposure to the metabolite after repeat dose administration of 400 mg was approximately 8.4-fold higher than that observed in rats at the no toxic effect dose level. However, this exposure to the metabolite in man is less than 17% of the exposure observed in dogs at the no toxic effect dose level.

In preclinical species, SB-664601 accounts for approximately 2% (in rat) and 12.5% (in dog) of plasma concentration of the parent, SB-659032. Data from study 002 showed that in those subjects with PK profiles of measurable metabolite concentrations, the exposure to SB-664601 was less than 20% of the parent, SB-659032.

Table 4 Preliminary SB-664601 Geometric Mean (CV%) Pharmacokinetic Parameters Following Repeat Oral Dose Administration of SB-659032 in Study 002 Part I

Dose (mg)	Day	N	AUC(0-t) (ng·h/mL)	AUC(0-τ) (ng·h/mL)	Cmax (ng/mL)	Tmax (h)[1]
20	1	2	3.40 (173)	NA	0.934 (44.4)	4.02 (2.02-5.02)[2]
	14	2	4.08 (7.46)	NA	0.955 (6.59)	3.49 (2.02-4.95)
50	1	6	17.6 (61.1)	28.9 (NA)[3]	2.51 (17.7)	3.03 (2.00-5.02)[4]
	14	6	76.7 (60.1)	24.8 (30.6)	2.02 (33.4)	2.99 (1.97-4.00)
120	1	6	31.5 (126)	41.4 (19.8)[5]	3.59 (71.2)	1.98 (1.07-2.02)[4]
	14	6	180 (33.1)	45.8 (55.2)	3.45 (95.9)	2.99 (2.00-6.05)
240	1	6	201 (43.4)	91.2 (43.7)[6]	11.9 (48.2)	3.00 (2.00-5.02)[6]
	14	6	402 (49.6)	128 (72.0)	10.4 (87.4)	3.51 (1.95-5.00)
400	1	5	266 (39.8)	135 (41.5)[7]	17.0 (57.0)	3.53 (2.98-6.00)[7]
	14	5	473 (51.5)	162 (66.4)	13.2 (78.2)	3.00 (2.00-5.00)

1. Median (range)
2. N=5
3. N=1
4. N=7
5. N=4
6. N=10
7. N=6

The pharmacokinetics of a single immediate-release, oral dose of 120 mg (2 x 50 mg plus 1 x 20 mg tablets) of SB-659032 was examined under fasting condition and following a high-fat meal in healthy adults. The maximum plasma concentration (Cmax) and the systemic exposure (AUC(0-inf)) of SB-659032 increased by 23% and 41%, respectively, following a high-fat meal relative to the fasted state (Table 5 and Table 7). For the metabolite SB-664601, Cmax and the systemic exposure (AUC(0-24)) were approximately 25% and 10% lower, respectively, following a high fat meal relative to the fasted state (

Table 6 and Table 8). Time to maximum plasma concentration (Tmax) was slightly delayed, on average, by 0.24 hours for the parent and by 0.47 hours for the metabolite in the fed state, although Tmax was fairly variable.

Table 5 Preliminary SB-659032 Geometric Mean (CV%) Pharmacokinetic Parameters Following Single Oral Administration of 120 mg of SB-659032 Under Fasted and Fed Conditions in Study 002 Part II

Regimen	N	AUC(0-∞) (ng·h/mL)	AUC(0-τ) (ng·h/mL)	Cmax (ng/mL)	Tmax (h)[1]	t½ (h)
Fasted	12	732 (42)	255 (31.7)	17.7 (33.3)	5.00 (2.05-6.00)	41.4 (19.6)
Fed	12	1033 (35)	343 (25.5)	21.9 (22.7)	5.98 (1.00-9.00)	42.0 (20.1)

1. Median (range)

Table 6 Preliminary SB-664601 Geometric Mean (CV%) Pharmacokinetic Parameters Following Single Oral Administration of 120 mg of SB-659032 Under Fasted and Fed Conditions in Study 002 Part II

Regimen	N	AUC(0-t) (ng·h/mL)	AUC(0-τ) (ng·h/mL)	Cmax (ng/mL)	Tmax (h)[1]
Fasted	12	47.5 (77.4)	43.6 (34.4)[2]	4.51 (63.7)	3.00 (1.97-6.02)
Fed	12	58.0 (45.3)	39.6 (24.6)[2]	3.36 (31.9)	5.51 (1.98-9.02)

1. Median (range)
2. N=11

Table 7 Comparison of Interests: Fed versus Fasted for SB 659032 Following Single Oral Administration of 120 mg of SB-659032 in Study 002 Part II

Parameter	Comparison	PE	90% C.I.	CVw(%)
AUC(0-inf)[1]	Fed: Fasted	1.41	(1.22, 1.63)	19.7%
Cmax[1]	Fed: Fasted	1.23	(1.07, 1.42)	19.5%
Tmax[2]	Fed-Fasted	1.24	(0.02, 2.45)	

1. Ratio of adjusted geometric means between regimen groups
2. Represents the adjusted median difference

Table 8 Comparison of Interests: Fed versus Fasted for SB-664601 Following Single Oral Administration of 120 mg of SB-659032 in Study 002 Part II

Parameter	Comparison	PE	90% C.I.	CVw(%)
AUC(0-τ)[1]	Fed: Fasted	0.91	(0.73, 1.15)	29.8%
AUC(0-t)[1]	Fed: Fasted	1.22	(0.85, 1.77)	53.2%
Cmax[1]	Fed: Fasted	0.75	(0.55, 1.01)	43.2%
Tmax[2]	Fed-Fasted	1.47	(0.00, 2.51)	

1. Ratio of adjusted geometric means between regimen groups
2. Represents the adjusted median difference

Preliminary pharmacodynamic data from study 002 suggest that SB-659032 inhibited plasma Lp-PLA_2 activity (expressed as percent inhibition) in a dose-related manner (Figure 2). Single and repeat oral doses of 240 and 400 mg resulted in an approximately 94% inhibition of plasma Lp-PLA_2 activity at peak and approximately 91% (240 mg) and 94% (400 mg) at trough following repeat dosing.

Figure 2 **SB-659032/002: Preliminary Pharmacodynamic Data (Plasma $Lp\text{-}PLA_2$ Activity) Following Single and Repeat Oral Doses of SB-659032**

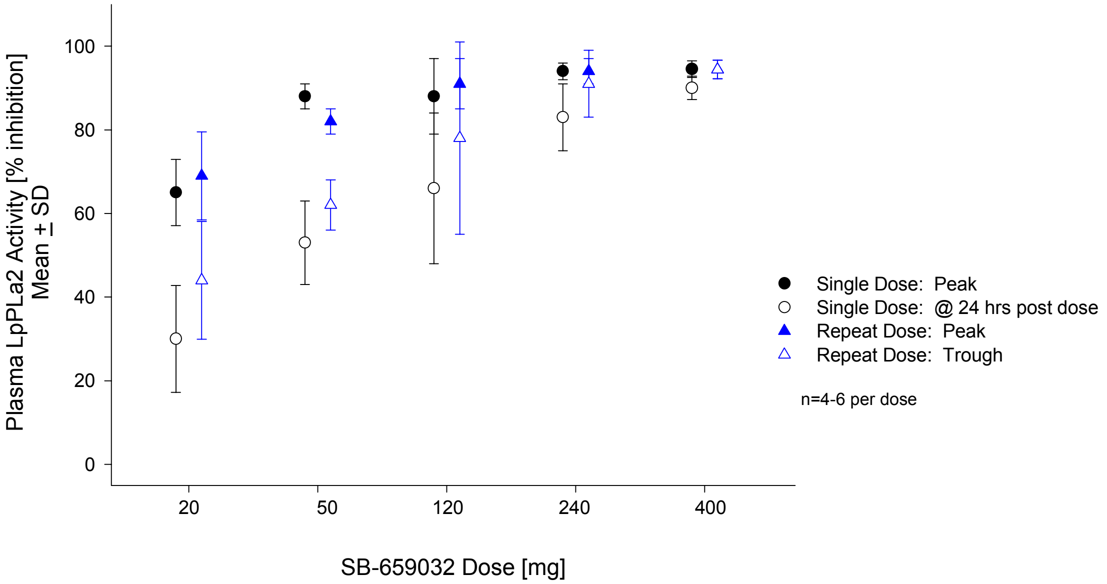

SB-659032/004

Repeat [once daily for 14 days] oral doses of SB-659032 were administered in Study 004 to evaluate the effect age and gender on the pharmacokinetics of SB-659032. Subjects were randomized to either 120 mg SB-659032 or placebo (2:1 randomization). A total of 32 subjects were exposed to at least one dose of SB-659032 in this study. SB-659032 was found to be generally safe and well tolerated.

There were no deaths or serious adverse events (SAEs) reported in Study 004. One subject receiving SB-659032 was withdrawn from the study due to an AE (dysphagia) which was considered unrelated to study drug.

There were no clinically significant changes in vitals signs reported in subjects dosed with SB-659032. The following changes in clinical laboratory parameters were reported as AEs: increase in blood creatinine and urea (n=1, active), neutropenia (n=1, active; n=1, placebo), thrombocytopenia (n=2, active; n=1, placebo) and white blood cell count decreased (n=1, active; n=1, placebo). The most common adverse events (AEs) reported following administration of SB-659032 were: headaches and changes in odors (body, feces or urine) and taste.

Following repeat-dose administration, Cmax and systemic exposure (AUC(0-24)) of SB-659032 were slightly lower in elderly male than in young male subjects (on average, 12% and 16%, respectively) (Table 9). For the pharmacologically active metabolite, SB-664601, elderly males also exhibited slightly lower Cmax and (AUC(0-24)) than young male subjects (on average, 6% and 16%, respectively) (Table 10).

Table 9 Point Estimates and Confidence Intervals for AUC(0-24) (ng.h/mL) and Cmax (ng/mL) of SB 659032 Following 14-day, Once-Daily Dosing of 120 mg of SB-659032

Comparison	Parameter	Point Estimate[1]	90% CI
EM:YM[2]	AUC(0-24)	0.84	(0.58 , 1.21)
EM:YM	Cmax	0.88	(0.61 , 1.27)
**EF:EM**	AUC(0-24)	1.35	(0.93 , 1.96)
**EF:EM**	Cmax	1.43	(0.99 , 2.09)

1. Represents the ratio of geometric means between groups
2. Elderly Males: Young Males

Table 10 Point Estimates and Confidence Intervals for AUC(0-24) (ng.h/mL) and Cmax (ng/mL) of SB-664601

Comparison	Parameter	Point Estimate[1]	90% CI
EM:YM[2]	AUC(0-τ)	0.94	(0.74 , 1.20)
EM:YM	Cmax	0.84	(0.53 , 1.35)
**EF:EM**	AUC(0-τ)	1.46	(1.14 , 1.87)
**EF:EM**	Cmax	1.87	(1.15, 3.02)

1. Represents the ratio of geometric means between groups
2. Elderly Males: Young Males

Following repeat dose administration, Cmax and (AUC(0-24)) of SB-659032 were modestly higher in elderly female than elderly male subjects (on average, 43% and 35%, respectively) (Figure 3 and Table 11). As with the parent compound, elderly females exhibited modestly higher exposure of SB-664601 than elderly male subjects (Table 12). Age/gender effects appeared to be influenced by two statistical outliers who had unusually low Cmax and AUC data (one young male and one elderly male).

Figure 3 SB-659032/004: Comparison of Plasma AUC by Cohort

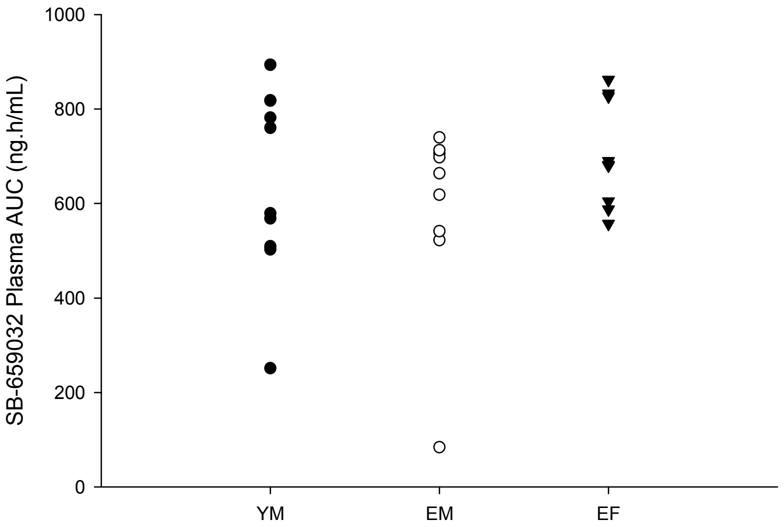

Table 11 Preliminary SB-659032 Geometric Mean (CV%) Pharmacokinetic Parameters Following Repeat Oral Administration of 120 mg of SB-659032 in Study 004

Age Group	N	AUC(0-τ) (ng·h/mL)	Cmax (ng/mL)	Tmax (h)[1]	T1/2 (h)
Young Males	10	613 (39.3)	33.9 (42.2)	5.00 (2.00, 9.00)	52.3 (19.2)
Elderly Males	9	515 (78.3)	29.8 (77.2)	5.00 (1.00, 6.00)	58.3 (14.5)[2]
Elderly Females	9	694 (16.2)	42.8 (15.4)	3.00 (1.00, 9.00)	54.6 (12.7)[2]

1. Median (range)
2. N=8

Table 12 Preliminary SB-664601 Geometric Mean (CV%) Pharmacokinetic Parameters Following Repeat Oral Administration of 120 mg of SB-659032 in Study 004

Age Group	N	AUC(0-τ) (ng·h/mL)	Cmax (ng/mL)	Tmax (h)[1]
Young Males	10	83.3 (44.2)	7.33 (62.0)	3.00 (2.00, 18.00)
Elderly Males	9	78.4 (19.6)[2]	6.17 (97.4)	2.00 (2.00, 5.00)
Elderly Females	9	115 (18.7)	11.5 (29.7)	2.00 (2.00, 6.00)

1. Median (range)
2. N=8

The potential of SB-659032 inducing its own metabolism through CYP3A4 was inferred from the urinary 6β-hydroxycortisol to cortisol ratio assessed by performing 24-hour urine collection conducted in the above age/gender study. Based on the point estimates, treatment groups showed a slight increase in the difference of the ratio of 6β-hydroxycortisol to cortisol urinary concentrations between Day 14 and Day -1 (on average, by 23%), whereas the placebo group showed an unexpected decrease (on average, by 57%). Given the substantial variability seen in this assessment (90% confidence interval ranged from 0.45 to 2.01 in the treatment group and -0.77 to 1.62 in the placebo group), a conclusive interpretation of these results is difficult.

SB-659032/902

Repeat [once daily for 14 days] 400 mg oral doses of SB-659032 were administered to healthy subjects in Study 902 to evaluate the frequency of odor-related events with non-bioenhanced, enteric-coated, free-base formulation of SB-659032 relative to placebo, in healthy adult subjects. Subjects were randomized to one of two treatment sequences (AB:BA=1:1 where A is SB-659032 and B is placebo). A total of 51 subjects were exposed to at least one dose of SB-659032 in this study. SB-659032 was found to be generally safe and well tolerated.

One subject receiving placebo experienced two serious adverse events (chest pain and hypoaesthesia) and withdrew from the study. An additional subject receiving SB-659032 withdrew from the study due to an AE (abdominal discomfort) which was considered unrelated to study drug.

There were no clinically significant changes in vitals signs reported in subjects dosed with SB-659032. The most common adverse events (AEs) reported following administration of SB-659032 were: dry skin, dry lip, headache, and dysgeusia. The most common odor-related events were dysgeusia (n=14, active; n=5, placebo), abnormal skin odor (n=9, active; n=5, placebo), and abnormal urine odor (n=2, active; n=4, placebo). Preliminary PK analysis showed that repeat dosing of 400 mg enteric-coat free-base formulation of SB-659032 resulted in AUC(0-24) of 233 ng.h/mL and Cmax of 11.1 ng/mL with variable Tmax ranging from 0 to 24 hours post-dose. This exposure is less than 20% of that from the non enteric-coat, free-base formulation of SB-659032 given at the same dose.

LP2102487

Single 250 mg oral doses of SB-659032 were administered to healthy subjects in Study LP2102487 to assess the effect of non-bioenhanced, non-enteric coated, free base SB-659032 on platelet function. Each subject received one dose of 250 mg non-enteric coated SB-659032 as a single tablet following a low fat breakfast. A total of 14 subjects were exposed to at least one dose of SB-659032 in this study.

There were no deaths or serious adverse events (SAEs) reported in Study LP2102487. All 14 subjects exposed to SB-659032 completed the study. Adverse events reported following administration of SB-659032 were: headache (n=2), abdominal pain (n=1), and urinary track infection (n=1). Only headache was considered related to study drug.

Preliminary data suggest that 11 out of 14 subjects had enhanced platelet aggregation (>15% change from baseline) at 6 hours post-dose following the addition of 0.1 or 0.2 mcg/mL of the agonist collagen. In addition, 3 out of the 14 subjects displayed enhanced platelet aggregation at 24 hours post-dose and 8 out of the 14 subjects displayed enhanced platelet aggregation at the 14 day follow-up visit. Preliminary PK analysis showed that following 250 mg single dose non enteric-coat free-base formulation of SB-659032, maximal plasma concentration occurred at approximately 4 hours post-dose and was on average 62.1 ng/mL. Due to incomplete sampling, AUC(0-inf) was not estimated.

1.4. Rationale

SB-659032 is a selective and orally active inhibitor of lipoprotein-associated phospholipase A_2 [Lp-PLA_2] that is being developed for the treatment of atherosclerosis. Lp-$PLA_{2,}$ also known as platelet activating factor acetylhydrolase (PAF-AH), is one of the enzymes responsible for the hydrolysis of PAF [platelet activating factor] into biologically inactive lyso-PAF. PAF is synthesized by a variety of cell types including platelets, neutrophils, monocytes, mast cells and eosinophils. In platelets, PAF promotes aggregation by stimulating a G protein-linked cell surface receptor. However, it does not function as an independent mediator of aggregation [Morrow, 2001].

SB-659032 will be studied in patients with documented evidence of coronary heart disease including patients with acute coronary syndrome where the use of antiplatelet agents such as aspirin and/or clopidogrel is considered part of standard care. Aspirin blocks the production of thromboxane A2, a labile inducer of platelet aggregation. Clopidogrel is a P2Y12 receptor antagonist and irreversibly inhibits adenosine 5'-diphosphate (ADP) induced platelet aggregation [Plavix Package Insert, 2003]. It is theoretically possible that an Lp-PLA_2 inhibitor may result in accumulation of PAF, promoting platelet aggregation. In a clinical study (study LP2102487) in which healthy subjects were administered a single 250 mg dose of the compound SB-659032, preliminary data [data on file] suggest that 11 out of 14 subjects had enhanced platelet aggregation (>15% change from baseline) at 6 hours post-dose following the addition of 0.1 or 0.2 mcg/mL of the agonist collagen. In addition, 3 out of the 14 subjects displayed enhanced platelet aggregation at 24 hours post-dose and 8 out of the 14 subjects displayed enhanced platelet aggregation at the 14 day follow-up visit. In the absence of a placebo control in this trial, the clinical significance of these results is unclear, highlighting the need to complete a placebo-controlled study. Therefore, this study is designed to assess the effect of SB-659032 on platelet function compared to placebo when administered daily for 14 days.

The current study is designed to assess whether inhibition of plasma Lp-PLA_2 activity impacts platelet function as assessed by *ex vivo* platelet aggregation tests and *in vivo* plasma biomarkers (ie, urine-11-dehydro-TxB2 and serum sCD40L) as compared to placebo. The stable thromboxane metabolite 11-dehydrothromboxane B2 reflects *in vivo* platelet activation [Catella, 1987] and will be measured in urine in this study. The CD40 ligand has been found expressed on CD4+ T cells and on activated platelets. Soluble CD40 ligand (sCD40L) has been implicated in acute coronary syndromes and elevated sCD40L predicts an increased risk of future cardiovascular events in healthy subjects

[Cipollone, 2002]. *Ex vivo* platelet aggregation tests will be performed using ADP and collagen as agonists. Platelet activation *in vivo* probably involves a combination of agonists, with collagen playing a more important role at the initiation of platelet adhesion, ADP playing a more important role in thrombus propagation, and with the other agonists in varying mixture throughout. Platelet-activating agonists such as ADP and collagen are added to the sample of platelet rich plasma (PRP) to initiate platelet aggregation [McKenzie, 1999]. The two agonists ADP and collagen were chosen in the current study because they stimulate aggregation through different pathways [Beutler, 1995].

Non-enteric coated, 250 mg repeat doses of SB-659032 will be utilized in this study. This dose will be administered with a low-fat breakfast in an attempt to minimize generation of 2,3-difluorobenzylchloride in the stomach. *Ex vivo* platelet aggregation tests using ADP and collagen as agonists will be performed at pre-dose, 6 hours post-dose, 24 hours post-dose on Day 1 and 14, and at the 21 day (minimum) post-last dose. They will also be performed at the equivalent time of 6 hours post-dose on Day -1. Considering the mode of action of aspirin and clopidogrel, the use of ADP and collagen as agonists for the purposes of assessing platelet function in the current study are deemed appropriate to ascertain whether an Lp-PLA_2 inhibitor, SB-659032, can negate the effect of these anti-platelet agents. Soluble CD40 ligand (sCD40L) in serum will be measured at the same time points as the platelet aggregation tests on Day 1 and 14 and at the 21 day (minimum) post-last dose. Urinary 11-dehydrothromboxane B2 (U-11-dehydro-TxB2) will be measured from a 24 hour urine collection on Days -1, 1, 14 and at the 21 day (minimum) post-dose visit.

2. OBJECTIVE(S)

2.1. Primary

1. To estimate the effect of single and repeat oral doses of 250 mg of non-enteric coated SB-659032 on platelet aggregation as compared to placebo
2. To assess the effect of single and repeat oral doses of 250 mg of non-enteric coated SB-659032 on biomarkers of platelet function as compared to placebo

2.2. Secondary

1. To evaluate the degree of inhibition of plasma Lp-PLA_2 activity following single and repeat dosing of non-enteric coated SB-659032
2. To assess the safety and tolerability of a non-enteric coated SB-659032
3. To monitor mean concentrations of SB-659032 and its major metabolite, SB-664601
4. To assess the frequency and intensity of odor-related adverse events as reported by subjects following repeated dosing with a non-enteric coated formulation of SB-659032 in healthy subjects

3. ENDPOINT(S)

3.1. Primary

1. Percent maximum platelet aggregation following ADP- and collagen-induced aggregation
2. Urinary 11-dehydrothromboxane B2 and serum soluble CD40 ligand concentrations

3.2. Secondary

1. Plasma Lp-PLA_2 activity, expressed in terms of percent inhibition relative to baseline
2. Clinical safety data from spontaneous adverse event reporting, 12-Lead ECG, vital signs, nursing/physician observation and safety laboratory tests will be summarized and any clinically significant abnormalities will be described
3. Geometric mean concentrations of SB-659032 and its major metabolite, SB-664601, will be summarized in a tabular form and descriptive statistics will be calculated.
4. The frequency and intensity of odor-related adverse events as reported by subjects will be summarized in a tabular form and descriptive statistics will be calculated.

4. STUDY DESIGN

This will be a double blind, repeat dose, randomized, placebo-controlled, period balanced, crossover study. The entire study will consist of a minimum of 12 overnight stays and 20 outpatient visits. There will be a minimum of a 21 day washout period between dosing in each period.

Regimen	Description
A	250 mg non-enteric coated SB-659032 QD for 14 days
B	SB-659032 matched placebo QD for 14 days

Subjects will return for an assessment, at a minimum of 14 days following the last dose of study medication in each period. This will require an additional 2 night stay, unless these assessments are performed at the same time as the Day -1 procedures for Period 2. The 21 day (minimum) post-last dose visit for Period 2 will also be the last follow-up visit. Each subject's participation in the study will be approximately 13 weeks from screening to follow-up.

5. STUDY POPULATION

5.1. Number of Subjects

A sufficient number of subjects will be enrolled so that approximately 20 subjects complete the study.

5.2. Eligibility Criteria

5.2.1. Inclusion Criteria

A subject will be eligible for inclusion in this study only if all of the following criteria apply:

1. Healthy adult males between 18 and 55 years of age, inclusive
2. Body weight > 50 kg (110 pounds) and body mass index (BMI) between 19 and 32 where:

$$BMI = \frac{\text{weight in kg}}{(\text{height in meters})^2}$$

3. A signed and dated written informed consent prior to admission to the study
4. The subject is able to understand and comply with protocol requirements, instructions and protocol-stated restrictions.

5.2.2. Exclusion Criteria

A subject will not be eligible for inclusion in this study if any of the following criteria apply:

1. Any clinically relevant abnormality identified on the screening medical assessment, laboratory examination or ECG
2. Platelet count below or above the reference range
3. History of hypercoagulable state or history of thrombosis
4. History of platelet dysfunction
5. A known history of Gilbert's Syndrome
6. History of asthma, anaphylaxis or anaphalactoid reactions, severe allergic responses
7. A history of alcohol, substance or drug abuse within the last year or a positive alcohol breath test at screening or predose in each period. Abuse of alcohol is defined as an average weekly intake of greater than or equal to 21 units (male) or an average daily intake of greater than or equal to 3 units (male). 1 unit is equivalent to a 285mL glass of full strength beer or 425 mL schooner of light beer or 1 (30 mL) measure of spirits or 1 glass (100 mL) of wine
8. Positive urine drug screen at screening or predose in each period
9. History of use of tobacco or nicotine containing products within 6 months of screening or a positive urine cotinine at screening or exhaled carbon monoxide test at predose in each period
10. Positive HIV, Hepatitis B or Hepatitis C at screening
11. Use of aspirin, aspirin-containing products, non-steroidal anti-inflammatory agents or any antiplatelet medication within 14 days prior to Day -1 of the study (a list of

these drugs will be reviewed with the subject at screening and provided to them to take home)

12. Use of prescription (including hormone replacement therapy) or non-prescription drugs and vitamins within 7 days or 5 half-lives (whichever is longer) prior to Day -1 of the study. An exception is acetaminophen which is allowed at doses of ≤ 2g/day
13. Use of dietary/herbal supplements including (but not limited to) St. John's wort, kava, ephedra (ma huang), gingko biloba, DHEA, yohimbe, saw palmetto, ginseng and red yeast rice within 14 days prior to Day -1 of the study
14. Treatment with an investigational drug within 30 days or 5 half-lives (whichever is longer) prior to dosing
15. Consumption of grapefruit or grapefruit juice within 7 days prior to Day -1 of the study
16. A history of cholecystectomy or biliary tract disease including a history of liver disease with elevated liver function tests of known or unknown etiology
17. An unwillingness to abstain from sexual intercourse with pregnant or lactating women or an unwillingness to use a condom and another form of contraception (e.g., IUD, birth control pills taken by female partner, diaphragm with spermicide) if engaging in sexual intercourse with a woman who could become pregnant until discharge from the study
18. Donation of blood in excess of 500 mL within 56 days or donation of blood in excess of 250 mL within 7 days prior to dosing
19. Full ADP- and/or collagen-induced aggregation (≥ 40%) at all three concentrations of one or both agonists, as assessed within 6 months prior to first dose and at Day –1 of each period
20. No ADP- or collagen-induced aggregation (<40%) at the highest concentration of either agonist, as assessed within 6 months prior to first dose and at Day –1 of each period

5.2.3. Other Eligibility Criteria Considerations

To assess any potential impact on subject eligibility with regard to safety, the investigator must refer to the Investigator’s Brochure for detailed information regarding warnings, precautions, contraindications, adverse events, and other significant data pertaining to the investigational product being used in this study. [GlaxoSmithKline Document Number ZM2004/00059/00]

6. STUDY ASSESSMENTS AND PROCEDURES

Study assessments and procedures will be performed by qualified study personnel as described in the Time and Events Table (Appendix 1).

6.1. Demographic and Baseline Assessments

Subjects will be screened within 30 days prior to administration of study medication to confirm that they meet the entrance criteria for the study. A study investigator or sub-investigator will discuss with each subject the nature of the study, its requirements, and its restrictions. Written informed consent must be obtained prior to performance of any protocol specific procedures.

6.1.1. Screening

The following procedures will be performed:

- Complete medical history
- Complete medication history of all drugs taken at least 30 days prior to screening procedures
- Complete tobacco history, including the type (e.g., pipe, cigar, chewing tobacco, or cigarette), quantity, and duration of use
- Alcohol breath test
- Physical examination including height, weight, blood pressure (BP), and heart rate (HR)
- Standard 12-lead electrocardiogram (ECG), supine
- Following a fast of at least 4 hours, blood (approximately 23 mL) and urine samples will be obtained for clinical laboratory tests,(Appendix 2: Clinical Laboratory tests), including:
  - HIV, Hepatitis B surface antigen and Hepatitis C antibody
  - Urine drug screen
  - Urine cotinine
  - PT and PTT
- Blood sample (approximately 18 mL) for platelet aggregation assessment (unless assessment was previously performed within 6 months of first dose)

To prepare for study participation, subjects will be instructed on the use of Concomitant Medications and Lifestyle Guidelines.

6.1.2. Day -1 and Predose

Subjects will report to the research facility at least 48 hours prior to the first dose of study medication in each period. Blood samples for baseline platelet aggregation and urine samples for urine-11-dehydro-TxB2 levels will be collected on Day -1 at the times described in Sections 6.6 and 6.7.

The following procedures will be performed prior to dosing with SB-659032 on Day 1:

- Update prior/concomitant medications
- Brief physical examination including vital signs, supine
- Baseline signs and symptoms assessment/adverse event assessment
- Standard 12-Lead electrocardiogram (ECG), supine
- Urine drug screen
- Alcohol breath test
- Exhaled carbon monoxide
- Blood sample (approximately 2 mL) for PK analysis
- Blood sample (approximately 3 mL) for Lp-PLA$_2$ analysis
- Blood sample (approximately 18 mL) for platelet aggregation
- Blood sample (approximately 3 mL) for sCD40L analysis
- Subjects will be asked to void to mark the beginning of the 0-24 hour urine collection for U-11-dehydro-TxB2 analysis

6.2. Dosing/Post-Dose Procedures

On the morning of Day 1, subjects will be randomized to receive either 250 mg dose of SB-659032 or SB-659032 matched placebo for 14 days. Subjects must have negative drug, alcohol and tobacco screening tests prior to dosing. A low-fat breakfast will be provided 0.5 hour prior to dosing. Study medication will be administered orally with 240 mL of tepid water by study personnel at approximately 0800 hours. Subjects will be released from the facility following dosing on Day 2. On Days 3 to 12, subjects will return for outpatient dosing of SB-659032. On the evening of Day 12, subjects will be re-admitted to the facility and will remain in house for the remainder of the period.

Blood samples for pharmacokinetic analysis, platelet aggregation, Lp-PLA$_2$ analysis and sCD40L analysis will be collected at predose and at 6 and 24 hours after dosing on Day 1 and Day 14 as described in Sections 6.5, 6.6 and 6.7. Samples will be collected either via direct venipuncture or through a catheter kept patent with 0.9% sodium chloride solution (**heparin will not be used in this study**). For the samples collected for platelet aggregation analysis, the first few mL of blood should be discarded at each collection. These blood samples should never be allowed to cool to lower than room temperature. Urine samples, for U-11-dehydro-TxB2 analysis, will be collected between 0-6, 6-12 and 12-24 hour on Day 1 and 14 as described in Section 6.7.1.

At the end of each period, questionnaires will be completed to evaluate the frequency of odor-related AEs with non-enteric coated formulation of SB-659032 relative to placebo (refer to Section 6.8).

All subjects will return for a post-dose visit at a minimum of 21 days following the last dose of study medication in Periods 1 and 2. This will require an additional 2 night stay

for Period 1, unless the Period 1 post-dose assessments are performed at the same time as the Day -1 procedures for Period 2, and an additional 2 night stay for Period 2. For both periods and for all subjects, this visit should occur at exactly the same number of days following the last dose of SB-659032. Blood samples will be collected for platelet aggregation at the equivalent times to the sample collection on Days 1 and 14 of dosing. Blood samples for Lp-PLA_2 and sCD40L analysis will be collected, for one assessment, at the time of the first platelet aggregation assessment at the 21 day (minimum) post-last dose visit. Urine samples (0-6, 6-12 and 12-24 hour), for U-11-dehydro-TxB2 analysis, will also be collected. Subjects will be asked to return to the facility at least 24 hours prior to these assessments in order to ensure that they are following a controlled diet (refer to Section 7.1).

A blood sample (approximately 10 mL) will be taken at the earliest opportunity during the study, after informed consent is obtained, for subjects who chose to participate in the pharmacogenetic research.

6.3. Follow-up Visit

The 21 day (minimum) post-last dose visit for Period 2 will also be the last follow-up visit. Subjects will undergo vital signs measurement, 12-Lead ECG, blood and urine collection for clinical laboratory tests and an AE assessment.

6.4. Safety

Safety assessments will be made at the times indicated in Appendix 1: Time and Events Table.

6.4.1. 12-Lead ECG Monitoring

All 12-Lead ECGs will be obtained after the subject has rested in the supine position for at least 10 minutes. The 12-Lead ECG tracings will be printed at a paper speed of 25 mm/sec in a '3 x 4' format. Whenever 12-Lead ECGs are performed at the same nominal time as a blood draw or heart rate and blood pressure measurement, the 12-Lead ECG will be obtained first, followed by vital signs and then blood draws. Measurements will be performed at screening, prior to dosing in each period, prior to discharge from each period, at the follow-up visit and as required throughout the study. All ECGs must be evaluated for safety by the investigator or designee.

6.4.2. Vital Sign and Clinical Laboratory Assessment

Heart rate and blood pressure measurements (supine) will be obtained after the subject has been rested for at least 5 minutes. Vital sign measurements will be performed at screening, prior to dosing in each period, at the follow-up visit and as necessary throughout the study. Clinical laboratory assessments will be performed at screening, on Day -1 of each period, at the follow-up visit, and as necessary throughout the study. Vitals signs and laboratory values of potential clinical concern (Appendix 3: Values of Potential Clinical Concern) will be reported.

6.4.3. Pregnancy

6.4.3.1. Time period for collecting pregnancy information

Details of all pregnancies which occur during the treatment and the follow-up period must be documented and reported to GSK (this is the same period over which AE information is collected – see Section 11.3). In addition, any pregnancies brought to the attention of the investigator after this period, and where it is known that study medication was taken within **30 days** prior to or at the time of conception, must also be reported.

Any pregnancies identified during the screening phase/prior to study medication administration do not need to be collected.

6.4.3.2. Action to be taken if pregnancy occurs in a female partner of a male study subject

The investigator will attempt to collect pregnancy information on any female partner of a male study subject who becomes pregnant while participating in this study. The investigator will record pregnancy information on the appropriate form and submit it to GSK within 2 weeks of learning of the partner's pregnancy. The partner will also be followed to determine the outcome of the pregnancy. Information on the status of the mother and child will be forwarded to GSK. Generally, follow-up will be no longer than 6 to 8 weeks following the estimated delivery date. Any premature termination of the pregnancy will be reported.

6.5. Pharmacokinetics

6.5.1. Sample Collection and Preparation

Blood samples (approximately 2 mL) for pharmacokinetic analysis will be collected either via direct venipuncture or through a catheter kept patent with 0.9% sodium chloride solution (**heparin will not be used in this study**) and will be processed as described in the Study Reference Manual (SRM). Samples will be collected at the following nominal times: immediately prior to dosing, at 6 and 24 hours after dosing on Day 1 and Day 14.

6.5.2. Assay Methodology

Plasma samples will be assayed for SB-659032 and its pharmacologically active N-desalkylated metabolite, SB-664601, using an approved assay methodology (on file at GlaxoSmithKline). Sample analysis will be the responsibility of World Wide Bioanalysis, Drug Metabolism and Pharmacokinetics, GlaxoSmithKline.

6.5.3. Planned Pharmacokinetic Evaluation

Due to incomplete sampling, maximum observed plasma concentration (Cmax), time to Cmax (Tmax) and AUC from time .0. to time τ, where τ is the 24-hour dosing interval

[AUC(0-τ)] will not be calculated. Geometric mean concentrations for SB-659032 and SB-664601 will be calculated at each time point. The data will be summarized in a tabular form and descriptive statistics will be calculated at each timepoint.

6.6. Pharmacodynamics

6.6.1. Sample Collection and Preparation

Platelet Aggregation

Blood samples (approximately 18 mL) for platelet aggregation will be obtained and processed according to the approved methodology (on file) for analysis in platelet rich plasma (PRP). Baseline samples will be collected on Day -1 at the equivalent times of predose, 6 and 24 hours postdose planned for the dosing days. On Day 1 and Day 14, samples will be collected at predose and at 6 and 24 hours after dosing. As the 24 hour Day -1 and the predose Day 1 time point are the same, only one sample will be collected. Samples will also be collected at the equivalent times to the dosing days at the 21 day (minimum) post-last dose visit for each period (i.e. equivalent to predose, 6 and 24 hours post-dose). Blood samples will be collected and prepared as described in the SRM. These blood samples must never be allowed to cool to lower than room temperature.

Lp-PLA_2 Activity

Blood samples (approximately 3 mL) for pharmacodynamic analysis will be collected at predose and at 6 and 24 hours after dosing on Day 1 and Day 14. An additional sample will be collected, for one assessment, at the time of the first platelet aggregation assessment at the 21 day (minimum) post-last dose visit. These samples will be processed as described in the SRM.

6.6.2. Assay Methodology

Platelet Aggregation

All platelet aggregometry will be performed according to the approved methodology using platelet rich plasma (PRP). ADP and collagen will be used as agonists for the platelet-induced aggregation studies. ADP agonist will use a single dose to establish a sub-threshold concentration, while the collagen agonist will require three doses, because of the variability of individual responses to collage. Both agonists will have a high (i.e. complete aggregation) concentration. A standard aggregation curves will be determined for both agonists.

Lp-PLA_2 Activity

Plasma samples will be assayed for Lp-PLA_2 activity using an approved method under the management of GlaxoSmithKline Pharmaceuticals.

6.6.3. Planned Pharmacodynamic Evaluation

Platelet Aggregation

Details of the platelet aggregation evaluation will be provided in the RAP. All platelet aggregation tracings will be over-read and analyzed by an external blinded haematologist. This will be the final data set to be databased.

Lp-PLA$_2$ Activity

The primary parameter, plasma Lp-PLA$_2$ activity, will be summarized as percent inhibition of plasma Lp-PLA$_2$ activity using the following formula:

$$100 \times [(\text{Activity}_{\text{at pre-dose on Day 1}} - \text{Activity}_{\text{at time x}}) / \text{Activity}_{\text{at pre-dose on Day 1}}]$$

where Activity represents plasma Lp-PLA$_2$ activity.

Additional summary measures may be calculated as appropriate.

6.7. Biomarker(s)

6.7.1. Sample Collection and Preparation

sCD40L

Blood samples (approximately 3 mL), for the determination of soluble CD40 ligand will be collected at predose and at 6 and 24 hours after dosing on Day 1 and Day 14. An additional sample will be collected, for one assessment, at the time of the first platelet aggregation assessment at the 21 day (minimum) post-last dose visit. These samples will be processed as described in the SRM.

u-11-dehydro-TxB2

A 0-24 hour urine collection will be obtained on Day -1, Day 1 and 14 and at the 21 day (minimum) post-last dose visit, for the determination of urinary 11-dehydrothromboxane B2 (u-11-dehydro-TxB2). To mark the beginning of the 0-6 hour urine collection interval, subjects will be asked to void. This void will be discarded. Urine will be collected and stored at refrigerated temperature (2-8°C or 36-46°F). At the nominal time of 6 hours, subjects will be asked to void. This void will be collected as part of the 0-6 hour interval of urine collection. The end of this void marks the beginning of the 6-12 hour collection interval. At the nominal time of 12 hours, subjects will be asked to void. This void will be collected as part of 6-12 hour collection. The end of this void marks the beginning of the 12-24 hour collection interval. At the nominal time of 24 hours, subjects will be asked to void. This void will be collected as part of 12-24 hour collection. For each time period, the total volume of urine will be recorded and a sample aliquot (approximately 3 mL) will be retained. Samples will be processed as described in

the SRM. Urine creatinine will be measured to assess the adequacy of the urine collections over the 24-hour period.

6.7.2. Assay Methodology

The assays for U-11-dehydro-TxB2 and sCD40L will be performed according to approved methodology.

6.7.3. Planned Biomarker Evaluation

Details of the biomarker analysis will be provided in the RAP.

6.8. Odor Assessments

Odor-related adverse events as reported by the subjects (questionnaire #1) will be assessed using a questionnaire with a validated intensity scale [Green, 1996] at the end of each period.

6.9. Pharmacogenetics

Information regarding pharmacogenetic research is included in Appendix 4: PGx.

7. LIFESTYLE AND/OR DIETARY RESTRICTIONS

7.1. Meals and Dietary Restrictions

- Subjects must abstain from ingestion of alcohol and caffeine- or xanthine-containing products 24 hours prior to Day -1 until collection of the final blood/urine sample in each period
- Subjects will not be allowed to drink grapefruit juice or eat grapefruit within 7 days prior to Day -1 until collection of the final blood/urine sample in each period
- Water may be consumed ad libitum beginning 2 hour after dosing; soft drinks without caffeine or fruit juices (except grapefruit) may be consumed ad libitum beginning 4 hours after dosing
- Subjects must fast from all food and drink (except water) at least 4 hours prior to any clinical laboratory tests.

- Subjects should not consume the following foods/food additives, listed in the table below, for 24 hours prior to Day -1 until collection of the final blood/urine sample in each period

Omega-3 fatty acids-eicosapentaenoic acid/docosahexaenoic acid (i.e., fish oil)
Ethanol
Chinese black tree fungus (used in chinese food)
Onion extract
Garlic (ajoene, a component of garlic)
Cumin
Tumeric
Red wine
Ginger
Red Clover
Horse chestnut
Cat's claw
Dong quai
Evening primrose
Feverfew
Green tea

- All subjects will be provided with the same low fat meals prepared without the foods/food additives mentioned above during their inpatient stay. Subjects will be admitted to the facility on Day -2 and they will be served breakfast, lunch and dinner.

  On Day 1 to 14, a low-fat breakfast will be provided 0.5 hour prior to dosing (the entire meal must be consumed within 20 minutes and subjects must be dosed within less than 10 minutes after completing the meal). The meal will consist of the following:

  - Cereal (Cornflakes or Special K), 200 mL (6.7 oz) of skim milk, one strip of bacon (grilled), one egg (scrambled with skimmed milk), two slices of toast, pat of butter equivalent to 1 tsp, and one-half cup (4 oz) of fruit juice (apple, orange). This breakfast will be approximately 488 calories (approximately 77 g carbohydrate, 28 g protein, 12 g fat).

  On all inpatient days, lunch will be provided at approximately 6 hours after dosing and **also must be low fat (≤ 12 g fat)**. Dinner will be provided at approximately 10 hours after dosing (or at the equivalent time) and an evening snack will be permitted up until 2200 hours.

  Subjects will be admitted to the facility at least 24 hour prior to their post-dose assessments and will receive the same meals for breakfast, lunch and dinner. Breakfast, lunch and dinner will be served at the same approximate times as on the dosing days. An evening snack will be permitted up until 2200 hours.

7.2. Contraception

Male subjects must be willing to abstain from or use a condom during sexual intercourse with pregnant or lactating females. Male subjects must also use a condom, plus another form of contraception (e.g., spermicide, IUD, birth control pills taken by female partner, diaphragm with spermicide) if engaging in sexual intercourse with a woman who could become pregnant. Male subjects must adhere to these contraceptive criteria from administration of the first dose of study medication until discharge from the study (i.e., follow-up visit).

7.3. Other Restrictions

- Subjects should abstain from strenuous physical activity from 24 hours prior to each blood collection for clinical laboratory tests
- Subjects will abstain from tobacco use for the duration of the study
- Subjects will be asked to avoid direct exposure to natural or artificial sunlight from administration of the first dose until 3 days after the last dose. Areas of skin exposed to direct sunlight for more than 15 minutes should be protected with sunblock (SPF $\geq$ 30).

8. INVESTIGATIONAL PRODUCT(S)

8.1. Description of Investigational Product

SB-659032 will be supplied by GlaxoSmithKline as 12 mm, white, film coated, round, biconvex tablets. The non-bioenhanced, non-enteric coated, freebase formulation of SB-659032 will be available in 250 mg tablet strengths.

8.2. Dosage and Administration

Subjects will be randomized to receive study medication in accordance with the randomization schedule. Subjects will be provided a low-fat breakfast starting 0.5 prior to dosing which they must consume entirely within 20 minutes. Subjects will be administered 1 tablet by study personnel with approximately 240 mL of water.

Regimen	Description
A	250 mg non-enteric coated SB-659032 QD for 14 days
B	SB-659032 matched placebo QD for 14 days

8.3. Dose Rationale

The 250 mg dose of non-enteric coated formulation of SB-659032 was chosen based on the pharmacodynamic data (inhibition of plasma Lp-PLA2 activity) noted in Study 002 where a non-enteric coated formulation of SB-659032 was studied. In the fasted state, doses of 240 and 400 mg resulted in >90% inhibition of plasma Lp-PLA2 activity at

Cmax following a single dose. In the current study, SB-659032 will be administered with food to minimize the formation of 2,3-diFBCl and risk to the subjects. The fact that food effect study results suggest a clinically significant food effect when non-enteric coated formulation of SB-659032 is dosed with a high-fat meal, the dose of non-enteric coated SB-659032 will be 250 mg in the current study so that the exposure at 400 mg is not exceeded. Extrapolating the observed food effect at a dose of 120 mg in Study 002 and assuming that the magnitude of food effect is similar with a high-fat breakfast (administered in Study 002) and a low-fat breakfast (to be administered in current study), with the 250 mg dose planned in the current study, the observed exposure is anticipated to be within the range noted in Study 002 where repeat, oral doses up to 400 mg were found to be safe and well tolerated. The ultimate goal of Lp-PLA2 inhibition is to inhibit the activity of the enzyme within atherosclerotic lesions (the site of Lp-PLA2 hydrolytic activity). In a study to investigate the effects of SB-480848 (lead Lp-PLA2 inhibitor) on carotid plaque composition in patients with carotid artery disease who underwent carotid endarterectomy after 14 +/- 4 days of dosing, results have shown that similar levels of inhibition of Lp-PLA2 activity were observed in plasma and carotid plaque. However, no clinical information exists as to what level of intralesional Lp-PLA2 inhibition results in the downstream inhibition of inflammatory mediators. Before such a relationship is established in the target patient population, the criteria for maximal desired plasma Lp-PLA2 inhibition are arbitrary for as long as no adverse effects are seen with SB-659032.

8.4. Blinding

This is a double blind study.

Only in the case of an emergency, when knowledge of the investigational product is essential for the clinical management or welfare of the subject, the investigator may unblind a subject's treatment assignment. The investigator will, whenever possible, discuss options with the Medical Monitor, on-call physician, or appropriate GSK study personnel before unblinding. If the blind is broken for any reason and the investigator is unable to contact GSK prior to unblinding, the investigator must notify GSK **as soon as possible following** the unblinding incident **without revealing the subject's study treatment assignment,** unless the information is important to the safety of subjects remaining in the study. In addition, the investigator will record the date and reason for revealing the blinded treatment assignment for that subject in the appropriate data collection tool (as defined in Section 13.7).

If a serious adverse event (SAE; as defined in Section 11.2, "Definition of an SAE") is reported to GSK, Global Clinical Safety and Pharmacovigilance (GCSP) staff may unblind the treatment assignment for the individual subject. If an expedited regulatory report to one or more regulatory agencies is required, the report will identify the subject's treatment assignment. When applicable, a copy of the regulatory report may be sent to investigators in accordance with relevant regulations, GSK policy, or both.

8.5. Treatment Assignment

Subjects will be assigned to one of the two sequences (AB or BA) in accordance with the randomization schedule prepared prior to study start by CPSP, GSK, using a validated internal software (RandAll).

8.6. Packaging and Labeling

The contents of the label will be in accordance with all applicable regulatory requirements.

8.7. Preparation

No specific preparation of study medication is required prior to administration.

8.8. Handling and Storage

Investigational product must be dispensed or administered according to procedures described herein. Only subjects enrolled in the study may receive investigational product, in accordance with all applicable regulatory requirements. Only authorized site staff may supply or administer investigational product. All investigational products must be stored in a secure area with access limited to the investigator and authorized site staff and under physical conditions that are consistent with investigational product-specific requirements.

8.9. Product Accountability

The investigator, institution, or the head of the medical institution (where applicable) is responsible for investigational product accountability, reconciliation, and record maintenance. In accordance with all applicable regulatory requirements, the investigator or the head of the medical institution (where applicable), or designated site staff (e.g., storage manager, where applicable) must maintain investigational product accountability records throughout the course of the study. The responsible person(s) will document the amount of investigational product received from and returned to GSK (when applicable), the amount supplied and/or administered to and returned by subjects, if applicable.

8.10. Assessment of Compliance

Study medication will be administered under the supervision of study personnel. The oral cavity of each subject will be examined following dosing to assure that study medication was taken.

A record of the amount taken by the subject, together with any related information (e.g. amount dispensed or returned) will be recorded.

8.11. Treatment of Investigational Product Overdose

Any signs or symptoms of SB-659032 overdosage will be treated symptomatically. No specific antidotes are known.

8.12. Occupational Safety

Investigational product is not expected to pose significant occupational safety risk to site staff under normal conditions of use and administration. A Material Safety Data Sheet (MSDS)/equivalent document describing occupational hazards and recommended handling precautions either will be provided to the investigator, where this is required by local laws, or is available upon request from GSK.

9. CONCOMITANT MEDICATIONS AND NON-DRUG THERAPIES

9.1. Permitted Medications

All concomitant medications taken during the study will be recorded in the CRF. The minimum requirement is that drug name and the dates of administration are to be recorded.

Acetaminophen is allowed at doses of ≤ 2g/day.

9.2. Prohibited Medications

- Use of prescription or non-prescription drugs (other than acetaminophen as described above) within 7 days or 5 half-lives (whichever is longer) prior to Day -1 of Period 1 and throughout the study
- Use of vitamins within 7 days prior to Day -1 of Period 1 and throughout the study
- Use of herbal and dietary supplements within 14 days prior to Day -1 of Period 1 and throughout the study
- Use of aspirin, aspirin-containing products, non-steroidal anti-inflammatory agents or any antiplatelet medication within 14 days prior to Day -1 of Period 1 and throughout the study (a list of these drugs will be reviewed with the subject at screening and provided to them to take home)

10. SUBJECT COMPLETION AND WITHDRAWAL

10.1. Subject Completion

Subjects who complete all protocol specified procedures will be considered as having completed the study. Subjects for whom sufficient safety, PD and/or PK data have been collected will be considered as being evaluable subjects.

10.2. Subject Withdrawal

10.2.1. Subject Withdrawal from Study

A subject may withdraw from the study at any time at his/her own request, or they may be withdrawn at any time at the discretion of the investigator for safety, behavioral or administrative reasons. Subjects will undergo vital signs measurement, 12-Lead ECG, blood and urine collection for clinical laboratory tests and an AE assessment prior to discharge.

When a subject completes a visit, relevant sections of the CRF will be completed by the investigator (or designated staff).

Unevaluable subjects may be replaced with another subject assigned the same sequence of treatments.

10.2.2. Subject Withdrawal from Investigational Product

A subject may withdraw from taking the investigational product at any time at his or her own request, or they may be withdrawn at any time at the discretion of the investigator for safety, behavioral or administrative reasons.

10.3. Treatment After the End of the Study

Subjects who do not meet the inclusion/exclusion criteria, including those subjects with clinical laboratory values such as a positive urine drug screen, will be considered screen failures.

11. ADVERSE EVENTS (AE) AND SERIOUS ADVERSE EVENTS (SAE)

The investigator is responsible for the detection and documentation of events meeting the criteria and definition of an AE or SAE, as provided in this protocol. During the study when there is a safety evaluation, the investigator or site staff will be responsible for detecting, documenting and reporting AEs and SAEs, as detailed in both this section of the protocol and in the AE/SAE section of the SRM.

11.1. Definition of an AE

Any untoward medical occurrence in a patient or clinical investigation subject, temporally associated with the use of a medicinal product, whether or not considered related to the medicinal product.

Note: An AE can therefore be any unfavorable and unintended sign (including an abnormal laboratory finding), symptom, or disease (new or exacerbated) temporally associated with the use of a medicinal product.

11.2. Definition of a SAE

A serious adverse event is any untoward medical occurrence that, at any dose:

a. Results in death

b. Is life-threatening

   NOTE: The term 'life-threatening' in the definition of 'serious' refers to an event in which the subject was at risk of death at the time of the event. It does not refer to an event, which hypothetically might have caused death, if it were more severe.

c. Requires hospitalization or prolongation of existing hospitalization

   NOTE: In general, hospitalization signifies that the subject has been detained (usually involving at least an overnight stay) at the hospital or emergency ward for observation and/or treatment that would not have been appropriate in the physician's office or out-patient setting. Complications that occur during hospitalization are AEs. If a complication prolongs hospitalization or fulfills any other serious criteria, the event is serious. When in doubt as to whether "hospitalization" occurred or was necessary, the AE should be considered serious.

   Hospitalization for elective treatment of a pre-existing condition that did not worsen from baseline is not considered an AE.

d. Results in disability/incapacity, or

   NOTE: The term disability means a substantial disruption of a person's ability to conduct normal life functions. This definition is not intended to include experiences of relatively minor medical significance such as uncomplicated headache, nausea, vomiting, diarrhea, influenza, and accidental trauma (e.g. sprained ankle) which may interfere or prevent everyday life functions but do not constitute a substantial disruption.

e. Is a congenital anomaly/birth defect

f. Medical or scientific judgement should be exercised in deciding whether reporting is appropriate in other situations, such as important medical events that may not be immediately life-threatening or result in death or hospitalization but may jeopardize the subject or may require medical or surgical intervention to prevent one of the other outcomes listed in the above definition. These should also be considered serious. Examples of such events are invasive or malignant cancers, intensive treatment in an emergency room or at home for allergic bronchospasm, blood dyscrasias or convulsions that do not result in hospitalization, or development of drug dependency or drug abuse.

11.2.1. Clinical Laboratory Abnormalities and Other Abnormal Assessments as AEs and SAEs

Abnormal laboratory findings (e.g., clinical chemistry, hematology, urinalysis) or other abnormal assessments (e.g., ECG or vital signs) that are judged by the investigator as **clinically significant** will be recorded as AEs or SAEs if they meet the definition of an AE or SAE. Clinically significant abnormal laboratory findings or other abnormal

assessments that are detected during the study or are present at baseline and significantly worsen following the start of the study will be reported as AEs or SAEs. However, clinically significant abnormal laboratory findings or other abnormal assessments that are associated with the disease being studied, unless judged by the investigator as more severe than expected for the subject's condition, or that are present or detected at the start of the study and do not worsen, will **not** be reported as AEs or SAEs.

The investigator will exercise his or her medical and scientific judgment in deciding whether an abnormal laboratory finding or other abnormal assessment is clinically significant.

11.3. Time Period, and Frequency of Detecting AEs and SAEs

Any pre-existing conditions or signs and/or symptoms present in a subject prior to the start of the study (i.e. before informed consent) should be recorded as Medical/Surgical History. In addition, any medical occurrence which is reported after informed consent is obtained but prior to starting active or randomized treatment (i.e. Period 1) will be documented as Medical/Surgical History. Any signs and symptoms present at the time the first dose is administered will be documented as baseline signs and symptoms. Any medical occurrences which present after administration of study medication and on or before the final follow up visit must be reported as Adverse Events. All AEs must be recorded irrespective of whether they are considered drug related.

From the time a subject consents to participate in the study until he or she has completed the study (including any follow-up period), all SAEs assessed as related to study participation (e.g., protocol-mandated procedures, invasive tests, or change in existing therapy) or related to a GSK concomitant medication, will be reported promptly to GSK.

At each visit/assessment in the period defined above, AEs/SAEs will be evaluated by the investigator and recorded. Any AEs/SAEs already documented at a previous assessment and designated as ongoing, should be reviewed at subsequent visits as necessary. If these have resolved, this should be documented. If an AE changes in intensity then the maximum intensity should be captured in the CRF.

As a consistent method of soliciting AEs, the subject should be asked a non-leading questions such as: "How do you feel?", "Have you had any medical problems since your last visit/assessment?", and "Have you taken any new medicines, other than those provided in this study, since your last visit?" at the timepoints listed in Appendix 1: Time and Events Table.

11.4. Prompt Reporting of SAEs to GSK

SAEs will be reported promptly to GSK as described in the following table once the investigator determines that the event meets the protocol definition of an SAE.

11.4.1. Timeframes for Submitting SAE Reports to GSK

	Initial SAE Reports		Follow-up Information on a Previously Reported SAE	
Type of SAE	Time Frame	Documents	Time Frame	Documents
All SAEs	24 hrs	"SAE" data collection tool	24 hrs	Updated "SAE" data collection tool

11.5. AE and SAE Documentation and Follow-up Procedures

The investigator will review and adhere to the following procedures, which are outlined in detail in the AE/SAE section of the SRM:

- Method of Detecting AEs and SAEs
- Recording of AEs and SAEs
- Evaluating of AEs and SAEs
- Completion and Transmission of SAE Reports to GSK
- Follow-up of AEs and SAEs
- Post-study AEs and SAEs
- Regulatory Reporting Requirements for SAEs

12. DATA ANALYSIS AND STATISTICAL CONSIDERATIONS

12.1. Hypotheses

An estimation approach will be used to address the study objectives, where point estimates and corresponding 90% C.I. will be constructed to provide a range of plausible values for the comparisons of interest.

12.2. Study Design Considerations

12.2.1. Sample Size Assumptions

Sample size calculations are based on the within-subject variability estimate (SD=15.6%, maximum platelet aggregation using collagen as an agonist) obtained from the preliminary analyses [data on file] of the previously completed clinical study for SB-659032 (LP2102487).

Assuming a similar variability for percent maximum platelet aggregation for SB-659032 and a sample size of 20, the estimated half width of the 90% confidence intervals for the difference A-B will be no more than 8.5%. Also, based on this variability and a sample size of 20 subjects, it is estimated that this study will have at least 90% power to detect a difference of 15% between regimens A and B. The above calculations were based on a

two-sided t-test with a type I error rate of 10%. No adjustment was made to the type I error rate for multiple comparisons.

12.2.2. Sample Size Sensitivity

A sensitivity analysis was performed to investigate the effect of increased variability.

A sensitivity analysis was conducted in the event that the variability in maximum platelet aggregation was greater than estimated. The upper bound of 90% C.I. for the observed variability estimate (SD=23.15%) was determined. Based on this variability, it is estimated the half width of the 90% confidence intervals for the difference A-B will be no more than 12.4%. Also, based on this variability and a sample size of 20 subjects, it is estimated that this study will have at least 65% power to detect a difference of 15% between regimens A and B. The above calculations were based on a two-sided t-test with a type I error rate of 10%.

No adjustment was made to the type I error rate for multiple comparisons.

12.2.3. Sample Size Re-estimation

A sample size re-estimation will not be performed for this study.

12.3. Data Analysis Considerations

12.3.1. Analysis Populations

For each pharmacodynamic parameter separately, subjects will be included in the formal statistical analyses and summaries of the pharmacodynamic data provided that they have data from at least one regimen.

All subjects who receive at least one dose of study medication will be included in the safety data.

12.3.2. Analysis Data Sets

All subjects who receive at least one dose of study drug will be included in the safety population. This population will be used in the evaluation of tolerability.

All subjects from whom a pharmacokinetic (PK) sample has been obtained and analyzed will be included in the PK concentration population. This population will be used in the evaluation of PK concentration data.

All evaluable data will be included as appropriate in the statistical analysis. Data from subjects not included in the statistical analyses will be listed. The impact of subjects with incomplete data will be evaluated, as appropriate to the data.

12.3.2.1. Derived and Transformed Data

The pharmacodynamic parameter, plasma Lp-PLA_2 activity, will be summarized as percent inhibition of plasma Lp-PLA_2 activity using the following formula:

$$100 \times [(\text{Activity}_{\text{at pre-dose on Day 1}} - \text{Activity}_{\text{at time x}}) / \text{Activity}_{\text{at pre-dose on Day 1}}]$$

where Activity represents plasma Lp-PLA_2 activity.

12.3.3. Treatment Comparisons

12.3.3.1. Primary Comparisons of Interest

The primary focus of the statistical analysis is to estimate the effect of single and repeat oral doses of SB-659032 (regimen A) on maximum platelet aggregation and biomarkers relative to Placebo (regimen B). Thus, point estimates and 90% confidence intervals will be presented for the difference A-B.

12.3.3.2. Other Comparisons of Interest

No other comparisons of interest.

12.3.4. Interim Analysis

No interim analysis will be performed.

12.3.5. Key Elements of Analysis Plan

12.3.5.1. Safety Analyses

No formal statistical analysis of safety data is planned. Safety data will be presented in tabular and/or graphical format and summarized descriptively. AEs will be coded using the MedDRA (Medical Dictionary for Regulatory Activities) dictionary.

12.3.5.2. Pharmacokinetic Analyses

No statistical analyses of pharmacokinetic data are planned.

12.3.5.3. Pharmacodynamic Analyses

The primary pharmacodynamic parameter, change from baseline maximum platelet aggregation following ADP and Collagen will be separately analyzed by a mixed effects model, fitting fixed terms for regimen, sequence, period, Day, and Time, and subject as a random effect. Baseline may be added as a covariate to the model. Point estimates and 90% confidence intervals for the difference A-B on Days 1 and 14 and at each time point will be calculated using appropriate error term.

All secondary pharmacodynamic parameters will be analyzed using a similar statistical model appropriate to the data.

Distributional assumptions underlying the analyses will be assessed by residual plots. Homogeneity of variance will be assessed by plotting the residuals against the predicted values from the model, while normality will be examined by normal probability plots. If assumptions are grossly violated, alternative analyses will be considered.

Data listings will be generated for all subjects and descriptive statistics (n, arithmetic mean, standard deviation, minimum, median, maximum) will be calculated for all pharmacodynamic endpoints by regimen, day and time.

As appropriate, for change from baseline in platelet aggregation, assessment of evidence of carryover effect will be performed by examining the sequence effects from the above model, and also by modelling the baseline values (pre-dose data measured at the start of each treatment period). Baseline may be separately analysed by ANOVA model, similar to the above, where subjects may be treated as random effects. Also, appropriate graphical/descriptive methods may be explored to examine the possibility of any carryover effects. If there is compelling evidence of carryover effect from the above analysis, the data from sequence BA or Period 1 data will be analyzed separately using appropriate methods.

Estimates of inter and intra subject variability for platelet aggregation will be calculated from placebo and/or baseline data to interpret clinical significance. Additional details will be provided in the RAP.

12.3.5.4. Pharmacokinetics/Pharmacodynamics Analyses

No pharmacokinetic/pharmacodynamic analyses will be conducted.

12.3.5.5. Biomarker(s) Analyses

All biomarkers will be separately analyzed by a mixed effects model similar to maximum platelet aggregation, fitting fixed terms for regimen, sequence, period, day, and time, and subject as a random effect. Point estimates and 90% confidence intervals for the difference A-B on days 1 and 14 will be calculated using appropriate error term. As appropriate, baseline (pre-dose on Day 1) will be added as a covariate in the model. Further details will be provided in the RAP.

Data listings will be generated for all subjects and descriptive statistics (n, arithmetic mean, standard deviation, minimum, median, maximum) will be calculated for all biomarkers by regimen, and day.

12.3.5.6. Odor Assessment

No formal analysis will be conducted. The frequency of odor-related adverse events as reported by subjects will be summarized in a tabular form and descriptive statistics will be calculated.

13. STUDY CONDUCT CONSIDERATIONS

13.1. Regulatory and Ethical Considerations, Including the Informed Consent Process

GSK will obtain favorable opinion/approval to conduct the study from the appropriate regulatory agency in accordance with any applicable country-specific regulatory requirements prior to a site initiating the study in that country.

The study will be conducted in accordance with all applicable regulatory requirements, including an U.S. IND.

The study will also be conducted in accordance with "good clinical practice" (GCP), all applicable subject privacy requirements, and, the guiding principles of the Declaration of Helsinki. This includes, but is not limited to, the following:

- IRB/IEC review and favorable opinion/approval to conduct the study and of any subsequent relevant amended documents
- Subject informed consent
- Investigator reporting requirements

GSK will provide full details of the above either verbally, in writing or both.

Written informed consent will be obtained for each subject before he or she can participate in the study.

13.2. Quality Control (Study Monitoring)

In accordance with applicable regulations, GCP, and GSK procedures, GSK monitors will contact the site prior to the start of the study to review with the site staff the protocol, study requirements, and their responsibilities to satisfy regulatory, ethical, and GSK requirements. When reviewing data collection procedures, the discussion will also include identification, agreement and documentation of data items for which the CRF will serve as the source document.

GSK will monitor the study consistent with the demands of the study and site activity to verify that the:

- Data are authentic, accurate, and complete.
- Safety and rights of subjects are being protected.
- Study is conducted in accordance with the currently approved protocol and any other study agreements, GCP, and all applicable regulatory requirements.

The investigator and the head of the medical institution (where applicable) agrees to allow the monitor direct access to all relevant documents

13.3. Quality Assurance

To ensure compliance with GCP and all applicable regulatory requirements, GSK may conduct a quality assurance audit. Regulatory agencies may also conduct a regulatory inspection of this study. Such audits/inspections can occur at any time during or after completion of the study. If an audit or inspection occurs, the investigator and institution agree to allow the auditor/inspector direct access to all relevant documents and to allocate his/her time and the time of his/her staff to the auditor/inspector to discuss findings and any relevant issues.

13.4. Study and Site Closure

Upon completion or premature discontinuation of the study, the monitor will conduct site closure activities with the investigator or site staff, as appropriate, in accordance with applicable regulations, GCP, and GSK procedures.

In addition, GSK reserves the right to temporarily suspend or prematurely discontinue this study at any time for reasons including, but not limited to, safety or ethical issues or severe non-compliance. For multicenter studies, this can occur at one or more or at all sites. If GSK determines such action is needed, GSK will discuss this with the investigator or the head of the medical institution (where applicable), including the reasons for taking such action, at that time. When feasible, GSK will provide advance notification to the investigator or the head of the medical institution, where applicable, of the impending action prior to it taking effect.

GSK will promptly inform all other investigators or the head of the medical institution (where applicable), and/or institutions conducting the study if the study is suspended or terminated for safety reasons. GSK will also promptly inform the regulatory authorities of the suspension or termination of the study and the reason(s) for the action. If required by applicable regulations, the investigator or the head of the medical institution (where applicable) must inform the IEC/IRB promptly and provide the reason for the suspension or termination.

13.5. Records Retention

Following closure of the study, the investigator or the head of the medical institution (where applicable) must maintain all site study records, except for those required by local regulations to be maintained by someone else, in a safe and secure location. The records must be maintained to allow easy and timely retrieval, when needed (e.g., audit or inspection), and, whenever feasible, to allow any subsequent review of data in conjunction with assessment of the facility, supporting systems, and staff. Where permitted by local laws/regulations or institutional policy, some or all of these records can be maintained in a format other than hard copy (e.g., microfiche, scanned, electronic); however, caution needs to be exercised before such action is taken. The investigator must assure that all reproductions are legible and are a true and accurate copy of the original, and meet accessibility and retrieval standards, including re-generating a hard copy, if required. Furthermore, the investigator must ensure there is an acceptable

back-up of these reproductions and that an acceptable quality control process exists for making these reproductions.

GSK will inform the investigator of the time period for retaining these records to comply with all applicable regulatory requirements. The minimum retention time will meet the strictest standard applicable to that site for the study, as dictated by any institutional requirements or local laws or regulations, or GSK standards/procedures; otherwise, the retention period will default to 15 years.

The investigator must notify GSK of any changes in the archival arrangements, including, but not limited to, the following: archival at an off-site facility, transfer of ownership of the records in the event the investigator leaves the site.

13.6. Provision of Study Results and Information to Investigators

When required by applicable regulations, the investigator signatory for the clinical study report will be determined at the time the report is written. When the clinical study report is completed, GSK will provide the investigator with a full summary of the study results. The investigator is encouraged to share the summary results with the subjects, as appropriate. In addition, the investigator will be given reasonable access to review the relevant statistical tables, figures, and reports and will be able to review the results for the entire study at a GSK site or other mutually agreeable location.

GSK will provide the investigator with the randomization codes for their site after the statistical analysis for the entire study has been completed.

13.7. Data Management

The data collection tool for this study will GSK-defined case report forms (CRFs). In all cases, subject initials will not be collected nor transmitted to GSK. In all cases, subject initials will not be collected nor transmitted to GSK. Subject data necessary for analysis and reporting will be entered/transmitted into a validated database or data system. Clinical data management will be performed in accordance with applicable GSK standards and data cleaning procedures. Original CRFs will be retained by GSK, while the investigator will retain a copy.

13.8. Independent Data Monitoring Committee (IDMC)

An IDMC will be utilized during the conduct of this study. An IDMC is generally assembled when there are significant safety or efficacy issues that warrant external objective medical and/or statistical review in order to protect the ethical and safety interests of subjects and to protect the scientific validity of the study. The schedule of any planned interim analysis and the analysis plan for IDMC review is described in the charter. A copy of the IDMC charter is available from GSK upon request.

14. REFERENCES

Beutler E, Lichtman MA, Coller BS, Kipps TJ. Chapter 119 – Platelet morphology, Biochemistry and Function Williams Hematology. 5th Edition, 1995.

Catella F, Lawson JA, Fitzgerald DJ et al. 1987. Analysis of multiple thromboxane metabolites in plasma and urine. Adv Prostaglandin Thromboxane Leukot Res 17B:611-614.

Cipollone F, Mezzetti A, Porreca E et al. 2002. Association between enhanced soluble CD40L and prothrombotic state in hypercholesterolemia effects of statin therapy. Circulation 106:399-402.

GlaxoSmithKline Document Number ZM2004/00059/00. Investigator Brochure, SB-659032

Green BG, Dalton P, Cowart B et al. 1996. Evaluating the 'Labeled Magnitude Scale' for measuring sensations of taste and smell. *Chemical Senses.* 21:323-334.

Macphee CH, Moores KE, Boyd HF et al. 1999. Lipoprotein associated phospholipase A2, platelet activating factor acetylhydrolase, generates two bioactive products during the oxidation of low density lipoprotein. Use of a novel inhibitor. *Biochem.J.*, 338, 479-487.

McKenzie ME, Gurbel PA, Levine DJ, Serebruany VL. 1999. Clinical Utility of Available Methods for Determining Platelet Function. Cardiology 92:240-247.

Morrow JD, Roberts LJ. Chapter 26 – Lipid-derived Autacoids. Goodman and Gilman's Pharmacological Basis of Therapeutics. 10th Edition, 2001.

Packard CJ, O'Reilly DJ, Caslake MJ ET AL. 2000. Lipoprotein associated phospholipase A2 as an independent predictor of coronary heart disease. New Eng.J.Med., 343, 1148-1155.

Plavix (clopidogrel bisulfate) Prescribing Information. May, 2003.

Ross R. 1999. Atherosclerosis: an inflammatory disease. N Eng J Med, 340, 115-126.

Tew DG, Southan C, Rice SQJ et al. 1996. Purification, properties, sequencing and cloning of a lipoprotein associated serine dependant phospholipase involved in the oxidative modification of low density lipoproteins. Arterioscler.Throm.Vasc.Biol., 16, 591-599.

APPENDICES

Appendix 1: Time and Events Table

Screening: physical examination, medical and medication history, alcohol breath test, tobacco history, vital signs, 12-Lead ECG, clinical laboratory tests and platelet aggregation assessment. See Appendix 2: Clinical Laboratory Tests.

Study Day (Periods 1 and 2)	-2	-1	1	2	3	4	5	6	7	8	9	10	11	12	13	14	15
Admitted to Research Facility	X													X			
Outpatient visits (morning)					X	X	X	X	X	X	X	X	X	X			
Physical examination[1]			X														
Prior/Concomitant medications			X		X	X	X	X	X	X	X	X	X	X			
Baseline signs/symptoms or Adverse Events			X[2]	X[1]	X[1]	X[1]	X[1]	X[1]	X[1]	X[1]	X[1]	X[1]	X[1]	X[1]	X[2]	X[2]	X[4]
Vital Signs[1]			X														
Clinical Lab test (see Appendix 2: Clinical Laboratory Tests)		X[3]															
12-Lead ECG			X[1]														X[4]
Urine Drug Screen[1]			X														
Alcohol Breath Test[1]			X														
Exhaled Carbon Monoxide[1]			X														
SB-659032 administration			X	X	X	X	X	X	X	X	X	X	X	X	X	X	
Blood Sample for PK Analysis[5]			X													X	
Blood Sample for Lp-PLA$_2$ Analysis[5]			X													X	
Blood Sample for Platelet Aggregation[6]		X	X													X	
Blood Sample for Biomarker Analysis (sCD40L)[5]			X													X	
Urine Collection for Biomarker Analysis (U-11-dehydro-TxB2)[7]		X	X													X	
Odor-related questionnaire[8]																X	
Blood Sample for PGx Analysis[9]			X														
Meal Served	X[10]	X[10]	X[11]	X[11]	X[11]	X[11]	X[11]	X[11]	X[11]	X[11]	X[11]	X[11]	X[11]	X[11]	X[10]	X[10]	
Discharge from Research Facility				X													X

Continued

1. Prior to dosing with study medication (physical examination, urine drug screen, alcohol screen and exhaled carbon monoxide may be performed on Day –1)
2. Pre-dose and at 6, 10 and 21 hours post-dose
3. Results must be reported prior to dosing.
4. Prior to discharge from the research facility
5. Prior to dosing (before breakfast) and at the nominal times of 6 and 24 hours following dosing
6. Prior to dosing (before breakfast) and at 6 and 24 hours after dosing on Day 1 and Day 14 and at the equivalent times on Day -1
7. 0-6, 6-12, 12-24 hour urine collection
8. Subjects will be given Questionnaire #1 after the last-dose of each period. If a subject withdrew early from the study, he/she will be given the questionnaire to complete after the last dose.
9. May be collected at any time during the study after the PGx informed consent has been obtained
10. Breakfast 0.5 hour prior to dosing (or equivalent). Lunch and dinner will be served at approximately 6 and 10 hours post-dose (or equivalent) respectively.
11. Breakfast 0.5 hour prior to dosing (or equivalent).

Appendix 1: Time and Events Table (cont)

Minimum Number of Days Post Last Dose	20	21	22
Admitted to Research Facility	X		
Prior/Concomitant medications	X		
Baseline signs/symptoms or Adverse Events	X		
Vital signs	X		
Clinical Lab test (see Appendix 2: Clinical Laboratory Tests)	X[1]		
Urine Drug Screen	X		
Alcohol Breath Test	X		
Exhaled Carbon Monoxide	X		
Blood Sample for Lp-PLA_2 Analysis[2]		X	
Blood Sample for Platelet Aggregation[3]		X	
Blood Sample for Biomarker Analysis (sCD40L)[2]		X	
Urine Collection for Biomarker Analysis (U-11-dehydro-TxB2)[4]		X	
Meal Served	X[5]	X[6]	
Discharge from Research Facility			X

1. Period 2 only (follow up visit)
2. One sample at the time of the first platelet aggregation assessment
3. At the equivalent times to the predose, 6 and 24 hour samples on the dosing days
4. 0-6, 6-12, 12-24 hour urine collection
5. Breakfast, lunch and dinner will be served
6. Breakfast, lunch and dinner will be provided at the equivalent time to 0.5 hour prior to dosing, 6 and 10 hours post-dose respectively

Follow-up: Vital signs, 12-Lead ECG, clinical laboratory and urine tests (see Appendix 2: Clinical Laboratory Tests) and AE assessment.

Appendix 2: Clinical Laboratory Tests

Hematology	
Complete blood count (CBC) with RBC indices and WBC differential	
Platelet count	

Chemistry	
BUN	AST
Creatinine	ALT
Glucose, fasting	GGT
Sodium	Alkaline Phosphatase
Potassium	Total Bilirubin
Chloride	Direct Bilirubin
Total CO_2	Uric Acid
Calcium	Albumin
Total Protein	

Urinalysis
Specific Gravity
pH, glucose, protein, blood and ketones by dipstick
Microscopic examination (if dipstick is positive for blood or protein)

Other tests
Urine Drug Screen (screening, pre-dose Day 1 and post-dose visit)
Urine cotinine (screening only)
HIV, Hepatitis B surface antigen and Hepatitis C antibody (screening only)
Urine creatinine (Days -1, 1, 14 and post-dose visit)
PT, PTT (screening only)

Appendix 3: Values of Potential Clinical Concern

**Hematology**		
Hemoglobin	Males: <12.0 or >18.0 g/dL	Females: <10.5 or >16.1 g/dL
Hematocrit	Males <36.0 or >54.0%	Females: <31.0 or >50.6%
Leukocytes	$>1x10^3/\mu L$ below or $>3x10^3/\mu L$ above the limit of the ref. range	
Platelets	<80 or > 500 K/μL	
**Chemistry**		
Total bilirubin	≥ 1.5 times upper limit of the ref. range	
AST	>2 times upper limit of the ref. range	
ALT	>2 times upper limit of the ref. range	
GGT	>2 times upper limit of the ref. range	
Alk Phosphatase	>1.5 times upper limit of the ref. range	
Creatinine	>1.8 mg/dL	
BUN	>1.5 times upper limit of the ref. range	
Glucose, fasting	<60 or >126 mg/dL	
Uric acid	>11 mg/dL	
Sodium	>5 mEq/L above or below the limits of the ref. range	
Potassium	>0.5 mEq/L above or below the limits of the ref. range	
Calcium	<7.2 or > 12 mg/dL	
Phosphate	>0.8 mg/dL below or 1.0 mg/dL above the limits of the ref. range	
Albumin	>0.5 g/dL above or below the limits of the ref. range	
Total protein	>1.0 g/dL above or below the limits of the ref. range	
**Urinalysis**		
WBC	>15/hpf	
RBC	>15/hpf	
**Vital Signs**		
Heart Rate	Supine: <35 or >100 bpm	Erect: <50 or >110 bpm
Blood Pressure	Systolic >30 mmHg change from baseline in same posture<br>Diastolic >20 mmHg change from baseline in same posture	
**12-Lead Electrocardiogram**		
PR interval	< 120 msec or > 219 msec	
QRS interval	> 119 msec	
QTc interval (Bazett's)	> 470 msec for males<br>> 470 msec for females ≤ 50 years of age<br>> 480 msec for females > 50 years of age	

Appendix 4: PGx

Pharmacogenetic Research

Pharmacogenetics – Background

Pharmacogenetics (PGx) is the study of variability in drug response due to hereditary factors in different populations. There is increasing evidence that an individual's genetic composition (i.e., genotype) may impact the pharmacokinetics (absorption, distribution, metabolism, elimination), pharmacodynamics (relationship between concentrations and pharmacologic effects or the time course of pharmacologic effects) and/or clinical outcome (in terms of efficacy and/or safety and tolerability). Some reported examples of PGx analysis include:

Drug	Disease	Gene	Outcome
Abacavir	HIV [Hetherington, 2002 and Mallal, 2002]	HLA (human leukocyte antigen)	Caucasian males with HLA B57 variant were at increased risk for experiencing hypersensitivity to abacavir
Tranilast	Restenosis prevention following coronary bypass [Danoff, 2004]	UGT1A1	Drug induced hyperbilirubinemia explained by high proportion of affected patients having 7/7 TA repeat genotype, consistent with clinically benign Gilbert's Syndrome
ABT-761	Asthma [Drazen, 1999]	ALOX5	ALOX5 Sp1 promoter genotype (x,x) associated with reduced response to 5-lipoxygenase inhibitor ABT-761

A key component to successful PGx research is the collection of samples during the conduct of clinical studies. Collection of whole blood samples, even when no *a priori* hypothesis has been identified, may enable PGx analysis to be conducted if at any time it appears that there is a potential unexpected or unexplained variation in handling or response to SB-659032.

Pharmacogenetic Research Objectives

If at any time it appears there is potential variability in response in this clinical study or in a series of clinical studies with SB-659032 that may be attributable to genetic variations of subjects, the following objectives may be investigated:

- Relationship between genetic variants and the pharmacokinetics of SB-659032
- Relationship between genetic variants and safety and/or tolerability of SB-659032

Study Population

Any subject who has given informed consent to participate in the clinical study, has met all the entry criteria for the clinical study, and receives investigational product may take part in the PGx research. Any subject who has received an allogeneic bone marrow transplant must be excluded from the PGx research.

Subject participation in the PGx research is voluntary and refusal to participate will not indicate withdrawal from the clinical study. Refusal to participate will involve no penalty or loss of benefits to which the subject would otherwise be entitled.

Study Assessments and Procedures

In addition to any blood samples take for the clinical study, a whole blood sample (~10ml) will be collected for the PGx research using a tube containing EDTA. The PGx sample is labeled (or "coded") with a study specific number that can be traced or linked back to the subject by the investigator or site staff. Coded samples do not carry personal identifiers (such as name or social security number). The blood sample will be taken on a single occasion unless a duplicate sample is required due to inability to utilize the original sample. It is recommended that the blood sample be taken at the first opportunity after a subject has been randomized and provided informed consent for PGx research, but may be taken at any time while the subject is participating in the clinical study.

If deoxyribonucleic acid (DNA) is extracted from the blood sample, the DNA may be subjected to sample quality control analysis. This analysis will involve the genotyping of several genetic markers to confirm the integrity of individual samples. If inconsistencies are noted in the analysis, then those samples may be destroyed.

Subject Withdrawal from Study

If a subject who has consented to participate in PGx research withdraws from the clinical study for any reason other than lost to follow-up, the subject will be given the following options concerning the PGx sample, if already collected:

- PGx research continues per the subject's consent; or,
- Any remaining sample is destroyed

If a subject withdraws consent from the PGx research or requests sample destruction, the investigator must complete the appropriate documentation to request sample destruction within the timeframe specified by GSK and maintain the documentation in the site study records. In either case, GSK will only keep study information collected/generated up to that point.

Screen and Baseline Failures

If a blood sample for PGx research has been collected and it is determined that the subject does not meet the entry criteria for participation in the clinical study, then the investigator must complete the appropriate documentation to request sample destruction

within the timeframe specified by GSK and maintain the documentation in the site study records.

Pharmacogenetics Analyses

The need to conduct PGx analysis may be identified after a study (or set of studies) of SB-659032 has been completed and the study data reviewed. For this reason, samples may be kept for up to 15 years after the last subject completes the study or GSK may destroy the samples sooner. In special cases, the samples may not be studied. This might happen if there are not enough subjects, if the study is stopped for other reasons, or if no questions are raised about how people respond SB-659032.

Generally GSK will utilize two approaches to explore genetic variation in drug response.

1. Specific sections of DNA may be selected from areas of the genome (e.g., candidate genes) known to encode the drug target, drug metabolizing enzymes, areas associated with mechanisms underlying adverse events, and those linked to study disease and, thus, linked to drug response.
2. By evaluating large numbers of polymorphic markers (e.g., single nucleotide polymorphisms or SNPs) throughout the genome, sets of markers may be identified that correspond to differential drug response.

Hardy-Weinberg Equilibrium Testing

The genotypic frequencies of each polymorphism will be evaluated for conformity to those expected under normal conditions by employing Hardy-Weinberg Equilibrium testing.

Comparison of Demographic and Baseline Characteristics by Genotype

Differences in baseline clinical characteristics and potential contributing covariates may be summarized and compared among genotype (or haplotype) subgroups.

Evaluation of Genotypic Effects

Analyses may be carried out to evaluate the degree of association between subject genotype (or haplotype) and selected parameters (e.g., pharmacokinetics, efficacy and safety). Where such genotypic tests are inappropriate (for example, where the number of marker genotypes is too large and/or the frequency of individual genotypes too small), allelic tests may be conducted. Allelic tests evaluate whether the frequency of each marker allele is the same in responders and non-responders.

Evaluation of Treatment by Genotype and Gene-Gene Interaction

In addition to evaluating the main effects of the genotypes (haplotypes or alleles) on the selected parameters, the possibility of a treatment group by genotype (haplotype or allele) interaction will also be explored. If appropriate, the joint effects of multiple markers (gene-gene interactions) may also be evaluated.

Linkage Disequilibrium

For pairs of polymorphisms, the degree to which alleles from the two sites are correlated (linkage disequilibrium) may also be evaluated. If the genotypes at two polymorphic sites within a gene are shown to be statistically associated with a response to investigational product, the degree of linkage disequilibrium will aid interpretation in that it will indicate the extent to which the two sites are exerting independent effects.

Multiple Comparisons and Multiplicity

To the extent that multiple markers are evaluated (especially in the case of a genome scan for association), an adjustment to observed p-values may be made to limit erroneous conclusions due to multiple tests.

Power and Sample Size Considerations

The ability to detect differential drug response among genotypes at a polymorphic site depends on the total number of subjects genotyped and the frequency distribution of the different genotypes. Consequently, genotyping analyses are plausible for those polymorphic sites where the number of subjects comprising the genotypic groups is sufficiently large; however, these frequencies will not be known until sufficient samples have been collected and genotyping is complete.

Estimates of sample sizes required to demonstrate genotype effects vary considerably, depending on the assumptions made about allele frequency, genetic effect size, and mechanism of inheritance [Cardon, 2000]. In the work by Palmer and Cookson [Palmer, 2001], which assumed a genotype relative risk of 1.5, it was estimated that more than 300 cases and 600 controls would be needed to conduct a genetic association analysis. In contrast, McCarthy and Hilfiker [McCarthy, 2000] showed that with a genotype relative risk of 2.16 and a relatively commonly occurring genotype, only 30 cases and 30 controls would be needed to demonstrate an association.

Published PGx examples include abacavir hypersensitivity reaction [Hetherington, 2002 and Mallal, 2002] and tranilast induced hyperbilirubinemia [Danoff, 2004] where genetic markers have been found to significantly associate with hypersensitivity reaction (abacavir) and hyperbilirubinemia (tranilast). These examples show that small sample sizes typically encountered in Phase I and Phase II studies may be sufficient to identify clinically relevant genetic associations.

Informed Consent

Subjects who do not wish to participate in the PGx research may still participate in the clinical study. PGx informed consent must be obtained prior to any blood being taken for PGx research.

References

Cardon LR, Idury RM, Harris TJR, Witte JS, Elston RC. Testing drug response in the presence of genetic information: sampling issues for clinical trials. *Pharmacogenetics*. 2000; 10:503-10.

Danoff TM, Campbell DA, McCarthy LC, Lewis KF, Repasch MH, Saunders AM, Spurr NK, Purvis IJ, Roses AD, Xu CF. A Gilbert's syndrome UGT1A1 variant confers susceptibility to tranilast-induced hyperbilirubinemia. Pharmacogenomics J 2004;4:49-53.

Drazen JM, Yandava CN, Dube L, Szcerback N, Hippensteel R, Pillari A, Israel E, Schork N, Silverman ES, Katz DA, Drajesk J. Pharmacogenetic association between ALOX5 promoter genotype and the response to anti-asthma treatment. *Nature Genet*. 1999; 22:168-70.

Hetherington S, Hughes AR, Mosteller M, Shortino D, Baker KL, Spreen W, Lai E, Davies K, Handley A, Dow DJ, Fling ME, Stocum M, Bowman C, Thurmond LM, Roses AD. Genetic variations in HLA-B region and hypersensitivity reactions to abacavir. *Lancet*. 2002; 359:1121-2.

Mallal S, Nolan D, Witt C, Masel G, Martin AM, Moore C, Sayer D, Castley A, Mamotte C, Maxwell D, James I. Association between presence of HLA-B*5701, HLA-DR7, and HLA-DQ3 and hypersensitivity to HIV-1 reverse-transcriptase inhibitor abacavir. *Lancet*. 2002; 359:727-32.

McCarthy JJ, Hilfiker R. The use of single-nucleotide polymorphism maps in pharmacogenomics. *Nat Biotechnol*. 2000; 18:505-8.

Palmer LJ, Cookson WO. Using single nucleotide polymorphisms as a means to understanding the pathophysiology of asthma. *Respir Res*. 2001; 2:102-12.

Division: Worldwide Development
Retention Category: GRS019
Information Type: Clinical Pharmacology Protocol

**Title:**	A Double Blind, Placebo controlled, Parallel Study to Evaluate Effects of Repeat Doses of Rilapladib on Platelet Aggregation in Healthy Male Volunteers

Compound Number: SB-659032

Effective Date: 01-Sep-2006

Description:

SB-659032 (rilapladib) is a selective and orally active inhibitor of lipoprotein-associated phospholipase A_2 (Lp-PLA_2) that is being developed for the treatment of atherosclerosis. A previous study (LP2104623) resulted in data that suggested an enhanced collagen-induced platelet aggregation 21 days after the last dose of rilapladib. The study's crossover design, the higher than expected variability of the collagen assay, a fluctuation of the placebo baseline, and a multiplicity issue with multiple endpoints (2 agonists) and time points limited the ability to properly interpret the results. The clinical relevance of the results is unknown and there is a 5-10% chance it was a random finding. This current study will help investigate this finding further with design changes aimed to address the issues in the previous study. This will be a double blind, 14-day repeat dose, randomized, placebo-controlled, parallel study. Platelet aggregation assessments will occur on Days -1, 1, 14 and 35 (21 days post-last dose).

Subject: SB-659032, platelet aggregation, rilapladib

Author(s):

Harter, Angela K	Clinical Sciences and Study Operations
Haws, Jr., Thomas F	Clinical Sciences and Study Operations
Leonov, Sergei	Research Statistics Unit
Magee, Mindy	CPK/M&S
Roger, James H	Research Statistics Unit
Shaddinger, Bonnie	Discovery Medicine
Xu, Yanmei	Clinical Pharmacology Statistics and Programming

Copyright 2006 the GlaxoSmithKline group of companies. All rights reserved. Unauthorised copying or use of this information is prohibited.

CONFIDENTIAL ZM2006/00132/00
LP2108364

SPONSOR SIGNATORY:

Anthony G. Johnson, M.D.	1st Sept., 2006
Anthony G. Johnson, M.D.<br>VP, CVU Discovery Medicine	**Date**

2

SPONSOR INFORMATION PAGE

Clinical Study Identifier: LP2108364

Sponsor Contact Information:

GlaxoSmithKline
2301 Renaissance Boulevard
Building #510
Post Office Box 61540
King of Prussia, Pennsylvania 19406
Telephone number: +1 610 787-7000
Sponsor Serious Adverse Events (SAE) Contact Information:

Primary Medical Monitor:

John Lepore	
Telephone Number	610 270 6414
After hours Contact Number	610 804 8120
Fax Number	610 270 5250

Back-up Medical Monitor:

Dennis Sprecher, M.D.	
Telephone number	610 270 6007
After hours telephone number	267 879 2599
Fax number	610 270 4114

INVESTIGATOR PROTOCOL AGREEMENT PAGE

I confirm agreement to conduct the study in compliance with the protocol.

Investigator Name: ______________________________

____ ____________________

Investigator Signature Date

TABLE OF CONTENTS

	Page
ABBREVIATIONS	8
PROTOCOL SUMMARY	10
1. INTRODUCTION	15
1.1. Background	15
1.2. Rilapladib (SB-659032)	15
1.2.1. Rilapladib Clinical Pharmacology Studies	16
1.3. Rationale	17
2. OBJECTIVE(S)	18
2.1. Primary	18
2.2. Secondary	18
2.3. Exploratory	19
3. ENDPOINT(S)	19
3.1. Primary	19
3.2. Secondary	19
3.3. Exploratory	19
4. STUDY DESIGN	20
5. STUDY POPULATION	20
5.1. Number of Subjects	20
5.2. Eligibility Criteria need to be reviewed	21
5.2.1. Inclusion Criteria	21
5.2.2. Exclusion Criteria	21
5.2.3. Other Eligibility Criteria Considerations	23
6. STUDY ASSESSMENTS AND PROCEDURES	23
6.1. Demographic and Baseline Assessments	23
6.1.1. Demography	23
6.1.2. Medical History/medication	23
6.2. Safety	24
6.2.1. Physical Exam	24
6.2.2. Vital Signs	24
6.2.3. 12-Lead Electrocardiogram (ECG) Monitoring	24
6.2.4. Clinical Laboratory Measurement	24
6.2.5. Pregnancy	25

6.3. Deviations from the Time and Events Schedule 25
6.4. Pharmacokinetics 26
6.4.1. Sample Collection and Preparation 26
6.4.2. Assay Methodology 26
6.4.3. Planned Pharmacokinetic Evaluation 26
6.5. Pharmacodynamics 26
6.5.1. Sample Collection and Preparation 26
6.5.2. Assay Methodology 27
6.5.3. Planned Pharmacodynamic Evaluation 28
6.6. Pharmacogenetics 28

7. LIFESTYLE AND/OR DIETARY RESTRICTIONS 28
7.1. Meals and Dietary Restrictions 28
7.2. Caffeine and Alcohol 30
7.3. Activity 30

8. INVESTIGATIONAL PRODUCT(S) 30
8.1. Description of Investigational Product 30
8.2. Dosage and Administration 30
8.3. Dose Rationale 30
8.4. Blinding 31
8.5. Treatment Assignment 31
8.6. Packaging and Labeling 32
8.7. Preparation 32
8.8. Handling and Storage 32
8.9. Product Accountability 32
8.10. Assessment of Compliance 32
8.11. Treatment of Investigational Product Overdose 32
8.12. Occupational Safety 33

9. CONCOMITANT MEDICATIONS AND NON-DRUG THERAPIES 33
9.1. Permitted Medications 33
9.2. Prohibited Medications 33
9.3. Non-Drug Therapies 33

10. SUBJECT COMPLETION AND WITHDRAWAL 33
10.1. Subject Completion 33
10.2. Subject Withdrawal 34
10.2.1. Subject Withdrawal from Study 34
10.2.2. Subject Withdrawal from Investigational Product 34

10.3. Screen and Baseline Failures 34

11. ADVERSE EVENTS (AE) AND SERIOUS ADVERSE EVENTS (SAE) . . 34
11.1. Definition of an AE 34
11.2. Definition of a SAE 35
11.2.1. Clinical Laboratory Abnormalities and Other Abnormal Assessments as AEs and SAEs 35
11.3. Time Period, and Frequency of Detecting AEs and SAEs 37
11.4. Prompt Reporting of SAEs to GSK 38
11.4.1. Timeframes for Submitting SAE Reports to GSK 38
11.5. AE and SAE Documentation and Follow-up Procedures 38

12. DATA ANALYSIS AND STATISTICAL CONSIDERATIONS 38
12.1. Hypotheses 38
12.2. Study Design Considerations 39
12.2.1. Sample Size Assumptions 39
12.2.2. Sample Size Sensitivity 41
12.2.3. Sample Size Re-estimation 41

13. STUDY CONDUCT CONSIDERATIONS 45
13.1. Regulatory and Ethical Considerations, Including the Informed Consent Process 45
13.2. Quality Control (Study Monitoring) 45
13.3. Quality Assurance 46
13.4. Study and Site Closure 46
13.5. Records Retention 46
13.6. Provision of Study Results and Information to Investigators 47
13.7. Data Management 47

14. REFERENCES 48

Appendices 49
Appendix 1: Time and Events Table 50
Appendix 2: Framingham Point Score 52
Appendix 3: Pharmacogenetic Research 53

ABBREVIATIONS

2,3-diFBCl	2,3-difluorobenzylchloride
ADP	Adenosine Diphosphate
AE	Adverse Event
ANOVA	Analysis of Variance
AUC	Area under the plasma concentration vs time curve
ALT	Alanine amino transferase
AST	Aspartate amino transferase
bTG	β-thromboglobulin
BUN	Blood Urea Nitrogen
CI	confidence interval
CBC	Complete blood count
CIB/IB	Clinical Investigator's Brochure/Investigator's Brochure
CPK	Creatinine phosphokinase
Cmax	Maximum drug concentration
Cmin	Minimum drug concentration
CRF	Case report form (electronic or hard copy)
CRU	Clinical Research Unit
CSR	Clinical study report
CV	Cardiovascular
CYP3A4	cytochrome P450 enzyme 3A4
DNA	deoxyribonucleic acid
EC	enteric coated
ECG	Electrocardiogram
FDA	Food and Drug Administration
GCP	Good Clinical Practices
GGT	Gamma glutamyltransferase
GSK	GlaxoSmithKline
HIV	Human Immunodeficiency Virus
IEC	Independent Ethics Committee
IND	Investigational New Drug application
IRB	Institutional Review Board
IUD	Intrauterine device
LDL	Low density lipoprotein
LLQ	Lower limit of quantification
Lp-PLA2	Lipoprotein-associated phospholipase A2
Lyso-PC	Lysophosphatidylcholine
M	Molar
MSDS	Material Safety Data Sheet
NOAEL	No Adverse Effect Level
PD	Pharmacodynamic
PAF-AH	platelet activating factor acetylhydrolase
PLA2	Phospholipase A2
PGx	Pharmacogenetics
PK	Pharmacokinetic
ppm	Parts per million
PRP	Platelet rich plasma

QC	Quality Control
RBC	Red blood cell
SAE	Serious adverse event
sCD40L	Soluble CD40 ligand
sd	Standard deviation
SNP	Single Nucleotide Polymorphism
TxB2	Thromboxane B2
t½	Terminal phase elimination half-life
Tmax	Time of maximal plasma concentration
UDS	Urine drug screen
ULN	Upper limit of normal

PROTOCOL SUMMARY

Rationale

SB-659032 (rilapladib) is a selective and orally active inhibitor of lipoprotein-associated phospholipase A_2 [Lp-PLA_2] that is being developed for the treatment of atherosclerosis. Lp-PLA_2, also known as platelet activating factor acetylhydrolase (PAF-AH), is one of the enzymes responsible for the hydrolysis of PAF [platelet activating factor] into biologically inactive lyso-PAF. PAF is synthesized by a variety of cell types including platelets, neutrophils, monocytes, mast cells and eosinophils. In platelets, PAF promotes aggregation by stimulating a G protein-linked cell surface receptor. Although PAF is a weak platelet agonist by itself, its presence can lower the required dose of other agonists to induce platelet activation.

Rilapladib will be studied in patients with documented evidence of coronary heart disease including patients with acute coronary syndrome where the use of antiplatelet agents such as aspirin and/or clopidogrel is considered part of standard care. It is theoretically possible that an Lp-PLA_2 inhibitor may result in accumulation of PAF, promoting platelet aggregation and hence negate the beneficial effects of anti-platelet agents. In a clinical study (study LP2102487) in which healthy subjects were administered a single 250 mg dose of the compound rilapladib, preliminary data [data on file] suggests that 11 out of 14 subjects had enhanced platelet aggregation (>15% change from baseline) at 6 hours post-dose following the addition of 0.1 or 0.2 mcg/mL of the agonist collagen. In addition, 3 out of the 14 subjects displayed enhanced platelet aggregation at 24 hours post-dose and 8 out of the 14 subjects displayed enhanced platelet aggregation at the 14 day follow-up visit. In the absence of a placebo control in this trial, the clinical significance of these results is unclear, highlighting the need to complete a placebo-controlled study.

Study LP2104623 was a randomized, double blind, placebo-controlled study to evaluate effects of repeat doses of 250 mg rilapladib on platelet aggregation in healthy male volunteers. Twenty-six healthy adult male subjects were enrolled at one site. Each subject was to participate in two study sessions and was randomized to receive each of the following treatments: 1) 250 mg free base SB-650932 once daily for 14 days or 2) rilapladib matched placebo once daily for 14 days. There was a 21 day washout period at the end of each dosing session. Five subjects withdrew from the study. Twenty-one subjects completed the study, having received both treatments in randomized order. There was no statistically significant effect on platelet aggregation in response to collagen or ADP during dosing of subjects with rilapladib (ie, lower bound of the 90% CI < 0%) at all timepoints on Day 1 and at steady state (Day 14). All corresponding point estimates were <15%. However, at the end of the 21 days washout period (following the last dose of rilapladib), the data suggested an enhanced collagen-induced platelet aggregation (i.e. upper bound of the 90% CI > 15% and lower bound > 0%), which was not seen with ADP. At this time, there was no detectable plasma concentration of rilapladib or its major metabolite SB-664601. However, there may still be minor inhibition of Lp-PLA_2 activity (approximately 16±9.4%) during the off-drug period.

Based upon the biology of PAF, which is stored in platelet granules and has a very short half life, the enhanced platelet aggregation seen at the end of the 21 day washout period

is unlikely mediated through PAF and therefore is considered to be unrelated to the primary mechanism action of a Lp-PLA_2 inhibitor such as rilapladib.

Other potential etiologies for the enhanced platelet aggregation observed at the end of the 21 day washout period include, (1) the effects of an unidentified metabolite, (2) an immune response, or (3) a defect in platelet maturation. However, these are equally difficult to explain given the typical immune responses are manifested within 4-10 days, and the fact that platelet life span is only 7 to 10 days.

Because data at the end of the wash-out of the first period (Day 21) served as the baseline for the period two analysis, subjects' data in period 1 may have influenced their data obtained from period 2. Therefore, the study's crossover design, in addition to other factors such as the higher than expected variability of the collagen assay, *a priori* collagen concentration selection rules, and a fluctuation of the placebo time course data, may have limited the ability to properly interpret the collagen effect on platelets at the end of the 21 day washout period. The clinical relevance of the degree of collagen platelet aggregation seen at the end of the wash out period is unknown and there is a 5-10% chance it was a random finding.

In the context of uncertainties related to the interpretation of these prior platelet aggregation studies with rilapladib, this parallel placebo controlled study will further investigate the potential for platelet aggregation during 14 days repeat oral dosing with rilapladib and after stopping the drug for 21 days using a more comprehensive collagen-induced platelet aggregation stimulus package.

Objective(s)

Primary

1. To demonstrate a lack of effect of the 14 days of repeat oral doses of 250 mg of rilapladib on platelet aggregation as compared to placebo on Day 35 (or 21 days post last dose)

Secondary

1. To estimate the effect of single and repeat oral doses of 250 mg of rilapladib on platelet aggregation as compared to placebo on Day 1 and at steady state (Day 14)
2. To characterize the degree of inhibition of plasma Lp-PLA_2 on Days 1 and 14 and during the off drug period following 14 days of repeat oral dosing with 250mg of rilapladib
3. To assess the safety and tolerability of 14 days of repeat oral doses of 250 mg of rilapladib
4. To measure concentrations of rilapladib, its major metabolite, SB-664601, and other potential metabolites with 14 days of repeat oral dosing with 250mg of rilapladib

Exploratory

1. To evaluate the effects of 14 days repeat oral dosing with 250mg of rilapladib on platelet maturation as measured by platelet count and mean platelet volume (MPV) on Days 1 and 14 (during treatment) and 35 (or 21 days post last dose)
2. To evaluate whether isolated platelets derived from rilapladib treated subjects behave aberrantly in normal plasma as well as to determine the effect of plasma derived from rilapladib treated subjects on untreated platelets on Day 34 (+/- 4 days), if feasible
3. To evaluate the effects of rilapladib on the initial rate of aggregation as determined by the slope of the individual aggregometer tracing after shape change
4. To evaluate the effects of rilapladib on immune responses of platelets, as data permit

Endpoint(s)

Primary

1. Collagen EC50 values (concentration which induces 50% of maximal aggregation) on Day 35 (or 21 days post last dose) as determine by optical aggregometry

Secondary

1. Collagen EC50 values (concentration which induces 50% of maximal aggregation) on Day 1 and Day 14 as determined by optical aggregometry
2. Plasma Lp-PLA_2 activity, expressed in terms of percent inhibition relative to baseline, on Days 1, 14 and 35 (or 21 days post last dose)
3. Clinical safety data from spontaneous adverse event reporting, 12-Lead ECG, vital signs, nursing/physician observation and safety laboratory tests
4. Concentrations of rilapladib, its major metabolite, SB-664601, and other potential metabolites, on Days 1, 14 and 35 (or 21 days post last dose)

Exploratory

1. Platelet maturation as measured by platelet count and MPV during treatment (Days 1 and 14) and off drug period (Day 35)
2. Collagen EC50 values (concentration which induces 50% of maximal aggregation) for normal platelets mixed into the post-treatment plasma, and for the post-treatment platelets mixed into normal plasma, on Day 34 (+/- 4 days) only, if feasible
3. Collagen concentration which induces 50% of the maximal slope change on Day 1, 14 and 35
4. Platelet surface immunoglobulin measured by RIA or flow cytometry, as data permits

Study Design

This will be a double blind, repeat dose, randomized, placebo-controlled, parallel study. Each subject will be randomized to either rilapladib or placebo and will be dosed for 14 days (in-house from Day -2 through Day 1; outpatient witnessed dosing Days 2-13; in house Day 13-14; discharge on Day 14). Platelet aggregation measurements will occur on Day -1, Day 1, Day 14 and Day 35 (relative to the start of dosing on Day 1). On Day 34 (+/- 4 days) a subset of subjects may have blood collected to be used for ex-vivo evaluation of the plasma and platelets on platelet aggregation when combined with normal (untreated) platelets and normal (untreated) plasma. The entire study will consist of a minimum of ~4 or 5 overnight stays and ~12 outpatient visits.

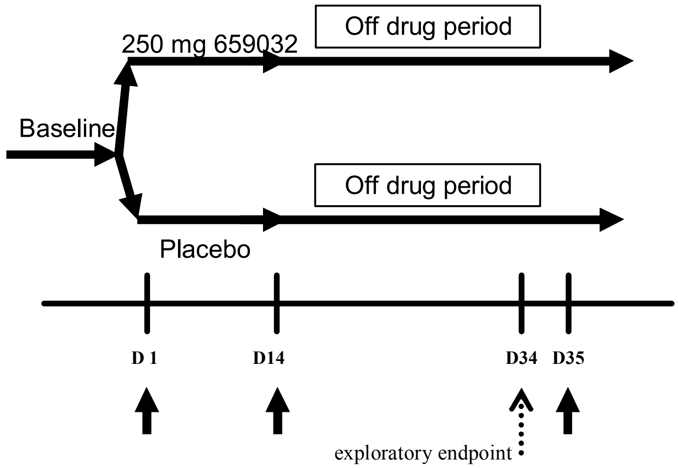

Regimen	Description
A	250 mg rilapladib QD for 14 days
B	Rilapladib matched placebo QD for 14 days

Each subject's participation in the study will be approximately 12 weeks from screening to follow-up.

Study Population

Approximately 66 subjects are targeted for enrollment. Following completion of the first cohort (~28 subjects) there will be a sample size re-estimation to determine if the planned sample size is sufficient or if the sample size should be adjusted. Eligible subjects will be healthy adult males between 18 and 55 years of age who have a 10 year risk of cardiovascular events of <10% (based on Framingham Point Score).

Study Assessments

Subjects will report to the research facility 2 days prior to the start of dosing and will remain in the facility until the afternoon of Day 1. On the morning of Day 1, subjects will be randomized to receive either 250 mg of rilapladib or rilapladib matched-placebo for 14 days. Subjects will be dosed at the research facility as outpatients on Days 2 to 13.

On Day 13, subjects will be re-admitted to the facility and will be discharged on Day 14. Blood samples for platelet aggregation will be collected predose and 6 hrs postdose on Day 1 and Day 14). Subjects will return for additional platelet aggregation measurements during the off drug period on Day 35 (20 and 21 days following the last dose). They will be admitted to the unit the night before and blood samples will be obtained the next morning at the time subjects were typically dosed and again 6 hours later. Blood samples will also be collected at the same timepoints listed above on Days 1, 14 and 35 to measure plasma concentrations of rilapladib, SB-664601 (major metabolite) and cold metabolites; inhibition of Lp-PLA_2 relative to baseline; platelet count; and MPV. A subset of the subjects will also have an additional blood draw on Day 34 (+/- 4 days) for ex-vivo evaluation of the subject plasma and platelets on platelet aggregation when combined with normal (untreated) platelets and normal (untreated) plasma from a different group of subjects. Assessment of tolerability to study medication will be made during the study by heart rate and blood pressure measurements, 12-Lead ECGs, laboratory safety tests, adverse event questioning and nursing/physician observation.

1. INTRODUCTION

1.1. Background

Cardiovascular (CV) disease accounts for approximately 50% of all deaths (from myocardial infarction and stroke) in both developed and developing countries. Atherosclerosis is the fundamental pathology behind most CV deaths and is now universally acknowledged as an inflammatory disease and not one that simply results from the arterial accumulation of lipids. This specialized inflammatory disease of the intima is characterized by leukocyte (monocytes and T-lymphocytes) recruitment and accumulation having a major role in both early and continuing aspects of its pathogenesis. Indeed, the histopathological examination of plaques that have been identified as culprit lesions causing a myocardial infarction more often than not occupy less than 50% of the vessel lumen cross sectional area, have a soft lipid core, and a thin friable cap with a high concentration of macrophages in the so called "shoulder region." Thus, macrophage driven chronic inflammation appears to represent an important destabilizing process in the arterial lesion [Ross, 1999].

The sub-endothelial oxidation of low density lipoprotein (LDL) is viewed as a highly significant biological process that both initiates and accelerates arterial lesion development [Ross, 1999]. One of the earliest events in LDL oxidation is the hydrolysis of oxidatively modified phosphatidylcholine, generating substantial quantities of lysophosphatidylcholine (lyso-PC) and oxidized fatty acids. This hydrolysis is mediated by lipoprotein-associated phospholipase A_2 (Lp-PLA$_2$), an enzyme that is found associated predominantly with LDL in human plasma [Tew, 1996]. A significant volume of evidence has accumulated in favor of both by-products, especially lyso-PC, being proinflammatory and proatherogenic mediators [Macphee, 1999]. Thus, preventing the generation of these mediators through inhibition of Lp-PLA$_2$ should retard atherosclerosis by interfering with inflammatory cell localization, activation, pro-inflammatory function and death. Consistent with this notion is the recent observation that plasma levels of Lp-PLA$_2$ represent an independent predictor of coronary heart disease [Packard, 2000].

1.2. Rilapladib (SB-659032)

Rilapladib may prevent the accumulation of lyso-PC and other pro-inflammatory lipids generated during the oxidation of LDL. Lyso-PC has several pro-atherogenic activities ascribed to it, including monocyte chemotaxis and induction of endothelial dysfunction, both of which facilitate monocyte-derived macrophage accumulation within the artery wall. An inhibitor of Lp-PLA$_2$ such as rilapladib is predicted to inhibit intimal macrophage accumulation and therefore retard and stabilize the developing atherosclerotic plaque.

Rilapladib represents the third inhibitor of Lp-PLA$_2$ to be evaluated in the clinic, the first two having been SB-435495 and SB-480848 (darapladib). Rilapladib is a back up compound of darapladib that has been in clinical development since 2001.

$Lp-PLA_2$ is often referred to in the literature as platelet-activating factor acetyl hydrolase (PAF-AH) since it was first characterized as a plasma enzyme activity that hydrolyzed exogenously added PAF. $Lp-PLA_2$ and PAF-AH are different names for the same enzyme but $Lp-PLA_2$ is a more general term, because this enzyme can hydrolyze a broad range of substrates in addition to PAF. $Lp-PLA_2$ may be a primary mechanism of PAF removal *in vivo* and a deficiency in its enzymatic activity could potentially enhance PAF-mediated responses and exacerbate asthmatic conditions. Hence, patients with asthma are excluded in clinical trials of rilapladib until an appropriate study in an asthmatic population with an $Lp-PLA_2$ inhibitor has been performed.

Rilapladib absorbs light in the waveband of concern (290-700 nM) for potential phototoxicity, and (based on rat data) shows an association with melanin in skin and eye. Although, the risk associated with this observation is likely to be very low. Subjects should avoid direct sunlight during the study, from the administration of the first dose until 3 days after the last dose. Sunblock (SPF ≥ 30) should be applied to areas of skin exposed to direct sunlight for more than 15 minutes.

1.2.1. Rilapladib Clinical Pharmacology Studies

As of August 2006, six clinical pharmacology studies with rilapladib have been initiated. Single, oral doses up to 250 mg of non-enteric coated rilapladib were evaluated in the following studies:

- 001 (single ascending dose)
- 002 (Part II, food effect)
- LP2102487 (single dose, platelet aggregation)

Repeat, oral doses of up to 400 mg of non-enteric coated rilapladib were evaluated in studies:

- 002 (Part I, single and repeat ascending dose)
- 004 (age/gender)
- LP2104623 (repeat dose, platelet aggregation)

And study 902 (odor study) assessed repeat, oral doses of 400 mg of enteric coated rilapladib.

As of August 2006, approximately 195 healthy (181 male, 15 female) subjects have been exposed to at least 1 oral dose of rilapladib. Of these, approximately 134 (124 male, 10 female) subjects have been exposed to repeat, oral doses of rilapladib ranging from 20-400 mg UID and for a duration of up to 14 days. Exposure appeared to increase less than dose proportionally following single and repeat doses of non-enteric coated rilapladib. The half-life ranged from 20 to 70 hours in healthy subjects. Accumulation following repeat dosing (25-100%) was less than that predicted from single dose pharmacokinetic data (150-230%) and steady-state concentrations of rilapladib appeared to be achieved following 14 days of once daily dosing. Exposure to the pharmacologically active metabolite, SB-664601, was on average 12% of the parent exposure in man.

Please refer to the CIB for detailed information on these Phase I studies [GlaxoSmithKline Document Number ZM2004/00059/01].

1.3. Rationale

Rilapladib is a selective and orally active inhibitor of lipoprotein-associated phospholipase A_2 [Lp-PLA_2] that is being developed for the treatment of atherosclerosis. Lp-PLA_2, also known as platelet activating factor acetylhydrolase (PAF-AH), is one of the enzymes responsible for the hydrolysis of PAF [platelet activating factor] into biologically inactive lyso-PAF. PAF is synthesized by a variety of cell types including platelets, neutrophils, monocytes, mast cells and eosinophils. In platelets, PAF promotes aggregation by stimulating a G protein-linked cell surface receptor. Although PAF is a weak platelet agonist by itself, its presence can lower the required dose of other agonists to induce platelet activation [Morrow, 2001].

Rilapladib will be studied in patients with documented evidence of coronary heart disease including patients with acute coronary syndrome where the use of antiplatelet agents such as aspirin and/or clopidogrel is considered part of standard care. It is theoretically possible that an Lp-PLA_2 inhibitor may result in accumulation of PAF, promoting platelet aggregation and hence negate the beneficial effects of anti-platelet agents. In a clinical study (study LP2102487) in which healthy subjects were administered a single 250 mg dose of the compound rilapladib, preliminary data [data on file] suggests that 11 out of 14 subjects had enhanced platelet aggregation (>15% change from baseline) at 6 hours post-dose following the addition of 0.1 or 0.2 mcg/mL of the agonist collagen. In addition, 3 out of the 14 subjects displayed enhanced platelet aggregation at 24 hours post-dose and 8 out of the 14 subjects displayed enhanced platelet aggregation at the 14 day follow-up visit. In the absence of a placebo control in this trial, the clinical significance of these results is unclear, highlighting the need to complete a placebo-controlled study.

Study LP2104623 was a randomized, double blind, placebo-controlled study to evaluate effects of repeat doses of 250 mg rilapladib on platelet aggregation in healthy male volunteers. Twenty-six healthy adult male subjects were enrolled at one site. Each subject was to participate in two study sessions and was randomized to receive each of the following treatments: 1) 250 mg free base SB-650932 once daily for 14 days or 2) rilapladib matched placebo once daily for 14 days. There was a 21 day washout period at the end of each dosing session. Five subjects withdrew from the study. Twenty-one subjects completed the study, having received both treatments in randomized order. There was no statistically significant effect on platelet aggregation in response to collagen or ADP during dosing of subjects with rilapladib (ie, lower bound of the 90% CI < 0%) at all timepoints on Day 1 and at steady state (Day 14). All corresponding point estimates were <15%. However, at the end of the 21 days washout period (following the last dose of rilapladib), the data suggested an enhanced collagen-induced platelet aggregation (i.e. upper bound of the 90% CI > 15% and lower bound > 0%), which was not seen with ADP. At this time, there was no detectable plasma concentration of rilapladib or its major metabolite SB-664601. However, there may still be minor inhibition of Lp-PLA_2 activity (approximately 16±9.4%) during the off-drug period.

Based upon the biology of PAF, which is stored in platelet granules and has a very short half life [Stafforini, 1997], the enhanced platelet aggregation seen at the end of the 21 day washout period is unlikely mediated through PAF and therefore is considered to be unrelated to the primary mechanism action of a Lp-PLA_2 inhibitor such as rilapladib.

Other potential etiologies for the enhanced platelet aggregation observed at the end of the 21 day washout period include, (1) the effects of an unidentified metabolite, (2) an immune response, or (3) a defect in platelet maturation. However, these are equally difficult to explain given the typical immune responses are manifested within 4-10 days, and the fact that platelet life span is only 7 to 10 days.

Because data at the end of the wash-out of the first period (Day 21) served as the baseline for the period two analysis, subjects' data in period 1 may have influenced their data obtained from period 2. Therefore, the study's crossover design, in addition to other factors such as the higher than expected variability of the collagen assay, *a priori* collagen concentration selection rules, and a fluctuation of the placebo time course data, may have limited the ability to properly interpret the collagen effect on platelets at the end of the 21 day washout period. The clinical relevance of the degree of collagen platelet aggregation seen at the end of the wash out period is unknown and there is a 5-10% chance it was a random finding.

In the context of uncertainties related to the interpretation of these prior platelet aggregation studies with rilapladib, this parallel placebo controlled study will further investigate the potential for platelet aggregation during 14 days repeat oral dosing with rilapladib and after stopping the drug for 21 days using a more comprehensive collagen-induced platelet aggregation stimulus package.

2. OBJECTIVE(S)

2.1. Primary

1. To demonstrate a lack of effect of the 14 days of repeat oral doses of 250 mg of rilapladib on platelet aggregation as compared to placebo on Day 35 (or 21 days post last dose)

2.2. Secondary

1. To estimate the effect of single and repeat oral doses of 250 mg of rilapladib on platelet aggregation as compared to placebo on Day 1 and at steady state (Day 14)
2. To characterize the degree of inhibition of plasma Lp-PLA2 on Days 1 and 14 and during the off drug period following 14 days of repeat oral dosing with 250mg of rilapladib
3. To assess the safety and tolerability of 14 days of repeat oral doses of 250 mg of rilapladib
4. To measure concentrations of rilapladib, its major metabolite, SB-664601, and other potential metabolites with 14 days of repeat oral dosing with 250mg of rilapladib

2.3. Exploratory

1. To evaluate the effects of 14 days repeat oral dosing with 250mg of rilapladib on platelet maturation as measured by platelet count and mean platelet volume (MPV) on Days 1 and 14 (during treatment) and 35 (or 21 days post last dose)
2. To evaluate whether isolated platelets derived from rilapladib treated subjects behave aberrantly in normal plasma as well as to determine the effect of plasma derived from rilapladib treated subjects on untreated platelets on Day 34 (+/- 4 days), if feasible
3. To evaluate the effects of rilapladib on the initial rate of aggregation as determined by the slope of the individual aggregometer tracing after shape change
4. To evaluate the effects of rilapladib on immune responses of platelets, as data permit

3. ENDPOINT(S)

3.1. Primary

1. Collagen EC50 values (concentration which induces 50% of maximal aggregation) on Day 35 (or 21 days post last dose) as determine by optical aggregometry

3.2. Secondary

1. Collagen EC50 values (concentration which induces 50% of maximal aggregation) on Day 1 and Day 14 as determined by optical aggregometry
2. Plasma Lp-PLA$_2$ activity, expressed in terms of percent inhibition relative to baseline, on Days 1, 14 and 35 (or 21 days post last dose)
3. Clinical safety data from spontaneous adverse event reporting, 12-Lead ECG, vital signs, nursing/physician observation and safety laboratory tests
4. Concentrations of rilapladib, its major metabolite, SB-664601, and other potential metabolites, on Days 1, 14 and 35 (or 21 days post last dose)

3.3. Exploratory

1. Platelet maturation as measured by platelet count and MPV during treatment (Days 1 and 14) and off drug period (Day 35)
2. Collagen EC50 values (concentration which induces 50% of maximal aggregation) for normal platelets mixed into the post-treatment plasma, and for the post-treatment platelets mixed into normal plasma, on Day 34 (+/- 4 days) only, if feasible
3. Collagen concentration which induces 50% of the maximal slope change on Day 1, 14 and 35
4. Platelet surface immunoglobulin measured by RIA or flow cytometry, as data permit

4. STUDY DESIGN

This will be a double blind, repeat dose, randomized, placebo-controlled, parallel study. Each subject will be randomized to either rilapladib or placebo and will be dosed for 14 days (in-house from Day -2 through Day 1; outpatient witnessed dosing Days 2-13; in house Day 13-14; discharge on Day 14). Platelet aggregation measurements will occur on Day -1, Day 1, Day 14 and Day 35 (relative to the start of dosing on Day 1). On Day 34 (+/- 4 days) a subset of subjects may have blood collected to be used for ex-vivo evaluation of the plasma and platelets on platelet aggregation when combined with normal (untreated) platelets and normal (untreated) plasma. The entire study will consist of a minimum of ~4 or 5 overnight stays and ~12 outpatient visits.

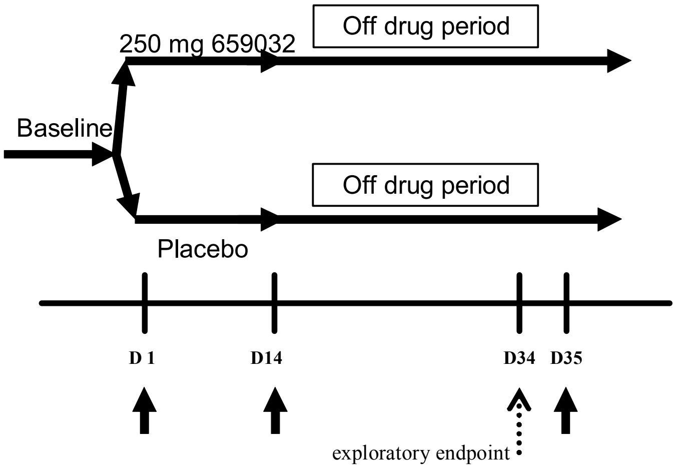

Regimen	Description
A	250 mg rilapladib QD for 14 days
B	Rilapladib matched placebo QD for 14 days

Each subject's participation in the study will be approximately 12 weeks from screening to follow-up.

5. STUDY POPULATION

5.1. Number of Subjects

Approximately 66 subjects are targeted for enrollment. Following completion of the first cohort (~28 subjects) there will be a sample size re-estimation to determine if the planned sample size is sufficient or if the sample size should be adjusted (see Section 12.2.3). Eligible subjects will be healthy adult males between 18 and 55 years of age who have a 10 year risk of cardiovascular events of <10% (Appendix 2).

5.2. Eligibility Criteria need to be reviewed

5.2.1. Inclusion Criteria

A subject will be eligible for inclusion in this study only if all of the following criteria apply:

1. Adult males between 18 and 55 years of age, inclusive
2. Ten year cardiovascular risk <10% (risk can be calculated using Framingham Point Scores in Appendix 2)
3. Healthy as determined by a responsible physician, based on a medical evaluation including history, physical examination, laboratory tests, cardiac monitoring. A subject with a clinical abnormality or laboratory parameters outside the reference range for the population being studied may be included only if the Investigator considers that the finding will not introduce additional risk factors and will not interfere with the study procedures
4. Body weight ≥ 50 kg (110 pounds) and body mass index (BMI) between 19 and 29.9 where:

$$\text{BMI} = \frac{\text{weight in kg}}{(\text{height in meters})^2}$$

5. Subjects with QTc < 450 msec as measured at screening
6. A signed and dated written informed consent prior to admission to the study
7. The subject is able to understand and comply with protocol requirements, instructions and protocol-stated restrictions.

5.2.2. Exclusion Criteria

A subject will not be eligible for inclusion in this study if any of the following criteria apply:

1. Any clinically relevant abnormality identified on the screening medical assessment, laboratory examination or ECG
2. Platelet count below or above the reference range
3. MPV outside of normal reference range
4. History of hypercoagulable state or history of thrombosis
5. History of platelet dysfunction
6. A known history of Gilbert's Syndrome
7. History of asthma, anaphylaxis or anaphalactoid reactions, severe allergic responses

8. The subject has a positive pre-study urine drug/ urine alcohol screen. A minimum list of drugs that will be screened for include Amphetamines, Barbiturates, Cocaine, Opiates, Cannabinoids and Benzodiazepines
9. A history of alcohol, substance or drug abuse within the last year or a positive alcohol breath test at screening or Day -1. Abuse of alcohol is defined as an average weekly intake of greater than 21 units or an average daily intake of greater than 3 units. 1 unit is equivalent to a middy (285mL) of full strength beer or a schooner (425mL) of light beer or 1 glass (100 mL) of wine or 1 glass (60mL) of sherry/portwine or 1 nip (30 mL) of spirits
10. History of use of tobacco or nicotine containing products within 6 months of screening or a positive urine cotinine at screening or exhaled carbon monoxide test at predose in each period
11. A positive pre-study HIV, Hepatitis B surface antigen or positive Hepatitis C antibody result within 3 months of screening
12. Use of aspirin, aspirin-containing products, non-steroidal anti-inflammatory agents or any antiplatelet medication within 14 days prior to the first platelet aggregation assessment (a list of these drugs will be reviewed with the subject at screening and provided to them to take home)
13. Use of prescription or non-prescription drugs within 7 days (or 14 days if the drug is a potential enzyme inducer) or 5 half-lives (whichever is longer) prior to the first dose of study medication, unless in the opinion of the Investigator and GSK medical monitor the medication will not interfere with the study procedures or compromise subject safety. An exception is paracetamol which is allowed at doses of ≤ 2g/day
14. Use of dietary/herbal supplements including (but not limited to) St. John's wort, kava, ephedra (ma huang), gingko biloba, DHEA, yohimbe, saw palmetto, ginseng and red yeast rice within 14 days prior to Day -1 of the study, unless in the opinion of the Investigator and GSK medical monitor the medication will not interfere with the study procedures or compromise subject safety
15. Exposure to more than four new chemical entities within 12 months prior to the first dosing day
16. The subject has participated in a clinical trial and has received a drug or a new chemical entity within 30 days or 5 half-lives, or twice the duration of the biological effect of any drug (whichever is longer) prior to the first dose of current study medication.
17. History of sensitivity to SB-659032, or components thereof or a history of drug or other allergy that, in the opinion of the physician responsible, contraindicates their participation
18. Consumption of grapefruit or grapefruit juice within 7 days prior to Day -1 of the study
19. A history of cholecystectomy or biliary tract disease including a history of liver disease with elevated liver function tests of known or unknown etiology

20. Where participation in study would result in donation of blood in excess of 500 mL within a 56 day period.
21. Subjects who do not aggregate fully (≥ 40%) in response to a collagen concentration of 10 µg/mL, as assessed at any time between screening and Day -1 (inclusive)
22. Subjects who have out of range EC50 values on Day -1
23. An unwillingness of subjects to either avoid direct sunlight, or to wear sunblock (SPF ≥ 30) if exposed to sunlight for more than 15 minutes, from the start of dosing until 14 days after the last dose

5.2.3. Other Eligibility Criteria Considerations

To assess any potential impact on subject eligibility with regard to safety, the investigator must refer to the Clinical Investigator's Brochure for detailed information regarding warnings, precautions, contraindications, adverse events, and other significant data pertaining to the investigational product(s) being used in this study [GlaxoSmithKline Document Number ZM2004/00059/01].

6. STUDY ASSESSMENTS AND PROCEDURES

A Time and Events Table is in Appendix 1.

Whenever multiple assessments are scheduled at the same time, the order of procedures should be as follows: ECG, vital signs, blood draws. Any samples collected for PK and/or PD analysis should be drawn at the exact nominal time.

Screening procedures can occur up to approximately 30 days prior to the first dose of study medication.

6.1. Demographic and Baseline Assessments

The investigator, or designate, will collect the following data and perform the following procedures for all subjects. Informed consent must be obtained from a subject prior to any study-specific procedures taking place.

6.1.1. Demography

The subject's date of birth, sex, ethnicity and race will be recorded in the CRF.

6.1.2. Medical History/medication

Relevant medical history (including tobacco and alcohol history) and current medication being taken by the subject will be collected.

6.2. Safety

6.2.1. Physical Exam

A complete physical exam will be obtained at the timepoints indicated in Appendix 1, and any clinically significant abnormalities will be captured as adverse events (AEs) (see Section 11). The height and weight of the subject will be recorded at the screening visit only.

6.2.2. Vital Signs

Blood pressure and heart rate will be measured at the timepoints indicated in Appendix 1. Measurement will be made after the subject sits in a semi-supine position quietly for at least 5 minutes.

6.2.3. 12-Lead Electrocardiogram (ECG) Monitoring

All 12-lead ECGs will be collected at the timepoints listed in Appendix 1. All 12-lead ECGs will be obtained after the subject has rested in the semi-supine position for at least 10 minutes. The 12-lead ECG tracings will be printed at a paper speed of 25 mm/sec in a '3 x 4' format.

The pre-dose 12-lead ECGs must be evaluated (for safety) by the investigator prior to the administration of the study medication. The 12-lead ECG obtained on Day 14 must be evaluated for safety by the investigator prior to discharge from the CRU. Any clinically significant ECG abnormalities observed at baseline and throughout the study will be documented in the CRF by the investigator.

6.2.4. Clinical Laboratory Measurement

6.2.4.1. Hematology, clinical chemistry and urinalysis

Blood and urine samples following an 8 hour fast will be taken at the timepoints listed in Appendix 1 for routine hematology and clinical chemistry.

Parameters to be tested are listed below.

Hematology

RBC Count	*RBC Indices*:	WBC Count
	MCV	*Automated WBC Differential:*
	MCH	Neutrophils
	MCHC	Lymphocytes
		Monocytes
		Eosinophils
		Basophils

Clinical Chemistry

BUN	Chloride	ALT (SGPT)	Direct Bilirubin
Creatinine	Total CO_2	AST (SGOT)	Uric Acid
Glucose, fasting	Calcium	GGT	Albumin
Sodium	Total Protein	Total Bilirubin	CPK
Potassium		Alkaline Phosphatase	

Other
Urine Drug Screen (screening, Day -1, Day 13, Day 34)
Urine cotinine (screening, Day -1, day 13, Day 34)
HIV, Hepatitis B surface antigen and Hepatitis C antibody (screening only)
PT, PTT (screening only)

Routine Urinalysis
Specific gravity
pH, glucose, protein, blood and ketones by dipstick
Microscopic examination (if dipstick is positive for blood or protein)

Normal reference ranges for all parameters are to be provided prior to starting the study. ***If, in the opinion of the Investigator, a laboratory test result becomes significantly abnormal, the GSK medical monitor should be contacted.*** Clinically significant abnormal laboratory findings should be recorded in the Adverse Events section of the CRF. Laboratory test values outside the normal range, but not clinically significant (NCS), should be marked "NCS" by the investigator on the report.

6.2.5. Pregnancy

Female subjects will not be enrolled into this study.

6.3. Deviations from the Time and Events Schedule

The precise timing of pharmacokinetic and/or pharmacodynamic sampling for blood may be altered during the course of the study based on newly available data (e.g. to obtain data closer to the time of peak plasma concentrations).

During the treatment phase, time deviations of up to 3 minutes on measurements scheduled up to 1 hour after dosing will not be regarded as protocol violations. Deviations of up to 5 minutes on measurements scheduled from 1 hour to 4 hour after dosing will not be regarded as protocol violations. Deviations of up to 30 minutes on measurements scheduled from 4 hour to 24 hour after dosing will not be regarded as protocol violations. Deviations of up to 60 minutes on measurements scheduled greater than 24 hours after dosing will not be regarded as protocol violations. However, the actual time should be recorded and the investigator should make every effort to perform procedures at the scheduled times.

6.4. Pharmacokinetics

6.4.1. Sample Collection and Preparation

Blood samples for pharmacokinetic analysis will be collected either via direct venipuncture or through a catheter kept patent with 0.9% sodium chloride solution (**heparin will not be used in this study**) and will be processed as described in the Study Reference Manual (SRM). The blood volume for each sample is documented in the SRM and the collection timepoints are indicated in Appendix 1 (PK samples will be collected at the same time samples are collected for platelet aggregation).

Urine samples will be collected for analysis of any metabolite(s) over a 24 hour period as indicated in Appendix 1. Urine collection procedures and processing instructions are included in the SRM.

6.4.2. Assay Methodology

Plasma samples will be assayed for rilapladib and its pharmacologically active N-desalkylated metabolite, SB-664601, using an approved assay methodology (on file at GlaxoSmithKline). Sample analysis will be the responsibility of World Wide Bioanalysis, Drug Metabolism and Pharmacokinetics, GlaxoSmithKline. Once the plasma has been analysed for SB-659032 and its metabolite SB-664601, any remaining plasma may be investigated for other circulating metabolites and the results reported under a separate DMPK protocol.

6.4.3. Planned Pharmacokinetic Evaluation

Due to incomplete sampling, maximum observed plasma concentration (Cmax), time to Cmax (Tmax) and area under the concentration-time curve (AUC) will not be calculated. Mean concentrations for rilapladib and SB-664601 will be calculated at each time point. The data will be summarized in a tabular form and descriptive statistics will be calculated at each timepoint.

6.5. Pharmacodynamics

6.5.1. Sample Collection and Preparation

Platelet Aggregation

Blood samples will be collected at the timepoints listed in Appendix 1. Blood samples for ex vivo platelet aggregation measurements via optical aggregometry will be collected and prepared as described in the SRM.

A subset of subjects may have blood samples collected on Day 34 (+/- 4 days) for ex-vivo evaluation of the subject plasma and platelets on platelet aggregation when combined as follows: 1) normal platelets + rilapladib treated plasma, 2) rilapladib treated platelets + normal plasma, 3) normal platelets + normal plasma, 4) rilapladib treated

platelets+ rilapladib treated plasma (the latter two serve as controls and won't be used in the final analysis). The plasma and platelets will be collected and combined as described in the SRM. The subset of subjects to be used will be randomly selected after taking into consideration: (1) subject availability for this extra commitment, and (2) feasibility of performing these additional assessments, as determined by the site. The selection process will be documented by the site and stored in the study file. Additional guidance on selection of subject to ensure balance between active and placebo will be provide in the SRM.

Lp-PLA$_2$ Activity

Blood samples for pharmacodynamic analysis will be collected at the timepoints listed in Appendix 1. These samples will be processed as described in the SRM.

Platelet Surface Immunoglobulin

Blood samples will be collected at the timepoints listed in Appendix 1 for measurement of platelet surface immunoglobulin by flow cytometry. Blood samples will be collected and prepared as described in the SRM.

Platelet Maturation Measurements

Platelet maturation will be assessed by measurements of platelet count and mean platelet volume (MPV) at the timepoints indicated in Appendix 1.

6.5.2. Assay Methodology

Platelet Aggregation

All ex vivo platelet aggregometry will be performed according to the approved methodology using platelet rich plasma (PRP). Collagen will be used as the agonists for the platelet-induced aggregation studies. The range of collagen concentrations to be used will contain values up to 10 μg/mL; the number of and actual concentrations to be used will be specified in the SRM. Single aggregation curves will be generated for each collagen concentration used at each timepoint.

Lp-PLA$_2$ Activity

Plasma samples will be assayed for Lp-PLA$_2$ activity using an approved method under the management of GlaxoSmithKline Pharmaceuticals.

Platelet Surface Immunoglobulin

Plasma samples will be measured by flow cytometry using an approved method under the management of GlaxoSmithKline Pharmaceuticals.

6.5.3. Planned Pharmacodynamic Evaluation

Platelet Aggregation

The percentage of maximal aggregation will be determined for each aggregometer tracing. In addition, the initial rate of aggregation will also be evaluated by measuring the slope of the individual aggregometer tracings. The slope on the aggregometer tracing reflects the initial rate of aggregation, which is an independent parameter of platelet responsiveness. All platelet aggregation tracings will be over-read and evaluated by an external blinded hematologist. This will be the final data set to be databased.

Details of the platelet aggregation analyes will be provided in the RAP.

Lp-PLA$_2$ Activity

The primary parameter, plasma Lp-PLA$_2$ activity, will be summarized as percent inhibition of plasma Lp-PLA$_2$ activity using the following formula:

$$100 \times [\ (\text{Activity}_{\text{at pre-dose on Day 1}} - \text{Activity}_{\text{at time x}}) / \text{Activity}_{\text{at pre-dose on Day 1}}\ ]$$

where Activity represents plasma Lp-PLA$_2$ activity.

Additional summary measures may be calculated as appropriate.

Platelet Surface Immunoglobulin

Details of this analysis will be provided in the RAP.

6.6. Pharmacogenetics

Information regarding pharmacogenetic research is included in Appendix 2. The IEC/IRB and, where required, the applicable regulatory agency must approve the PGx assessments before these can be conducted at the site. The approval(s) must be in writing and will clearly specify approval of the PGx assessments (i.e., approval of Appendix 2). In some cases, approval of the PGx assessments can occur after approval is obtained for the rest of the study. If so, then the written approval will clearly indicate approval of the PGx assessments is being deferred and in most cases, the study, except for PGx assessments, can be initiated. When PGx assessments will not be approved, then the approval for the rest of the study will clearly indicate this and therefore, PGx assessments will not be conducted.

7. LIFESTYLE AND/OR DIETARY RESTRICTIONS

7.1. Meals and Dietary Restrictions

- Subjects will not be allowed to drink grapefruit juice or eat grapefruit within 7 days prior to Day -1 until collection of the final blood sample in the study (e.g. Day 35)

- Water may be consumed ad libitum beginning 2 hour after dosing; soft drinks without caffeine or fruit juices (except grapefruit) may be consumed ad libitum beginning 4 hours after dosing
- Subjects must fast from all food and drink (except water) at least 8 hours prior to any clinical laboratory tests.
- Subjects should not consume the foods/food additives listed in the table below for 24 hours prior to Day -1 until collection of the final blood sample in the study (e.g. Day 35).

  Omega-3 fatty acids-eicosapentaenoic acid/docosahexaenoic acid (i.e., fish oil)
  Ethanol
  Chinese black tree fungus (used in chinese food)
  Onion extract
  Garlic (ajoene, a component of garlic)
  Cumin
  Tumeric
  Red wine
  Ginger
  Red Clover
  Horse chestnut
  Cat's claw
  Dong quai
  Evening primrose
  Feverfew
  Green tea

- All subjects will be provided with the same low fat meals prepared without the foods/food additives mentioned above during their inpatient stay.
- Subjects will be admitted to the facility on Days -2, 13 and 34 and may be served dinner. A subset of subjects may also be admitted to the facility one additional time, on the day before the Day 34 (+/- 4 days), and may also be served dinner on this day.
- On Days -1, 1, 14 and 35 subjects will not have breakfast. Subjects may be served lunch (after 6 hr blood collection for platelet aggregation) and dinner (at approximately 10 hrs after dosing). An evening snack will be permitted up to 2200 hours. These guidelines also apply to Day 34 (+/- 4 days) for the subset of subjects that may have a blood collection on this day.
- On Days 2 to 13 a low-fat breakfast will be provided 0.5 hour prior to dosing (the entire meal must be consumed within 20 minutes and subjects must be dosed within less than 10 minutes after completing the meal). This breakfast will be approximately 488 calories and consists of approximately 12 g fat, 28 g protein, and 77 g carbohydrates.

7.2. Caffeine and Alcohol

- Subjects will abstain from ingesting alcohol, caffeine- and xanthine-containing products for 24 hours prior to admission on Day -1 and until collection of the final blood sample for platelet aggregation testing on Day 14.
- Subjects will also abstain from ingesting alcohol, caffeine- and xanthine-containing products for 24 hours prior to admission to the CRU for additional platelet aggregation testing after the off-drug period and until discharge.

7.3. Activity

- Subjects will abstain from strenuous exercise for 72 hours prior to each blood collection for clinical laboratory tests.
- Subjects will be asked to avoid direct exposure to natural or artificial sunlight from administration of the first dose until 3 days after the last dose. Areas of skin exposed to direct sunlight for more than 15 minutes should be protected with sunblock (SPF ≥ 30).

8. INVESTIGATIONAL PRODUCT(S)

8.1. Description of Investigational Product

Rilapladib and rilapladib matched placebo will be supplied by GlaxoSmithKline as 12 mm, white, film coated, round, biconvex tablets. The non-bioenhanced, non-enteric coated, freebase formulation of rilapladib will be available in 250 mg tablet strengths.

8.2. Dosage and Administration

Subjects will be randomized to receive study medication prior to dosing on Day 1 in accordance with the randomization schedule. Subjects will be provided a low-fat breakfast starting 30 minutes prior to dosing, and meal must be consumed entirely within 20 minutes. Subjects will be administered 1 tablet by study personnel with approximately 240 mL of water.

Regimen	Description
A	250 mg non-enteric coated rilapladib QD for 14 days
B	Rilapladib matched placebo QD for 14 days

8.3. Dose Rationale

The 250 mg dose of non-enteric coated formulation of rilapladib was chosen based on the pharmacodynamic data (inhibition of plasma Lp-PLA2 activity) noted in Study 002 where a non-enteric coated formulation of rilapladib was studied. In the fasted state, doses of 240 and 400 mg resulted in >90% inhibition of plasma Lp-PLA2 activity at Cmax following a single dose. In the current study, rilapladib will be administered with

food to minimize the formation of 2,3-diFBCl. The fact that preliminary data suggest a clinically significant food effect (i.e. increased exposure) when non-enteric coated formulation of rilapladib is dosed with a high-fat meal, the dose of the non-enteric coated rilapladib will be reduced to 250 mg in the current study so that the exposure at 400 mg is not exceeded. Extrapolating the observed food effect at a dose of 120 mg in Study 002 and assuming that the magnitude of food effect is similar with a high-fat breakfast (administered in Study 002) and a low-fat breakfast (to be administered in current study), with the 250 mg dose planned in the current study, the observed exposure is anticipated to be within the range noted in Study 002 where repeat, oral doses up to 400 mg were found to be safe and well tolerated. The ultimate goal of Lp-PLA2 inhibition is to inhibit the activity of the enzyme within atherosclerotic lesions (the site of Lp-PLA2 hydrolytic activity).

8.4. Blinding

This is a double blind study.

An investigator or other physician managing the subject may decide to unblind that subject's treatment code, only in the case of a **medical emergency or in the event of a serious medical condition**, when knowledge of the investigational product is essential for the clinical management or welfare of the subject. The investigator will make every effort to contact the GSK Medical Monitor, on-call physician (if applicable), or appropriate GSK study personnel before unblinding to discuss options. If the blind is broken for any reason and the investigator is unable to contact GSK prior to unblinding, the investigator must notify GSK **as soon as possible following** the unblinding incident **without revealing the subject's study treatment assignment,** unless the information is important to the safety of subjects remaining in the study. In addition, the investigator will record the date and reason for revealing the blinded treatment assignment for that subject in the appropriate data collection tool (as defined in Section 13.7).

If a serious adverse event (SAE; as defined in Section 11.2, "Definition of an SAE") is reported to GSK, Global Clinical Safety and Pharmacovigilance (GCSP) staff may unblind the treatment assignment for the individual subject. If an expedited regulatory report to one or more regulatory agencies is required, the report will identify the subject's treatment assignment. When applicable, a copy of the regulatory report may be sent to investigators in accordance with relevant regulations, GSK policy, or both.

An interim sample size re-estimation using complete data for the first cohort of 28 subjects will be conducted by an independent, unblinded statistician. Procedures will be employed to maintain the integrity of the blind, and ensure that treatment assignments will remain blinded to the study subjects, study investigators, and all other personnel directly involved with the conduct of the study.

8.5. Treatment Assignment

Subjects will be randomly assigned in a double-blind fashion to regimen A or B in accordance with the randomization schedule prepared in advance of the study by Clinical

Pharmacology Statistics and Programming, GlaxoSmithKline, using internal validated software (i.e., RandAll).

8.6. Packaging and Labeling

The contents of the label will be in accordance with all applicable regulatory requirements.

8.7. Preparation

No specific preparation of study medication is required prior to administration.

8.8. Handling and Storage

Rilapladib tablets will be stored up to 30°C (86°F) and protected from light and moisture. Investigational product must be dispensed or administered according to procedures described herein. Only subjects enrolled in the study may receive investigational product, in accordance with all applicable regulatory requirements. Only authorized site staff may supply or administer investigational product. All investigational products must be stored in a secure area with access limited to the investigator and authorized site staff and under physical conditions that are consistent with investigational product-specific requirements.

8.9. Product Accountability

The investigator, institution, or the head of the medical institution (where applicable) is responsible for investigational product accountability, reconciliation, and record maintenance. In accordance with all applicable regulatory requirements, the investigator or the head of the medical institution (where applicable), or designated site staff (e.g., storage manager, where applicable) must maintain investigational product accountability records throughout the course of the study. The responsible person(s) will document the amount of investigational product received from and returned to GSK (when applicable), the amount supplied and/or administered to and returned by subjects, if applicable.

8.10. Assessment of Compliance

Study medication will be administered under the supervision of study personnel. The oral cavity and hands of each subject will be examined following dosing to assure that study medication was taken.

A record of the amount taken by the subject will be maintained.

8.11. Treatment of Investigational Product Overdose

Any signs or symptoms of overdose will be treated symptomatically. No specific antidote is known.

8.12. Occupational Safety

Investigational product is not expected to pose significant occupational safety risk to site staff under normal conditions of use and administration. A Material Safety Data Sheet (MSDS)/equivalent document describing occupational hazards and recommended handling precautions either will be provided to the investigator, where this is required by local laws, or is available upon request from GSK.

9. CONCOMITANT MEDICATIONS AND NON-DRUG THERAPIES

9.1. Permitted Medications

All concomitant medications taken during the study will be recorded in the CRF. The minimum requirement is that drug name and the dates of administration are to be recorded.

Acetaminophen at doses of ≤ 2 grams/day is the only permitted medication.

All other medications are prohibited (as described in Sections 9.2), unless the medical monitor and PI agree that a subject needs a specific medication.

9.2. Prohibited Medications

Subjects will abstain from using prescription or over-the-counter medications (other than acetaminophen as described above) within 7 days or 5 half-lives (whichever is longer) prior to Day 1 and until the follow-up visit. Use of any anticoagulants, aspirin, aspirin-containing products, or non-steroidal anti-inflammatory agents are not allowed within 14 days prior to Day 1 and until the follow-up visit.

9.3. Non-Drug Therapies

The use of vitamins is prohibited within 7 days prior to Day 1 and until the follow-up visit.

The use of herbal and dietary supplements is prohibited within 14 days prior to Day 1 and until the follow-up visit.

10. SUBJECT COMPLETION AND WITHDRAWAL

10.1. Subject Completion

Subjects who complete all protocol-specified procedures will be considered as having completed the study. Subjects for whom sufficient safety, PK and/or PD data have been collected will be considered as evaluable subjects.

10.2. Subject Withdrawal

10.2.1. Subject Withdrawal from Study

Subjects may withdrawal from the study at any time at their own request, or may be withdrawn at any time at the discretion of the investigator for safety, behavioral, or administrative reasons.

10.2.2. Subject Withdrawal from Investigational Product

Subjects withdrawn from the investigational product (i.e., during the treatment phase of the study) will undergo vital signs measurement, 12-Lead ECG, blood and urine collection for clinical laboratory tests and an AE assessment prior to discharge. In addition the subject should come back for a follow up visit approximately 14-21 days after their last dose of study medication and undergo vitals signs measurement, 12-lead ECG, blood and urine collection for clinical laboratory test and an AE assessment.

Unevaluable subjects may be replaced with another subject assigned the same sequence of treatments, with approval from GlaxoSmithKline.

10.3. Screen and Baseline Failures

Subjects who do not meet the inclusion/exclusion criteria, including those subjects with clinical laboratory values such as a positive urine drug screen, will be considered screen failures and will not have data captured in the CRF.

11. ADVERSE EVENTS (AE) AND SERIOUS ADVERSE EVENTS (SAE)

The investigator is responsible for the detection and documentation of events meeting the criteria and definition of an AE or SAE, as provided in this protocol. During the study when there is a safety evaluation, the investigator or site staff will be responsible for detecting, documenting and reporting AEs and SAEs, as detailed in both this section of the protocol and in the AE/SAE section of the SRM.

11.1. Definition of an AE

Any untoward medical occurrence in a patient or clinical investigation subject, temporally associated with the use of a medicinal product, whether or not considered related to the medicinal product.

Note: An AE can therefore be any unfavorable and unintended sign (including an abnormal laboratory finding), symptom, or disease (new or exacerbated) temporally associated with the use of a medicinal product.

11.2. Definition of a SAE

A serious adverse event is any untoward medical occurrence that, at any dose:

a. Results in death

b. Is life-threatening

   NOTE: The term 'life-threatening' in the definition of 'serious' refers to an event in which the subject was at risk of death at the time of the event. It does not refer to an event, which hypothetically might have caused death, if it were more severe.

c. Requires hospitalization or prolongation of existing hospitalization

   NOTE: In general, hospitalization signifies that the subject has been detained (usually involving at least an overnight stay) at the hospital or emergency ward for observation and/or treatment that would not have been appropriate in the physician's office or out-patient setting. Complications that occur during hospitalization are AEs. If a complication prolongs hospitalization or fulfills any other serious criteria, the event is serious. When in doubt as to whether "hospitalization" occurred or was necessary, the AE should be considered serious.

   Hospitalization for elective treatment of a pre-existing condition that did not worsen from baseline is not considered an AE.

d. Results in disability/incapacity, or

   NOTE: The term disability means a substantial disruption of a person's ability to conduct normal life functions. This definition is not intended to include experiences of relatively minor medical significance such as uncomplicated headache, nausea, vomiting, diarrhea, influenza, and accidental trauma (e.g. sprained ankle) which may interfere or prevent everyday life functions but do not constitute a substantial disruption.

e. Is a congenital anomaly/birth defect

f. Medical or scientific judgement should be exercised in deciding whether reporting is appropriate in other situations, such as important medical events that may not be immediately life-threatening or result in death or hospitalization but may jeopardize the subject or may require medical or surgical intervention to prevent one of the other outcomes listed in the above definition. These should also be considered serious. Examples of such events are invasive or malignant cancers, intensive treatment in an emergency room or at home for allergic bronchospasm, blood dyscrasias or convulsions that do not result in hospitalization, or development of drug dependency or drug abuse.

11.2.1. Clinical Laboratory Abnormalities and Other Abnormal Assessments as AEs and SAEs

Abnormal laboratory findings (e.g., clinical chemistry, hematology, and urinalysis) or other abnormal assessments (e.g. ECGs and vital signs) that are judged by the investigator as **clinically significant** will be recorded as AEs or SAEs if they meet the definition of an AE or SAE. Clinically significant abnormal laboratory findings or other

abnormal assessments that are detected during the study or are present at baseline and significantly worsen following the start of the study will be reported as AEs or SAEs. However, clinically significant abnormal laboratory findings or other abnormal assessments that are associated with the disease being studied, unless judged by the investigator as more severe than expected for the subject's condition, or that are present or detected at the start of the study and do not worsen, will **not** be reported as AEs or SAEs.

The investigator will exercise his or her medical and scientific judgment in deciding whether an abnormal laboratory finding or other abnormal assessment is clinically significant.

11.2.1.1. QTc Stopping Criteria

Subjects will be withdrawn from the study if QTc measurement from any 12-lead ECG exceeds 500 msec.

11.2.1.2. Liver Chemistry Stopping Criteria

Liver chemistry threshold stopping criteria have been designed to assure subject safety. When subjects meet the following liver chemistry threshold criteria, investigational product must be permanently withdrawn, additional testing performed, and the subject monitored until liver chemistries resolve, stabilize, or return to baseline values. The subject must then be permanently withdrawn from the study:

- ALT ≥ 3xULN **and** bilirubin ≥ 1.5xULN (>35% direct).
- ALT ≥ 3xULN.

Subjects with ALT ≥3xULN **and** bilirubin ≥1.5xULN (>35% direct bilirubin; bilirubin fractionation required) must be immediately and permanently withdrawn from investigational product. Every attempt must be made to have the subject return to clinic (within 24 hours) for repeat liver chemistries and additional testing, and monitored closely (with specialist or hepatology consultation recommended). This event must be reported to GSK within 24 hours of learning of its occurrence. Subjects must be monitored twice weekly until liver chemistries (ALT, AST, alkaline phosphatase, bilirubin) resolve, stabilize or return to within baseline values. Upon completion of the safety follow-up, the subject must then be withdrawn from the study.

Subjects with ALT ≥3xULN must be permanently withdrawn from investigational product and monitored weekly until liver chemistries (ALT, AST, alkaline phosphatase, bilirubin) resolve, stabilize or return to within baseline values. This event must be reported to GSK within 24 hours of learning of its occurrence.

In all the above situations, every attempt must be made to obtain the following:

- Viral hepatitis serology including:
    - Hepatitis A IgM antibody.

  - Hepatitis B surface antigen and Hepatitis B Core Antibody (IgM).
  - Hepatitis C RNA.
  - Cytomegalovirus IgM antibody.
  - Epstein-Barr viral capsid antigen IgM antibody (or if unavailable, obtain heterophile antibody or monospot testing).
  - Hepatitis E IgM antibody (if subject resides outside the USA or Canada, or has traveled outside USA or Canada in past 3 months).
- Blood sample for pharmacokinetic (PK) analysis, obtained within 24 hours or 3 half-lives <insert value of whichever is longer> of last dose. Record the date/time of the PK blood sample draw and the date/time of the last dose of investigational product prior to blood sample draw on the CRF.
- Serum creatine phosphokinase (CPK) and lactate dehydrogenase (LDH).
- Fractionate bilirubin, if bilirubin≥1.5xULN.
- Record the appearance or worsening of clinical symptoms of hepatitis, or hypersensitivity, fatigue, decreased appetite, nausea, vomiting, abdominal pain, jaundice, fever, or rash as relevant on the AE report form.
- Record use of concomitant medications, acetaminophen, herbal remedies, other over the counter medications, putative hepatotoxins, or alcohol on the concomitant medications report form.

The following are required for subjects with ALT ≥3xULN **and** bilirubin ≥1.5xULN but are optional for other abnormal liver chemistries:

- Anti-nuclear antibody, anti-smooth muscle antibody, and Type 1 anti-liver kidney microsomal antibodies.
- Liver imaging (ultrasound, magnetic resonance, or computerized tomography) to evaluate liver disease.

11.3. Time Period, and Frequency of Detecting AEs and SAEs

Any pre-existing conditions or signs and/or symptoms present in a subject prior to the start of the study (i.e., before informed consent) should be recorded as Medical/Surgical History. In addition, any medical occurrence which is reported after informed consent is obtained but prior to the start of dosing will be documented as Medical/Surgical History. Any medical occurrences which present after the start of dosing and up to and including the final Follow-up Visit must be reported as AEs or SAEs. All AEs and SAEs must be recorded irrespective of whether they are considered drug related.

From the time a subject consents to participate in the study until he or she has completed the study (including any follow-up period), all SAEs assessed as related to study participation (e.g., protocol-mandated procedures, invasive tests, or change in existing therapy) or related to a GSK concomitant medication, will be reported promptly to GSK.

11.4. Prompt Reporting of SAEs to GSK

SAEs will be reported promptly to GSK as described in the following table once the investigator determines that the event meets the protocol definition of an SAE.

11.4.1. Timeframes for Submitting SAE Reports to GSK

	Initial SAE Reports		Follow-up Information on a Previously Reported SAE	
Type of SAE	Time Frame	Documents	Time Frame	Documents
All SAEs	24 hrs	"SAE" data collection tool	24 hrs	Updated "SAE" data collection tool

11.5. AE and SAE Documentation and Follow-up Procedures

The investigator will review and adhere to the following procedures, which are outlined in detail in the AE/SAE section of the SRM:

- Method of Detecting AEs and SAEs
- Recording of AEs and SAEs
- Evaluating of AEs and SAEs
- Completion and Transmission of SAE Reports to GSK
- Follow-up of AEs and SAEs
- Post-study AEs and SAEs
- Regulatory Reporting Requirements for SAEs

12. DATA ANALYSIS AND STATISTICAL CONSIDERATIONS

Statistical analysis will be performed by, or under the direct auspices of, Clinical Pharmacology Statistics and Data Sciences, GlaxoSmithKline.

12.1. Hypotheses

The statistical framework will be to demonstrate a lack of increased effect of the 14 days of repeat oral doses of 250 mg of rilapladib on EC50 of collagen as compared to placebo at Day 35 (21 days post last dose). This will be evaluated using a non-inferiority testing framework.

The null and alternative hypotheses to be tested are as follows:

$$H_0 : \frac{\mu_T}{\mu_P} \leq 1 - E$$

$$H_1 : \frac{\mu_T}{\mu_P} > 1 - E$$

where μ_T and μ_P are the mean EC50 values for the active group and placebo group, respectively, at Day 35, and E is the magnitude of a relative margin of equivalence of 15%.

Lack of increased effect will be demonstrated (that is the null hypothesis will be rejected), if the lower bound of a two-sided 95% confidence interval for the estimated ratio of active dose versus placebo (allowing for possible baseline differences in EC50 between subjects) (A: B, on Day 35) is greater than 85%.

12.2. Study Design Considerations

12.2.1. Sample Size Assumptions

The following model is used for both the sample size calculation and the subsequent data analysis. Emin, Emax and EC50 represent the parameters for respectively, the minimum and maximum aggregation levels (in % scale) and the log of the collagen concentration corresponding to the middle (mean) of Emin and Emax as determined by optical aggregometry.

The applied Emax model is:

$$y = E_{\max} - \frac{E_{\max} - E_{\min}}{1 + c}$$, where

y represents the platelet aggregation (in % scale) as determined by optical aggregometry, and c represents:

c = exp((logCollagenConcentration – EC50 + SubjectEffect) * Gamma)

The gamma parameter controls the slope between platelet aggregation (%) and collagen concentration.

The EC50 for log of the collagen concentration is determined by maximum likelihood estimation using fixed values for the Emin, Emax and Gamma parameters. Iteratively re-weighted least squares is used with the variance-mean relationship determined by

Variance = **1** + S2 * (abs(PredictedValue) * abs(**100**-PredicetedValue))

The value 1 makes sure that the variance is non-zero when the predicted value gets close to 0 or 100. The scale parameter S2 is estimated as a parameter in the maximum likelihood procedure.

In this sample size calculation, Emax and Emin were set at 5% and 95% while Gamma has been set at 6. These values were chosen based on the analysis of data from a pilot study with a few individual subjects using a wide range of collagen concentrations (eight or more collagen levels).

These values were used to calculate EC50 values of log-collagen-concentration for each sample in period 1 from the previous study [Data on file, SB-659032/LP2104623, GSK], in which only four collagen concentrations (0.7, 1, 1.2 and 10 µgm/mL) were evaluated. Due to the limited concentrations used in the previous study, the estimates are expected to be more variable than those in the current study. However they can provide evidence for how well the baseline should predict the day 35 response.

In this parallel group study the ability of the baseline to control variation is an important aspect of the sample size estimation. Combining the data at day 35 across the three times into a single analysis, the between subject variation in EC50 at day 35 is estimated as 0.142 and the residual is 0.107. The magnitude of the residual is considered to be mostly due to assay sensitivity (ie., high assay variability) with only four collagen levels. These values are adjusted for the baseline covariate. Without adjusting for baseline, the variances would be 0.214 and 0.107.

To estimate the between-subject variation excluding the assay variation, a random effects Emax model was fitted to data from the previous study LP2104623 using Period 1 only again. A maximum likelihood approach was used to estimate model parameters; Emax, Emin, EC50 for each treatment and Time effects as well as the estimated between-subject variance on EC50, and the residual variance on the percentage aggregation scale. The gamma parameter was fixed at 6 as above. The residual estimation used the same iteratively re-weighted least squares approach. The estimated between-subject variation in EC50 at day 35 was 0.142. This is value is not adjusted for baseline covariate.

In order to estimate the assay variability when using the collagen levels prescribed for the current study, a simulation was carried out with 10 collagen levels (0.2, 0.5, 0.7, 0.9, 1.1, 1.3, 1.5, 1.7, 2 and 10 µgm/mL). The data were simulated using an Emax model with parameter values; Emax and Emin at 5% and 95% and gamma at 6. The between-subject and residual variation in aggregation percentages were chosen from the previous modeling (0.142 for the between-subject and S2=0.071 in the variance formula). From the simulated data, an EC50 was estimated using the Emax model with fixed Emin, Emax and Gamma based on the 10 simulated aggregation values. The variation across simulations of the resulting EC50 values was estimated from 10000 simulations. When the EC50 was in the middle of the collagen range, between 0.5 and 2, the additional variation above the 0.142 between-subject variation introduced into the simulated data was around 0.005, rising rapidly if EC50 is outside this range.

Applying the same approach with only four collagen levels (0.7, 1, 1.2 and 10 µgm/mL) the variance component from the assay level was 0.169 based around 1.2 µgm/mL in the

middle of the range where much of the data were located, but it rises quickly as EC50 moves in either direction within the range.

This suggests that the variance in the EC50 at day 35 in this study will be (0.142+0.005)=0.147 rather than (0.142+0.169)=0.311 when using only the 4 collagen levels. Note that 0.311 is close to the 0.321 (=0.214+0.107) observed for the absolute estimated EC50 when uncorrected for baseline, as described above.

This suggests that adjusting for the baseline reduces the variance by 0.072 (0.214-0.142), so the effective variance is estimated as (0.142 – 0.072+0.005) = 0.075. The standard deviation is 0.27. Note that this has not taken account of the replication, 0 and 6 hours, on Day 35. Also all the assumptions in the calculation of the variation have been conservative, especially for the between-subject variation which is critical. Therefore, the estimated standard deviation of 0.27 is most probably an over-estimate of the standard deviation which might be observed in the current study. Thus, in order to obtain a more realistic and feasible sample size, the initial sample size will be estimated using an arbitrarily lower assumed standard deviation of 0.2. Note that the planned sample-size re-estimation will allow for adjustments to the sample size based on the actual/observed variability during the course of the study.

The non-inferiority testing framework is based on a clinically specified non-inferiority limit of 85% (100-15%). With a sample size of 66 (~33 subjects per treatment group) there will be at least 90% power to show non-inferiority when there is no difference between the treatment arms, assuming a standard deviation of 0.2.

12.2.2. Sample Size Sensitivity

The following sample size sensitivity analysis was conducted, under the assumption that the standard deviation for the observed study is 0.27. Based on the non-inferiority limit of 85% (100-15%), and a standard deviation of 0.27 as estimated above, a sample size of approximately 122 subjects (~61 subjects per treatment group) would provide at least 90% power to show non-inferiority when there is no difference between the treatment arms.

12.2.3. Sample Size Re-estimation

The study is planned to be conducted in cohorts (~28 subjects per cohort initially) with a maximal capacity to evaluate approximately 84 subjects in total.

An interim sample size re-estimation using complete data for the first cohort (~28 subjects) will be conducted by an independent unblinded statistician. Procedures will be employed to maintain the integrity of the blind, and ensure that treatment assignments will remain blinded to the study subjects, study investigators, and all other personnel directly involved with the conduct of the study.

After approximately 28 subjects (about 14 subjects per treatment group) have completed the study assessment on Day 35, their aggregation data will be analyzed to obtain better estimates for the population Emax model parameters. The non-inferiority limit will

remain at 85% and a revised sample size estimate (N1) for each treatment group will be calculated to obtain a power of 90%.

The standard deviation to be used in the sample size re-estimation will be calculated as follows. The analysis will involve two-stages. Firstly the Emax model will be fitted to the pre-treatment aggregation data (samples at 0, 6 and 24 hour on Day -1) with a random effect for subject on the EC50 scale, and a separate EC50 for the three time points. The estimated values for Emin, Emax and Gamma will be used in the second stage. The resulting EC50 values for log-collagen-concentration will be averaged separately for baseline and Day 35, to give one baseline value and one Day 35 measurement for each subject. A simple one-way analysis of variance model will be fitted to the Day 35 measurement using the baseline as a covariate. The standard deviation for the sample size re-estimation will be obtained from the estimated residual from this analysis.

(1) If N1 is less than 33 per group, the final target sample size may not be reduced. The study will continue as planned to enroll enough subjects to ensure that approximately 66 subjects (33 per group) complete the study.

(2) If N1 is larger than 33 per group but less than 42 per group, the study will continue to enroll the remaining cohorts of subjects in an effort to enroll up to 84 subjects (42 per group) to ensure at least (N1+N1) subjects complete the study.

(3) If N1 is larger than 42 but less than 84, the study will continue to enroll the remaining cohorts of subjects. Then a total of 84 subjects (42 per group) will be recruited and a reduced power for the study will be accepted.

(4) If N1>84 then it will be assumed that variability in the aggregation data was been poorly controlled and recruitment will be halted and the study will be terminated.

Based on the outline above, the independent unblinded statistician will provide the team with one of the following three recommendations at the interim assessment

(1) Enroll a sufficient number of subjects to ensure that a total of approximately 66 subjects complete the study

(2) Enroll the maximum capacity of 84 subjects into the study

(3) Terminate the study due to futility based on the variability.

If the target sample size of 66 subjects will not be reduced, no modification to the analysis (ie., alpha adjustment) is needed in light of the sample size re-estimation.

As statistically appropriate, an a priori decision rule to allow for the reduction of the target sample size to less than 66 subjects may be considered. Full details of this procedure, if adopted, and including alpha adjustment details, will be defined in the RAP, prior to the receipt of any data.

12.2.3.1. Other Comparisons of Interest

The other comparisons of interest will include:

(1) The repeat oral doses of rilapladib versus placebo (A:B) in EC50 of collagen on Day 1, and Day 14

(2) As appropriate to the data, the repeat oral doses of rilapladib will be compared to placebo in other platelet aggregation related endpoints (e.g. platelet count and MPV, Collagen EC50 values for normal platelets mixed with the post-treatment plasma, and Collagen EC50 values for the post-treatment platelets mixed with the normal plasma, and collagen EC50 values (concentration which induces the 50% of the maximal slope change) on each Day of interest (as specified in Section 3.3 and Appendix 1).

(3) The repeat oral doses of rilapladib versus placebo (A-B) in percent LpPla2 inhibition from baseline on Day 1, Day 14, Day 34 and Day 35 at each time point of interest (as specified in Appendix 1).

12.2.3.2. Pharmacodynamic Analyses

All pharmacodynamic endpoints will be listed and summarized descriptively by treatment group and time.

Platelet Aggregation Data Analyses

<u>Primary Endpoint</u>

The applied Emax model is:

$y = E_{\max} - \frac{E_{\max} - E_{\min}}{1+c}$ where y represents the platelet aggregation (in % scale) as determined by optical aggregometry, and c represents,

c = exp((logCollagenConcentration – EC50 + SubjectEffect) * Gamma)

The gamma parameter controls the slope between platelet aggregation (%) and collagen concentration.

The first step of the analysis will be to obtain estimated values for Emin, Emax and Gamma parameters for the study population.

Following $\log_e$ transformation of the concentration of collagen, the platelet aggregation (in % scale) as determined by optical aggregometry at baseline (0, 6 and 24 hours on Day -1), will be used to fit a non-linear mixed model using the maximum likelihood approach. The EC50 for log-collagen-concentration is determined by a linear predictor with a fixed effect term for time (i.e. 0, 6 and 24 hour on Day -1), while subject will be included as a random effect. Iteratively re-weighted least squares is used with the variance-mean relationship determined by Variance = 1 + S2 * (abs(PredictedValue) * abs(100-PredictedValue)), where S2 is an additional parameter in the model.

The Emin, Emax and Gamma estimates obtained from the above mentioned approach, will then be applied to the data (% platelet aggregation as determined by aggregometer) to estimate the EC50 value for log-collagen-concentration for each subject at each post-dose timepoint (i.e. 6 hour on Day 1; 0 and 6 hour on Day 14 and 35). That is separately for each blood sample.

Separate ANCOVA analysis will be performed to estimate the treatment effect at each day of interest (i.e. Day 1, Day 14 and Day 35). EC50 values for each subject at duplicate timepoints (e.g. 0 and 6 hour on Day 14 and Day 35) will be averaged before fitting in a mixed effect ANCOVA model. Treatment will be included as a fixed effect; and subject will be included as a random effect. The average of EC50 values from three duplicate baseline timepoints (0, 6 and 24 hour on Day -1) will be included as a covariate. Point estimates and associated 95% confidence intervals for the difference of A-B will be constructed using the residual variance. These point estimate and confidence intervals will then be exponentially back-transformed to obtain point estimates and associated 95% confidence interval for the ratio between A:B in EC50.

An alternative Bayesian analysis of the Emax random effect model may also be conducted in an exploratory fashion, but the primary objective will be evaluated based on the primary analysis methods outlined above. Details will be specified in the Reporting and Analysis Plan (RAP) prior to the database lock.

Exploratory Endpoints

As appropriate to the data, a similar analysis will be performed to provide point estimates and 95% CI for the comparisons of interest in the exploratory EC50 endpoints (i.e. collagen EC50 values to induce 50% maximal platelet aggregation collected following normal platelet mixed with post-treatment plasma, and following the normal plasma mixed with post-treatment platelet on Day 34; and the EC50 values to induce the 50% of the maximal slope change as determined by aggregometer tracing on Day 1, 14 and 35).

Additional formal statistical analyses may be conducted for other platelet related endpoints (e.g. platelet count and MPV, etc) to assess the treatment effect. Details will be specified in the Reporting and Analysis Plan (RAP) prior to the database lock.

Plasma LpPla2 Activity Data Analysis

Plasma LpPla2 activity data expressed as percent inhibition from baseline will be listed and summarized with descriptive statistics by regimen.

A formal statistical analysis will be conducted on the percent LpPla2 inhibition from baseline using a mixed effect model by fitting fixed effect terms (e.g. Treatment, Day, or as appropriate to the data) and including baseline value as covariate. Subjects will be treated as a random effect. Point estimates and 95% confidence intervals for the difference A-B at each time point evaluated will be estimated using appropriate error term.

13. STUDY CONDUCT CONSIDERATIONS

13.1. Regulatory and Ethical Considerations, Including the Informed Consent Process

GSK will obtain favorable opinion/approval to conduct the study from the appropriate regulatory agency in accordance with any applicable country-specific regulatory requirements prior to a site initiating the study in that country.

The study will be conducted in accordance with all applicable regulatory requirements.

The study will also be conducted in accordance with "good clinical practice" (GCP), all applicable subject privacy requirements, and, the guiding principles of the Declaration of Helsinki. This includes, but is not limited to, the following:

- IRB/IEC review and favorable opinion/approval to conduct the study and of any subsequent relevant amended documents
- Subject informed consent
- Investigator reporting requirements

GSK will provide full details of the above either verbally, in writing or both.

Written informed consent will be obtained for each subject before he or she can participate in the study.

13.2. Quality Control (Study Monitoring)

In accordance with applicable regulations, GCP, and GSK procedures, GSK monitors will contact the site prior to the start of the study to review with the site staff the protocol, study requirements, and their responsibilities to satisfy regulatory, ethical, and GSK requirements. When reviewing data collection procedures, the discussion will also include identification, agreement and documentation of data items for which the CRF will serve as the source document.

GSK will monitor the study consistent with the demands of the study and site activity to verify that the:

- Data are authentic, accurate, and complete.
- Safety and rights of subjects are being protected.
- Study is conducted in accordance with the currently approved protocol and any other study agreements, GCP, and all applicable regulatory requirements.

The investigator and the head of the medical institution (where applicable) agrees to allow the monitor direct access to all relevant documents

13.3. Quality Assurance

To ensure compliance with GCP and all applicable regulatory requirements, GSK may conduct a quality assurance audit. Regulatory agencies may also conduct a regulatory inspection of this study. Such audits/inspections can occur at any time during or after completion of the study. If an audit or inspection occurs, the investigator and institution agree to allow the auditor/inspector direct access to all relevant documents and to allocate his/her time and the time of his/her staff to the auditor/inspector to discuss findings and any relevant issues.

13.4. Study and Site Closure

Upon completion or premature discontinuation of the study, the monitor will conduct site closure activities with the investigator or site staff, as appropriate, in accordance with applicable regulations, GCP, and GSK procedures.

In addition, GSK reserves the right to temporarily suspend or prematurely discontinue this study at any time for reasons including, but not limited to, safety or ethical issues or severe non-compliance. For multicenter studies, this can occur at one or more or at all sites. If GSK determines such action is needed, GSK will discuss this with the investigator or the head of the medical institution (where applicable), including the reasons for taking such action, at that time. When feasible, GSK will provide advance notification to the investigator or the head of the medical institution, where applicable, of the impending action prior to it taking effect.

GSK will promptly inform all other investigators or the head of the medical institution (where applicable), and/or institutions conducting the study if the study is suspended or prematurely discontinued for safety reasons. GSK will also promptly inform the regulatory authorities of the suspension or premature discontinuation of the study and the reason(s) for the action. If required by applicable regulations, the investigator or the head of the medical institution (where applicable) must inform the IEC/IRB promptly and provide the reason for the suspension or premature discontinuation.

13.5. Records Retention

Following closure of the study, the investigator or the head of the medical institution (where applicable) must maintain all site study records, except for those required by local regulations to be maintained by someone else, in a safe and secure location. The records must be maintained to allow easy and timely retrieval, when needed (e.g., audit or inspection), and, whenever feasible, to allow any subsequent review of data in conjunction with assessment of the facility, supporting systems, and staff. Where permitted by local laws/regulations or institutional policy, some or all of these records can be maintained in a format other than hard copy (e.g., microfiche, scanned, electronic); however, caution needs to be exercised before such action is taken. The investigator must assure that all reproductions are legible and are a true and accurate copy of the original and meet accessibility and retrieval standards, including re-generating a hard copy, if required. Furthermore, the investigator must ensure there is an acceptable

back-up of these reproductions and that an acceptable quality control process exists for making these reproductions.

GSK will inform the investigator of the time period for retaining these records to comply with all applicable regulatory requirements. The minimum retention time will meet the strictest standard applicable to that site for the study, as dictated by any institutional requirements or local laws or regulations, or GSK standards/procedures; otherwise, the retention period will default to 15 years.

The investigator must notify GSK of any changes in the archival arrangements, including, but not limited to, the following: archival at an off-site facility, transfer of ownership of the records in the event the investigator leaves the site.

13.6. Provision of Study Results and Information to Investigators

When required by applicable regulations, the investigator signatory for the clinical study report will be determined at the time the report is written. When the clinical study report is completed, GSK will provide the investigator with a full summary of the study results. The investigator is encouraged to share the summary results with the subjects, as appropriate. In addition, the investigator will be given reasonable access to review the relevant statistical tables, figures, and reports and will be able to review the results for the entire study at a GSK site or other mutually agreeable location.

GSK will provide the investigator with the randomization codes for their site after the statistical analysis for the entire study has been completed.

13.7. Data Management

The data collection tool for this study will be GSK-defined case report forms (CRFs). In all cases, subject initials will not be collected nor transmitted to GSK. Subject data necessary for analysis and reporting will be entered/transmitted into a validated database or data system. Clinical data management will be performed in accordance with applicable GSK standards and data cleaning procedures.

14. REFERENCES

Arranz M, Collier D, Sodhi M, Ball D, Roberts G, Price J, Sham P, Kerwin R. Association between clozapine response and allelic variation in 5-HT2A receptor gene. *Lancet*. 1995; 346:281-2.

Executive Summary of the Third Report of the National Cholesterol Education Program (NCEP) Expert Panel on Detection, Evaluation, and Treatment of High Blood Cholesterol in Adults (Adult Treatment Panel III). *JAMA.* 2001;285:2486-97.

GlaxoSmithKline Document Number ZM2004/00059/01. Investigator Brochure, SB-659032.

Macphee CH, Moores KE, Boyd HF et al. 1999. Lipoprotein associated phospholipase A2, platelet activating factor acetylhydrolase, generates two bioactive products during the oxidation of low density lipoprotein. Use of a novel inhibitor. *Biochem.J.*, 338, 479-487.

Morrow JD, Roberts LJ. Chapter 26 – Lipid-derived Autacoids. Goodman and Gilman's Pharmacological Basis of Therapeutics. 10th Edition, 2001.

Packard CJ, O'Reilly DJ, Caslake MJ ET AL. 2000. Lipoprotein associated phospholipase A2 as an independent predictor of coronary heart disease. New Eng.J.Med., 343, 1148-1155.

Ross R. 1999. Atherosclerosis: an inflammatory disease. N Eng J Med, 340, 115-126.

Stafforini DM, McIntyre TM, Zimmerman GA, Prescott SM. Platelet-activating factor acetylhydrolases. J Biol Chem. 1997 Jul 18;272(29):17895-8.

Tew DG, Southan C, Rice SQJ et al. 1996. Purification, properties, sequencing and cloning of a lipoprotein associated serine dependant phospholipase involved in the oxidative modification of low density lipoproteins. Arterioscler.Throm.Vasc.Biol., 16, 591-599.

Appendices

Appendix 1: Time and Events Table

Study Day	Screening	-2	-1	1	2	3	4	5	6	7	8	9	10	11	12	13	14
Admitted to Research Facility		X														X	
Outpatient visits	X				X	X	X	X	X	X	X	X	X	X	X		
Physical examination	X		X														
Prior/Concomitant medications	X		X	X		X	X	X	X	X	X	X	X	X	X	X	
Baseline signs/symptoms or AEs			X	X[2]	X[1]	X[1]	X[1]	X[1]	X[1]	X[1]	X[1]	X[1]	X[1]	X[1]	X[1]	X[1]	X[1,4]
Vital Signs	X			X[1]						X[1]							X[1]
Clinical Lab test (see Section 6.2.4)	X		X[3]														X[1]
12-Lead ECG	X			X[1]						X[1]							X[4]
Urine Drug/Cotinine Screen	X		X													X	
Alcohol Breath Test	X		X														
Exhaled Carbon Monoxide	X		X													X	
Pre-dose urine collection			X														
Rilapladib administration				X	X	X	X	X	X	X	X	X	X	X	X	X	X
Blood Sample -- PK Analysis				X[5]													X[5]
24 hr Urine collection for metabolite analysis																	X
Blood Sample -- Lp-PLA$_2$ Analysis				X[5]													X[5]
Blood Sample -- platelet count & MPV				X[5]													X[5]
Blood Sample -- Platelet Aggregation	X[7]		X[5]	X[5]													X[5]
Blood Sample--Platelet Surface Immunoglobulin				X													
Blood Sample -- PGx Analysis[6]				X													
Meal(s) Served (see Section 7.1)			X	X	X	X	X	X	X	X	X	X	X	X	X	X	X
Discharge from Research Facility				X													X

1. Prior to dosing with study medication
2. Pre-dose and at 6, 10 and 21 hours post-dose
3. Results must be reported prior to dosing.
4. Prior to discharge from the research facility
5. At the nominal times of 0 and 6 hours (relative to dosing, and before meal at each timepoint)
6. May be collected at any time during the study after the PGx informed consent has been obtained.
7. Platelet aggregation will only be done with the highest collagen concentration (10 ug/mL), for purposes of satisfying Exclusion Criteria #21.

Appendix 1: Time and Events Table (cont)

**Study day**	**Day**	**34****	**35**
Admitted to Research Facility	X		
Prior/Concomitant medications	X	X	X
Adverse Events	X	X	X
Vital signs			X
Clinical Lab test (see Appendix 2)			X
12 Lead-ECG			X
Urine Drug Screen	X		
Alcohol Breath Test			
Exhaled Carbon Monoxide	X		
Blood Sample for PK Analysis			X[1]
Blood Sample for Lp-PLA_2 Analysis			X[2]
Blood Sample for platelet count and MPV			X[2]
Blood Sample for Platelet Aggregation		X[1]	X[2]
Blood Sample--Platelet Surface Immunoglobulin			X[1]
Meal(s) Served (see Section 7.1)	X	X	X
Discharge from Research Facility			X

1. At the equivalent times to the predose samples on the dosing days
2. At the equivalent times of 0 and 6 hours post dose on the dosing days

*****This only applies to a subset of subjects, see Section 6.5.1*****	
**Study Day**	**Day 34 (+/- 4 days)**
Admitted to Research Facility	(Evening before)
Prior/Concomitant medications	X
Adverse Events	X
Blood sample for Platelet Aggregation	X[1,2]
Meal(s) Served (see Section 7.1)	X
Discharge from Research Facility	X

1. At the equivalent times to the predose samples on the dosing days
2. On Day 34 only there will be ex-vivo evaluation of the subject plasma and platelets on platelet aggregation when combined with normal (untreated) platelets and normal (untreated) plasma.

Appendix 2: Framingham Point Score

Estimating 10-year Risk <u>For Men</u>

Age	Points
20 – 34	-9
35 – 39	-4
40 – 44	0
45 – 49	3
50 - 54	6
55 – 59	8
60 – 64	10
65 – 69	11
70 – 74	12
75 - 79	13

Total Cholesterol	Points at Ages 20-39	Points at Ages 40-49	Points at Ages 50-59	Points at Ages 60-69	Points at Ages 70-79
<160	0	0	0	0	0
160 – 199	4	3	2	1	0
200 – 239	7	5	3	1	0
240 – 279	9	6	4	2	1
≥280	11	8	5	3	1

	Points at Ages 20-39	Points at Ages 40-49	Points at Ages 50-59	Points at Ages 60-69	Points at Ages 70-79
Nonsmoker	0	0	0	0	0
Smoker	8	5	3	1	1

HDL	Points
≥60	-1
50 – 59	0
40 – 49	1
<40	2

Systolic BP	If Untreated	If Treated
≤120	0	0
120 – 129	0	1
130 – 139	1	2
140 – 159	1	2
≥160	2	3

CALCULATE TOTAL HERE

	Points
Age	
Total Cholesterol	
Smoking Status	
HDL	
Systolic BP	
Total	

Point Total	10 – Year Risk
<0	<1%
0	1%
1	1%
2	1%
3	1%
4	1%
5	2%
6	2%
7	3%
8	4%
9	5%
10	6%
11	8%
12	10%
13	12%
14	16%
15	20%
16	25%
≥17	≥30%

.

Taken from: Executive Summary of the Third Report of the National Cholesterol Education Program (NCEP), 2001.

Appendix 3: Pharmacogenetic Research

Pharmacogenetics – Background

Pharmacogenetics (PGx) is the study of variability in drug response due to hereditary factors in different populations. There is increasing evidence that an individual's genetic composition (i.e., genotype) may impact the pharmacokinetics (absorption, distribution, metabolism, elimination), pharmacodynamics (relationship between concentrations and pharmacologic effects or the time course of pharmacologic effects) and/or clinical outcome (in terms of efficacy and/or safety and tolerability). Some reported examples of PGx analysis include:

Drug	Disease	Gene	Outcome
Abacavir	HIV [Hetherington, 2002 and Mallal, 2002]	HLA (human leukocyte antigen)	Caucasian males with HLA B57 variant were at increased risk for experiencing hypersensitivity to abacavir
Tranilast	Restenosis prevention following coronary bypass [Roses, 2002]	UGT1A1	Drug induced hyperbilirubinemia explained by high proportion of affected patients having 7/7 TA repeat genotype, consistent with clinically benign Gilbert's Syndrome
ABT-761	Asthma [Drazen, 1999]	ALOX5	ALOX5 Sp1 promoter genotype (x,x) associated with reduced response to 5-lipoxygenase inhibitor ABT-761

A key component to successful PGx research is the collection of samples during the conduct of clinical studies.

Collection of whole blood samples, even when no a priori hypothesis has been identified, may enable PGx analysis to be conducted if at any time it appears that there is a potential unexpected or unexplained variation in handling or response to rilapladib.

Pharmacogenetic Research Objectives

The objective of the PGx research (if there is a potential unexpected or unexplained variation) is to investigate a possible genetic relationship to handling or response to rilapladib. If at any time it appears there is potential variability in response in this clinical study or in a series of clinical studies with rilapladib that may be attributable to genetic variations of subjects, the following objectives may be investigated:

- Relationship between genetic variants and the pharmacokinetics of rilapladib
- Relationship between genetic variants and safety and/or tolerability of rilapladib

Study Population

Any subject who has given informed consent to participate in the clinical study, has met all the entry criteria for the clinical study, and receives investigational product may take part in the PGx research. Any subject who has received an allogeneic bone marrow transplant must be excluded from the PGx research.

Subject participation in the PGx research is voluntary and refusal to participate will not indicate withdrawal from the clinical study. Refusal to participate will involve no penalty or loss of benefits to which the subject would otherwise be entitled.

Study Assessments and Procedures

In addition to any blood samples take for the clinical study, a whole blood sample (~10ml) will be collected for the PGx research using a tube containing EDTA. The PGx sample is labeled (or "coded") with a study specific number that can be traced or linked back to the subject by the investigator or site staff. Coded samples do not carry personal identifiers (such as name or social security number). The blood sample will be taken on a single occasion unless a duplicate sample is required due to inability to utilize the original sample. It is recommended that the blood sample be taken at the first opportunity after a subject has been randomized and provided informed consent for PGx research, but may be taken at any time while the subject is participating in the clinical study.

If deoxyribonucleic acid (DNA) is extracted from the blood sample, the DNA may be subjected to sample quality control analysis. This analysis will involve the genotyping of several genetic markers to confirm the integrity of individual samples. If inconsistencies are noted in the analysis, then those samples may be destroyed.

The need to conduct PGx analysis may be identified after a study (or a set of studies) of rilapladib has been completed and the study data reviewed. For this reason, samples may be kept for up to 15 years after the last subject completes the study or GSK may destroy the samples sooner. In special cases, the samples may not be studied. This might happen if there are not enough subjects, if the study is stopped for other reasons, or if no questions are raised about how people respond to rilapladib. GSK or those working with GSK (for example, other researchers) will only work with samples collected from the study for the use stated in this protocol and in the informed consent form. Samples will be stored securely. Subjects can request their sample to be destroyed at any time.

Subject Withdrawal from Study

If a subject who has consented to participate in PGx research withdraws from the clinical study for any reason other than lost to follow-up, the subject will be given the following options concerning the PGx sample, if already collected:

- PGx research continues per the subject's consent; or,
- Any remaining sample is destroyed

If a subject withdraws consent from the PGx research or requests sample destruction, the investigator must complete the appropriate documentation to request sample destruction

within the timeframe specified by GSK and maintain the documentation in the site study records. In either case, GSK will only keep study information collected/generated up to that point.

Screen and Baseline Failures

If a blood sample for PGx research has been collected and it is determined that the subject does not meet the entry criteria for participation in the clinical study, then the investigator must complete the appropriate documentation to request sample destruction within the timeframe specified by GSK and maintain the documentation in the site study records.

Pharmacogenetics Analyses

The need to conduct PGx analysis may be identified after a study (or set of studies) of rilapladib has been completed and the study data reviewed. For this reason, samples may be kept for up to 15 years after the last subject completes the study or GSK may destroy the samples sooner. In special cases, the samples may not be studied. This might happen if there are not enough subjects, if the study is stopped for other reasons, or if no questions are raised about how people respond rilapladib.

Generally GSK will utilize two approaches to explore genetic variation in drug response.

1. Specific sections of DNA may be selected from areas of the genome (e.g., candidate genes) known to encode the drug target, drug metabolizing enzymes, areas associated with mechanisms underlying adverse events, and those linked to study disease and, thus, linked to drug response.
2. By evaluating large numbers of polymorphic markers (e.g., single nucleotide polymorphisms or SNPs) throughout the genome, sets of markers may be identified that correspond to differential drug response.

Hardy-Weinberg Equilibrium Testing

The genotypic frequencies of each polymorphism will be evaluated for conformity to those expected under normal conditions by employing Hardy-Weinberg Equilibrium testing.

Comparison of Demographic and Baseline Characteristics by Genotype

Differences in baseline clinical characteristics and potential contributing covariates may be summarized and compared among genotype (or haplotype) subgroups.

Evaluation of Genotypic Effects

Analyses may be carried out to evaluate the degree of association between subject genotype (or haplotype) and selected parameters (e.g., pharmacokinetics, efficacy and safety). Where such genotypic tests are inappropriate (for example, where the number of marker genotypes is too large and/or the frequency of individual genotypes too small),

allelic tests may be conducted. Allelic tests evaluate whether the frequency of each marker allele is the same in responders and non-responders.

Evaluation of Treatment by Genotype and Gene-Gene Interaction

In addition to evaluating the main effects of the genotypes (haplotypes or alleles) on the selected parameters, the possibility of a treatment group by genotype (haplotype or allele) interaction will also be explored. If appropriate, the joint effects of multiple markers (gene-gene interactions) may also be evaluated.

Linkage Disequilibrium

For pairs of polymorphisms, the degree to which alleles from the two sites are correlated (linkage disequilibrium) may also be evaluated. If the genotypes at two polymorphic sites within a gene are shown to be statistically associated with a response to investigational product, the degree of linkage disequilibrium will aid interpretation in that it will indicate the extent to which the two sites are exerting independent effects.

Multiple Comparisons and Multiplicity

To the extent that multiple markers are evaluated (especially in the case of a genome scan for association), an adjustment to observed p-values may be made to limit erroneous conclusions due to multiple tests.

Power and Sample Size Considerations

The ability to detect differential drug response among genotypes at a polymorphic site depends on the total number of subjects genotyped and the frequency distribution of the different genotypes. Consequently, genotyping analyses are plausible for those polymorphic sites where the number of subjects comprising the genotypic groups is sufficiently large; however, these frequencies will not be known until sufficient samples have been collected and genotyping is complete.

Estimates of sample sizes required to demonstrate genotype effects vary considerably, depending on the assumptions made about allele frequency, genetic effect size, and mechanism of inheritance [Cardon, 2000]. In the work by Palmer and Cookson [Palmer, 2001], which assumed a genotype relative risk of 1.5, it was estimated that more than 300 cases and 600 controls would be needed to conduct a genetic association analysis. In contrast, McCarthy and Hilfiker [McCarthy, 2000] showed that with a genotype relative risk of 2.16 and a relatively commonly occurring genotype, only 30 cases and 30 controls would be needed to demonstrate an association.

Published PGx examples include abacavir hypersensitivity reaction [Hetherington, 2002 and Mallal, 2002] and tranilast induced hyperbilirubinemia [Roses, 2002] where genetic markers have been found to significantly associate with hypersensitivity reaction (abacavir) and hyperbilirubinemia (tranilast). These examples show that small sample sizes typically encountered in Phase I and Phase II studies may be sufficient to identify clinically relevant genetic associations.

Informed Consent

Subjects who do not wish to participate in the PGx research may still participate in the clinical study. PGx informed consent must be obtained prior to any blood being taken for PGx research.

Provision of Study Results and Confidentiality of Subject's PGx Data

GSK may summarize the cumulative PGx research results in the clinical study report.

In general, GSK does not inform the investigator, subject, or anyone else (e.g., family members, study investigators, primary care physicians, insurers, or employers) of the PGx research results because the information generated from PGx studies is preliminary in nature, and the significance and scientific validity of the results are undetermined at such an early stage of research, under any circumstances unless required by law.

References

Cardon LR, Idury RM, Harris TJR, Witte JS, Elston RC. Testing drug response in the presence of genetic information: sampling issues for clinical trials. *Pharmacogenetics*. 2000; 10:503-10.

Drazen JM, Yandava CN, Dube L, Szcerback N, Hippensteel R, Pillari A, Israel E, Schork N, Silverman ES, Katz DA, Drajesk J. Pharmacogenetic association between ALOX5 promoter genotype and the response to anti-asthma treatment. *Nature Genet*. 1999; 22:168-70.

Hetherington S, Hughes AR, Mosteller M, Shortino D, Baker KL, Spreen W, Lai E, Davies K, Handley A, Dow DJ, Fling ME, Stocum M, Bowman C, Thurmond LM, Roses AD. Genetic variations in HLA-B region and hypersensitivity reactions to abacavir. *Lancet*. 2002; 359:1121-2.

Mallal S, Nolan D, Witt C, Masel G, Martin AM, Moore C, Sayer D, Castley A, Mamotte C, Maxwell D, James I. Association between presence of HLA-B*5701, HLA-DR7, and HLA-DQ3 and hypersensitivity to HIV-1 reverse-transcriptase inhibitor abacavir. *Lancet*. 2002; 359:727-32.

McCarthy JJ, Hilfiker R. The use of single-nucleotide polymorphism maps in pharmacogenomics. *Nat Biotechnol*. 2000; 18:505-8.

Palmer LJ, Cookson WO. Using single nucleotide polymorphisms as a means to understanding the pathophysiology of asthma. *Respir Res*. 2001; 2:102-12.

Roses AD. Genome-based pharmacogenetics and the pharmaceutical industry. *Nat Rev Drug Discov*. 2002; 1:541-9.
